# Supplementary material for: Setting the boundaries–an approach to estimate the Loss Gap in dairy cattle
Source: PLoS One. 2024 Jun 27;19(6):e0306314. doi: 10.1371/journal.pone.0306314 (PMC11210862; doi:10.1371/journal.pone.0306314)
Supplement: S1 Table — (DOCX) [file pone.0306314.s001.docx]

Table S1. Classification system and cost centres descriptors across the selected relevant references

| Parameter | Reference | | | | | | |
| --- | --- | --- | --- | --- | --- | --- | --- |
|  | Redman et al (2020) ([1](#_ENREF_1)) | Beattie et al (2019)([2](#_ENREF_2)) | King et al (2020)([3](#_ENREF_3)) | AHDB (2020)([4](#_ENREF_4)) | Hyde et al (2020)([5](#_ENREF_5)) | Hanks and Kossaibati (2020) ([6](#_ENREF_6)) | Boulton (2015) ([7](#_ENREF_7)) |
| Publication type | Budgeting book | Budgeting book | Budgeting book | Report | Scientific paper | Report | PhD thesis |
| Data sources | Farm Business Surveys in the UK; Levy-boards; Benchmarking systems; Office of National Statistics; Private consultancy activities | Farm Business Surveys in the UK; Levy-boards; Benchmarking systems; Office of National Statistics; Private consultancy activities | Farm Business Surveys in the UK; Levy-boards; Benchmarking systems; Office of National Statistics; Private consultancy activities | Farm annual accounts sourced from Promar International and partners | Birth and death data from 2010 to 2019 from the British Cattle Movement Services | Monthly milk records obtained by National Milk Records (NMR) | Primary data |
| Sample size | Not reported | Not reported | 161 farms for England and Wales | 350 farms | Whole population | 500 farms | 102 farms |
| Sampling strategy | Random proportional stratified sampling of farms with at least 25,000 euros of output according to type and geographical location. | Random proportional stratified sampling of farms with at least 25,000 euros of output according to type and geographical location. | Random proportional stratified sampling of farms with at least 25,000 euros of output according to type and geographical location. | Random stratified sampling according to geographical location, level of milk production, calving pattern, housing period, type of contract, financial and physical performance | None | Randomly selection of farms with fully milk record on a monthly assisted basis with a minimum of two-year records. | Proportional convenience sampling according to county |
| No of categories for dairy systems according to calving pattern | 3 | 2 | 3 | 3 | N/A^*^ | 1 | 4 |
| Classification of dairy systems | Spring calving, Autumn calving, all-year-round calving | Spring calving and all-year-round calving | Spring calving, Autumn calving, all-year-round calving | Spring calving, Autumn calving, all-year-round calving | N/A | All-year-round calving | Spring calving, Autumn calving, all-year-round calving, multi block calving |
| Categories within dairy systems | Two categories according to yearly yield per cow – average and high | Three categories for AYR calving systems according to yearly yield per cow and milk contract type | Averages | Three categories according to quartiles with farms ranked by output/input ratio | N/A | Three categories according to quartiles with farms ranked by performance for individual parameters | Averages with standard deviations |
| Results presentation | Ppl^**^, £ per head | Ppl, £ per head | Ppl, £ per head | Ppl, £ per head | % | % or number according to parameter | £ per head |
| Sensitivity analysis | Impact on gross margin per cow with 0.25 and 0.5 pence change per litre on milk price and 10 and 20£ change per tonne in concentrate price | Impact on gross margin per cow with 1 pence change per litre on milk price and 10£ change per tonne in concentrate price | Impact on gross margin per cow with change in concentrate use and yearly milk yield level per cow; impact on gross margin per hectare with change in pence per litre on milk price and stocking rate | None | None | None | None |
| Young stock costs | Takes into account herd depreciation, and value of culled cows and calves adjusted for casualty and mortality allowance) | Takes into account the herd depreciation, and value of culled cows and calves adjusted for casualty and mortality allowance) | Takes into account the herd depreciation, and value of culled cows and calves adjusted for casualty and mortality allowance) | Takes into account the average value of cows and heifers entering the herd, the average culling value of cows exiting the herd, and the number of animals exiting the herd | N/A | N/A |  |
| ^*^N/A stands for non-applicable ^**^ ppl stand for pence per litre | | | | | | | |

1. Redman G. John Nix farm management pocketbook. Melton Mowbray: Agro Business Consultants Ltd; 2020.

2. Beattie A. The Farm Management Handbook 2019/20 2019. Available from: <https://www.fas.scot/publication/farm-management-handbook-2019-20/>.

3. King R, Benbow A, Ingamells C, Redman G. The Agricultural Budgeting & Costing Book 90th Edition. 2020.

4. AHDB Dairy. Dairy performance results 2018/19. 2020.

5. Hyde RM, Green MJ, Sherwin VE, Hudson C, Gibbons J, Forshaw T, et al. Quantitative analysis of calf mortality in Great Britain. Journal of Dairy Science. 2020;103(3):2615-23.

6. Hanks J, Kossaibati M. Key Performance Indicators for the UK national dairy herd - A study of herd performance in 500 Holstein/Friesian herds for the year ending 31st August 2020. Veterinary Epidemiology & Economics Research Unit - University of Reading; 2020.

7. Boulton A. An economic analysis of heifer rearing and breeding selection in Great Britain – an empirical analysis: The Royal Veterinary College; 2015.
