# Supplementary material for: Setting the boundaries–an approach to estimate the Loss Gap in dairy cattle
Source: PLoS One. 2024 Jun 27;19(6):e0306314. doi: 10.1371/journal.pone.0306314 (PMC11210862; doi:10.1371/journal.pone.0306314)
Supplement: S2 Table — (DOCX) [file pone.0306314.s002.docx]

Table S2. Descriptors for the different costs centres across the selected relevant references with data on industry enterprise budget

| Cost Centre | Reference | | | |
| --- | --- | --- | --- | --- |
|  | Redman et al (2020) ([1](#_ENREF_1)) | Beattie et al (2019) ([2](#_ENREF_2)) | King et al (2020) ([3](#_ENREF_3)) | AHDB (2020) ([4](#_ENREF_4)) |
| Variable costs | | | | |
| Artificial insemination | No information | No information | No information | N/A^*^ |
| Bedding | No information | No information | Different assumptions depending on the calving pattern | N/A |
| Concentrate feed | No information | No information | No information | No information |
| Forage | Includes fertilisers, seeds and sprays | No information | Includes fertilisers and reseeding costs | No information |
| Other livestock costs | Includes recording, parlour consumables and sundries | Includes milk recording, bedding, sawdust and dairy detergents. | Includes milk recording/costings, dairy chemicals, ear tags, freeze branding, etc | Includes artificial insemination and breeding costs, bedding  costs and dairy, parlour and youngstock sundries |
| Purchased bulk feed | No information | No information | Includes by-products such as brewers grains, etc | No information |
| Vet services and medicines | No information | No information | Includes vaccinations, wormers, insect spray and general veterinary care | No information |
| Fixed costs | | | | |
| Depreciation | N/A | Includes machinery and property depreciation | N/A | N/A |
| General overheads | Includes general farm maintenance and repairs, office expenses, water, insurance, fees, subscriptions, etc | Includes property repairs, rates, insurance, and miscellaneous | Includes land and building repairs, water, office costs, insurance, etc | Overheads of the dairy and youngstock herd, including inputed cost of unpaid labour and machinery and buildings depreciation, and other operation costs (water and telephone charges, general insurances, professional fees and other office-related costs) but excluding rent and finance |
| Labour | Includes unpaid labour but there is not management charge included | Regular labour | Includes unpaid labour at 3.5 ppl^**^ of the total labour charge | Actual cost of paid labour, plus imputed cost for unpaid labour |
| Power and machinery | Includes all machinery and equipment costs, including the use of farm vehicles, depreciation, fuel, servicing, etc | Includes repairs, fuel (including drying fuel), oil, electricity, contracting, crop and livestock haulage and leasing, and hire | Includes depreciation and repairs on milking plant and equipment, electricity, fuel and contracting | Includes repairs and spares, machinery hire, contracting, fuel, electricity and vehicle tax and insurance, and depreciation on dairy-specific and forage machinery and equipment |
| Rent and finance charges | Assumes modest rental charge on all land plus interest charges on a small amount of working capital | Includes interest (bank, HP and loans) and rent (excluding keep) | Varies depending on the business model (tenanted vs owned land and level of bank borrowings) | Includes rented land and an opportunity costs for owned land, and financial costs related to borrowing |
| *N/A stands for non-applicable ** ppl stand for pence per litre | | | | |

1. Redman G. John Nix farm management pocketbook. Melton Mowbray: Agro Business Consultants Ltd; 2020.

2. Beattie A. The Farm Management Handbook 2019/20 2019. Available from: <https://www.fas.scot/publication/farm-management-handbook-2019-20/>.

3. King R, Benbow A, Ingamells C, Redman G. The Agricultural Budgeting & Costing Book 90th Edition. 2020.

4. AHDB Dairy. Dairy performance results 2018/19. 2020.
