# Supplementary material for: Setting the boundaries–an approach to estimate the Loss Gap in dairy cattle
Source: PLoS One. 2024 Jun 27;19(6):e0306314. doi: 10.1371/journal.pone.0306314 (PMC11210862; doi:10.1371/journal.pone.0306314)
Supplement: S3 Table — (DOCX) [file pone.0306314.s003.docx]

**Table S3. Farm Enterprise Budget for each category of the production systems (results in thousands)**

| Dairy System | Scenario | Farm Enterprise Budget in thousands (`000) | | | |
| --- | --- | --- | --- | --- | --- |
|  |  | Top 25% | Middle 50% | Bottom 25% | Total |
| Spring calving | Baseline | £15,539 | £19,151 | £310 | £35,000 |
|  | Utopia 1 | £16,139 | £22,119 | £1,091 | £39,349 |
|  | Utopia 2 | =* | £21,930 | £642 | £38,711 |
|  | Utopia 3 | = | £24,756 | £2,625 | £43,520 |
| Autumn calving | Baseline | £24,032 | £16,654 | -£1,079 | £39,607 |
|  | Utopia 1 | £25,492 | £21,399 | £1,771 | £48,662 |
|  | Utopia 2 | = | £28,523 | £5,646 | £59,662 |
|  | Utopia 3 | = | £24,058 | £5,520 | £55,071 |
| AYR calving | Baseline | £185,985 | £59,779 | -£55,924 | £189,840 |
|  | Utopia 1 | £227,252 | £136,481 | -£39,038 | £324,695 |
|  | Utopia 2 | = | £147,089 | -£22,064 | £352,277 |
|  | Utopia 3 | = | £170,184 | -£4,494 | £392,942 |
| All systems | Baseline | £225,556 | £95,584 | -£56,693 | £264,447 |
|  | Utopia 1 | £268,883 | £179,999 | -£36,175 | £412,706 |
|  | Utopia 2 | = | £197,542 | -£15,775 | £450,650 |
|  | Utopia 3 | = | £218,998 | £3,651 | £491,533 |
| *the = sign means that the estimate is equal to that of Utopia 1 | | | | | |
