# Supplementary material for: Setting the boundaries–an approach to estimate the Loss Gap in dairy cattle
Source: PLoS One. 2024 Jun 27;19(6):e0306314. doi: 10.1371/journal.pone.0306314 (PMC11210862; doi:10.1371/journal.pone.0306314)
Supplement: S4 Table — (DOCX) [file pone.0306314.s004.docx]

Table S4. Loss Gap estimate for different models

| Parameter | Utopia | | |
| --- | --- | --- | --- |
|  | 1 | 2 | 3 |
| Loss Gap (thousands) | £148,260 | £186,203 | £227,086 |
| Proportional variation* | - | 25.6% | 53.2% |
| *Proportional variation from Utopia 1 | | | |
