# Supplementary material for: Setting the boundaries–an approach to estimate the Loss Gap in dairy cattle
Source: PLoS One. 2024 Jun 27;19(6):e0306314. doi: 10.1371/journal.pone.0306314 (PMC11210862; doi:10.1371/journal.pone.0306314)
Supplement: Attachment — Submitted filename: NMR500Herds-2023V2.pdf [file pone.0306314.s005.pdf]

# Key performance indicators (KPIs) for the UK national dairy herd

# A study of herd performance in 500 Holstein/Friesian NMR recorded herds for the year ending 31st August 2023

**Dr James Hanks**
**Dr Emma Taylor**
**Dr Mohamad Kossaibati**

**January 2024**

**Veterinary Epidemiology & Economics Research Unit (VEERU),**
**School of Agriculture Policy & Development,**
**University of Reading,**
**P.O.Box 237**
**Reading,**
**RG6 6AR**
**panveeru@panveeru.net**

University of Reading

# Table of Contents


**Table of Figures** ..... ii

**Table of Tables** ..... iv

**Executive Summary** ..... 1

**Further Reading** ..... 3

**Acknowledgements** ..... 3

**Section 1: Description of the study and methods used** ..... 4

1.1 Introduction ..... 4

1.2 The sample of herds ..... 4

1.3 Description of parameters ..... 5

1.4 Changes to studies from earlier years ..... 7

**Section 2: KPI results for the year ending 31 August 2023** ..... 8

**Section 3: Trends in KPIs from 2010 to 2023** ..... 28

3.1 Summary of trends since 2010. ..... 28

3.2 Trends in herd size since 2010. ..... 31

3.3 Trends in culling and longevity parameters since 2010. ..... 32

3.4 Trends in key fertility parameters since 2020. ..... 34

3.5 Trends in milk production parameters since 2010. ..... 39

3.6 Trends in SCC parameters and mastitis rate since 2010 (SCC) or 2016 (mastitis). ..... 40

**Section 4. The practical use of KPIs by farmers and their technical advisers** ..... 43

4.1 Interpreting the “KPIs at a glance” report in InterHerd+ ..... 43

4.2 Using the target and range values to highlight a herd’s strengths and weaknesses ..... 45

4.3 Correlation between Herd SCC vs % chronic high SCC milk samples ..... 46

**Conclusion.** ..... 48

**Appendix 1. Changes in KPIs between 2010 and 2023** ..... 49

**Appendix 2. KPI definitions** ..... 51

**Appendix 3. Herd Companion high SCC categories** ..... 54

# Table of Figures

## Introductory Figures

Figure i Herd size of the 500 herds. ....................5
Figure ii A description of the median, inter-quartile range and target values. ....................6

## KPI 2023 Figures

A. Cull/death/exit rate (%) .................... 10
B. Cull/death/exit rate within the first 100 days after calving .................... 10
C. Average age (in years) at exit. .................... 11
D. Average age (in lactations) at exit .................... 11
E. % Served 80 days after calving .................... 12
F. % Conceived 100 days after calving. .................... 12
G. Calving to 1st service interval (days) .................... 13
H. Calving interval (days) .................... 13
I. Age at 1st calving (years). .................... 14
J. Conception rate .................... 14
K. Heat detection .................... 15
L. % service intervals >50 days. .................... 15
M. Submission rate. .................... 16
N. Pregnancy rate .................... 16
O. Lifetime milk / cow / day (kg) .................... 17
P. Milk / cow / year (kg). .................... 17
Q. Average protein (%). .................... 18
R. Average fat (%). .................... 18
S. 305 day yield (kg). .................... 19
T. 305 day protein (kg) .................... 19
U. 305 day fat (kg). .................... 20
V. Average SCC (‘000 cells/ml). .................... 20
W. % of milk samples with high SCC .................... 21
X. % of milk samples with SCC ≥ 500,000 cells/ml .................... 21
Y. % 1st recording SCC ≥ 200,000 cells/ml .................... 22
Z. Percentage chronic SCC ≥ 200,000 cells/ml .................... 22
ZA. Dry period cure (High:Low) .................... 23
ZB. Dry period protection (Low:Low) .................... 23
ZC. % Low at the end of previous lactation. .................... 24
ZD. % NEW SCC milk samples. .................... 24
ZE. % dried-off with no SCC ≥ 200,000 cells/ml .................... 25
ZF. Threshold Index new high / new low .................... 25
ZG. Recovery % of New/First/Repeat infections .................... 26
ZH. Recovery % of chronic infections .................... 26
ZJ. Mastitis rate (%). .................... 27

## KPI 2010 to 2023 Trends Figures

Figure 1. Herd size (number of cows). ........ 31
Figure 2. % of cows which exited the herd (culled, died or sold) (%). ........ 32
Figure 3. % of cows which exited the herd (culled, died or sold) within 100 DPP (%). ........ 32
Figure 4. Age at exit (years). ........ 33
Figure 5. Age at exit (lactations). ........ 33
Figure 6. Age at first calving (years). ........ 34
Figure 7. Age at first calving (days). ........ 34
Figure 8. Productive life (age at exit - age at 1st calving) (days). ........ 35
Figure 9. % served by 80 days after calving. ........ 35
Figure 10. % conceived 100 days after calving. ........ 36
Figure 11. Heat detection (%) ........ 36
Figure 12. Conception rate (%). ........ 37
Figure 13. Submission rate (%). ........ 37
Figure 14. Pregnancy rate (%) ........ 38
Figure 15. Calving interval (days). ........ 38
Figure 16. Lifetime milk per cow / day (kg) ........ 39
Figure 17. Milk per cow / year (kg). ........ 39
Figure 18. Herd average SCC (0’000 cells/ml). ........ 40
Figure 19. % milk samples originating from chronic (repeat) high SCC cows. ........ 40
Figure 20. % dried-off with no high SCC in lactation. ........ 41
Figure 21. Mastitis rate (cases / 100 cows in milk / year) ........ 41
Figure 22. Mastitis rate (cases / 100 cows in milk / year) ........ 42

## Supplementary Figures

Supplementary Figure 1. The “KPIs at a glance” report in InterHerd+. ........ 43
Supplementary Figure 2. Interpreting the “KPIs at a glance” report in InterHerd+. ........ 44
Supplementary Figure 3. Highlighting the strengths and weaknesses of a dairy herd. ........ 45
Supplementary Figure 4. The relationship between herd average SCC and the % chronic high SCC cows. ........ 46
Supplementary Figure 5. The relationship between herd average SCC and mastitis incidence. ........ 47

# Table of Tables

## KPI 2023 Tables

Table KPIs A. Summary of culling, fertility and milk related KPIs derived from the analysis of 500 NMR milk recording herds for the year ending 31 August 2023. ....8
Table KPIs B. Summary of the somatic cell count (SCC) and mastitis related KPIs derived from analysis of 500 NMR milk recording herds for the year ending 31 August 2023. ....9

## KPI 2010 to 2023 Trends Tables

Table Trends 1. Comparison of longevity and fertility median and target values derived from the study of 500 NMR recording herds in 2022 and 2023 with the original study in 2010. .... 29
Table Trends 2. Comparison of milk, composition and SCC median and target values derived from the study of 500 NMR recording herds in 2022 and 2023 with the original study in 2010. .... 30

## Supplementary Tables

Supplementary Table 1. Percentage of herds with low (<10%) and high (≥15%) levels of chronic high SCC samples, in the KPI studies from 2010 to 2022. .... 47

## Appendices Tables

Table Appendix 1. A comparison of the 2010 and 2013 median values within the 500-herds and 100 common herds. .... 50
Table Appendix 2. KPI definitions. .... 51

# Executive Summary

This is the fourteenth annual study of key performance indicators (KPIs) using a cross-section of 500 dairy herds that milk record with National Milk Records (NMR). In addition to allowing direct comparison of performance for any milk recording herd, these studies provide a description of both **current performance** and **long term trends** in the national dairy herd.

In 2023, in comparison to earlier studies, cows were markedly more fertile and with higher milk yields and significantly lower somatic cell counts (SCC)/fewer cases of mastitis. Many parameters including; conception, heat detection, age at first calving, 305-day milk yield and SCC have improved almost every year since the first study in 2010. However, culling rates have increased, so cows are being removed from the herd at an **earlier age** and after completing **fewer lactations**. Subsequently, despite substantial improvements in milk production and fertility parameters, overall **cow longevity has failed to improve.**

Two important measures of overall efficiency have stalled in recent years. The 2023 median values for **milk/cow/day of life** and **milk/cow place/year** were similar to the 2019 median values. Therefore, **the benefits of keeping more mature cows in production may now exceed the gains being achieved from genetic improvement of first lactation cows joining the herd.**

**Herd size: Herd sizes have remained relatively stable for the last five years.**

- The median **herd size** was **178 cows** (Figure i). One herd in four had >243 cows and one herd in four had <123 cows.
- Since 2010, the median herd size has increased by 49 cows, but herd sizes have not changed markedly since 2019 (Figure 1).

**Longevity: Culling rates increased slightly to 28%, but the number of lactations at exit remained unchanged at 3.6 lactations.**

- The median **age at 1st calving** was 804 days/**2.2 years** (I), an increase of 5 days compared to 2022 (799 days) but decrease of 89 days compared to 2010 (893 days) (Figure 7).
- The median **age at exit** was **6.0 years** (2,179 days) (C), almost unchanged from 2022, but 214 days earlier than in 2010 (Figure 6).
- The median **productive life** (age at exit - age at 1st calving) was **1,367 days** / 3.7 years, 144 days shorter than in 2010 (Figure 8).
- The median **lactations per cow at exit** was **3.6** (D), unchanged from 2022 but 0.3 lactations less than in 2010 (Figure 5).
- The median **culling rate** increased to **28%** (A). Culling rates have remained relatively constant in recent years though generally 3 - 4% higher than in 2010-2015 (Figure 2).

**Fertility: The majority of fertility parameters have gradually improved since 2010.**

- The median **calving interval** was **394 days** (H), unchanged from 2022, but 30 days shorter than in 2010 (Figure 15).
- The median **conception rate** was **39%** (J), an increase of 1% on 2022. Variation in conception rates between herds remained high, ranging from below 32% in the bottom quartile of herds to over 45% in the best quartile. Over the past 14 years, the median conception rate has increased by 7% (Figure 12).

- The median rate of **heat detection** was **41%** (K)**.** Although unchanged from 2022, heat detection has increased by 11% since 2010. The median herd % of inter-service intervals that are >50 days (suggesting 2+ missed oestrus cycles) was 13% lower than in 2010, showing that heat detection has improved significantly in recent years (Figure 11).
- The median **submission** and **pregnancy rates** were **41%** and **16%** respectively (M, N) improvements of 14% and 7% respectively compared to 2010 (Figure 13, Figure 14).
- The median **% cows served by day 80** (after calving) was **59%** (E), 13% higher than in 2010, with most improvements occurring before 2015 (Figure 9).
- The median **% conceived by day 100** (after calving) was **39%** (F), 13% higher than in 2010 (Figure 10).

**Milk production: Milk yields have remained stable for the last five years although protein and fat contents have increased slightly.**

- The median **milk production / cow / year** was **8,737 kg** (P). Although >1,000 kg greater than in 2010, it has remained largely unchanged since 2019 (Figure 17).
- The median **lifetime milk / cow / day** (since birth) was **12.7 kg** (O) and has remained largely unchanged since 2019 (Figure 16).
- The median **protein** content of milk was **3.36%** (Q), compared to 3.33% in 2022 and 3.27% in 2010. The protein yield (kg / cow / year) was 294 kg an increase of 17% since 2010.
- The median **fat %** content of milk was **4.26%** (R), compared to 4.18% in 2022 and 3.96% in 2010. The fat yield (kg / cow / year) was 372 kg an increase of 22% since 2010.

**Somatic cell count (SCC):**

- The median **herd SCC** was **168,000 cells/ml milk** (V), continuing the long-term downward trend (Figure 18).
- **Herd SCC was below 200,000 cells/ml milk in 70% herds**, up from 44% of herds in 2010.
- The median level of **chronic (repeat) high SCC cows** was **8%**, compared to 14% in 2010, emphasising enormous progress in milk quality (Figure 19).
- **Half the herds recorded 51% or more of their cows completing lactations without any high SCC recordings** (ZE). In 2010, the equivalent figure was 33% of cows (Figure 20).
- **52% of all cows completed lactations without recording a single high SCC** (>200,000 cells/ml milk). In 2010, the equivalent value was 35% of cows.

**Mastitis:** Cases are not consistently recorded across all 500 herds. Since 2016, a subset of the 500 herds (recording more than 5 cases/100 cows/year) has been included for analysis of mastitis incidence. In 2023, this included 242 herds.

- **Mastitis incidence** in the median herd in the 2023 subset was **22 cases per 100 cows per year** (ZJ), a reduction of 14 cases per 100 cows per year since 2016 (Figure 21).
- **In half the herds** (with mastitis records), **85% of cows recorded no mastitis** in completed lactations, an increase from 79% in 2016.

# Further Reading

Milk recording focused research:

- Taylor, E. N., Channa, K., Hanks, J., Taylor, N. 2023. Milk recording data indicates the importance of fertility, including age at first calving, on the progression of first lactation cows to second lactation. [Under peer review].
- Taylor, E. N., Kossaibati, M., Hanks, J., Taylor, N. 2023. How are fertility, production and health parameters changing in the national UK dairy herd? Trends in KPIs since 2010. *British Cattle Veterinary Association Congress 2023*. Telford: 19th - 21st October 2023.

Johne's Disease focused research: Milk recording data, alongside milk ELISA data, are used to calculate a series of Johne's Disease (JD) parameters within the JD tracker.

- Hanks, J., Orpin, P.G., Taylor, E. N., Morrison, R. 2023. Practical experiences in using disease data to promote farmer and vet engagement in Johne's Disease control - The development and impact of the Johne's Tracker. *British Cattle Veterinary Association Congress 2023*. Telford: 19th - 21st October 2023.
- Orpin, P.G., Sibley, D., Hanks, J., Taylor, N. 2022. The National Johne's Management Plan: Using risk and data analysis to optimize Johne's control in dairy herds. Livestock. 27, 1, 2 - 7. https://doi.org/10.12968/live.2022.27.1.24


# Acknowledgements

The authors are very grateful to National Milk Records (NMR) for their assistance and cooperation with the preparation of this study.

The authors also acknowledge the contribution of VEERU / Pan Livestock Services Ltd colleagues: Kulwant Channa for his technical support, and Nick Taylor in the proof-reading.

# Section 1: Description of the study and methods used

## 1.1 Introduction

This is the 14th annual study describing key indicators of production, fertility and health in commercial black and white dairy herds in the United Kingdom. The key performance indicators (KPIs) are based on milk recording data from 500 commercial black and white herds for the 12 month period ending on 31st August 2023. Herd selection used random numbers to ensure a representative cross-section of all herds that milk record with National Milk Records (NMR).

The range in performance across these herds is described for 38 of 80 available parameters clearly showing the wide differences in performance, as well as huge potential for improvement, in commercial dairy herds. This includes six additional parameters (including mastitis rate/100 cows in milk/year) shown since the 2016 study. The principal objective throughout has been to provide farmers and technical advisers with accurate and up-to-date information on the variation in performance of commercial dairy herds.

The calculations used to generate these parameters are identical to those used by the InterHerd+ program allowing farmers and technical advisers to compare the performance of any milk recording herd directly with the 500 herd sample that is representative of the national performance. In other words, for each parameter: **"Is the performance of my herd typical/outlying, good/acceptable/poor when compared to the 500 herds?"**. This prompts discussion around: **"Why is a parameter where it is? Which parameters could/should we prioritise/improve and what are the likely implications?"** If this promotes discussion between farmers and their technical advisers into the different causes and options for improvement then the study has served its primary purpose.

Following the analysis of individual parameters, the trends since 2010 for a selection of the KPIs are displayed, followed by on discussions on their practical use by farmers and advisers. A KPI template of 80 parameters for use in InterHerd+ is also available for users to update the KPI parameters to the target values from the 2023 study.

## 1.2 The sample of herds

The source of data is the monthly milk records obtained by NMR. The 500 herds used in the study all fully milk record on a monthly assisted basis and are approximately 10% of herds recorded by NMR. Herds were selected using random numbers to ensure a representative cross-section of the sample. The herds are all predominantly comprised of black and white breeds (Holstein, Holstein-Friesian, Friesian) and have recorded for a minimum of two years. Where possible the same herds used in the 2022 study were maintained for the 2023 herds' sample. Herds with poorly recorded fertility data (inadequate recording of services and pregnancy diagnoses), as well as herds no longer recording, were replaced with new herds, again selected using random numbers. In total, 451 herds (about 90%) were in both the 2022 and 2023 studies.

Herd size for the 500 herds in the 2023 study ranged from minimum 41 to maximum 1199 cows, with a median value of 178 cows, as shown in Figure i. In the sample 61% of herds were comprised of less than 200 cows, with 41 herds containing over 400 cows.

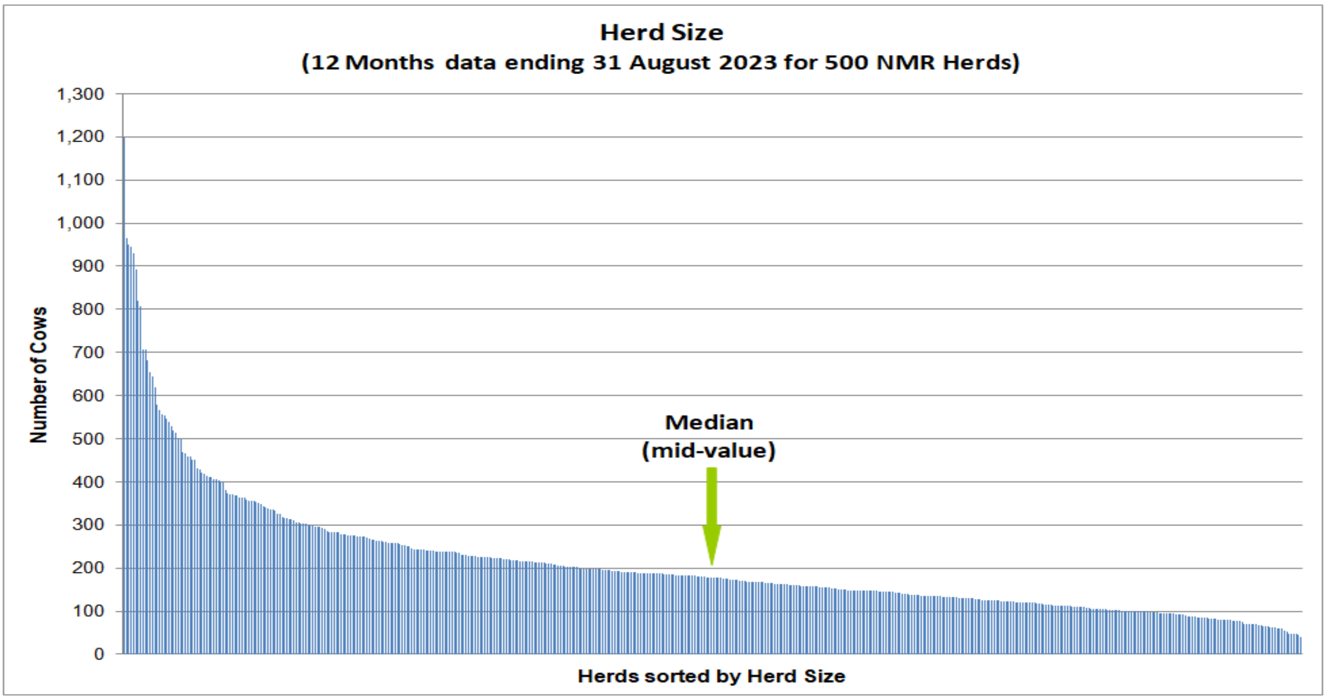


**Figure i Herd size of the 500 herds.**

## 1.3 Description of parameters

For 35 parameters described in this study (out of total of 38), the performance level of each of the 500 herds (242 herds for mastitis rate) is presented as a bar chart. The herds are displayed from "best" to "worst", in ascending or descending order depending on whether it is generally preferable to have a low value (e.g. SCC, calving interval) or a high value (e.g. dry period cure, conception rate). The "best" is always on the left side, nearest the vertical Y axis. For each parameter, a median (middle) value and inter-quartile range values (the level achieved by the middle 50% of herds) are also derived.

The **target** value proposed for each parameter (and included in the KPI template) is the level achieved by the **"best" 25%** of the herds for that parameter. In other words, **the target is set at a level that is currently achieved (or exceeded) by 25% of herds over the last year.**

To minimize the impact of short term seasonal variations, the key performance indicator values are calculated using data recorded over a full 12 month period. They represent the consolidated or average performance levels achieved by each herd for the period from 01 September 2022 to 31 August 2023. The definitions of each parameter are detailed in Appendix 1.

The results of the study are summarized in Table KPIs A and Table KPIs B.
For each parameter, four statistics are presented:

1. The **median**: The middle value. If the performance levels of all herds are arranged in ascending order, the median is the performance of the middle herd (or the average of the middle two herds in an even number of herds). Half the herds do better and half do worse than the median value.
2. The **first quartile (25% value) and third quartile (75% value)**. With the median, these split the herds into four equal groups. The first and third quartile values are the lower and upper limits of performance achieved by the middle 50% of herds. 25% achieve "better" and 25% achieve "worse" than the limits for that parameter.
3. The **target** value used by InterHerd+ is the level achieved or bettered by 25% of the herds in the study. This value is the "**better**" of the **first quartile (25%) or third quartile (75%)**

**values.** For parameters like somatic cell count, culling % and calving interval the target will be the 25% (lower) value, while for others (conception %, protein %, dry period cure %) it will be the 75% (higher) value.

4. The **inter-quartile range** is the difference between the performance of the best and worst 25% of herds (i.e. the difference between the **first quartile (25% value) and third quartile (75% value)**. This gives an impression of how widely herds in the middle 50% differ.

The position of these values is graphically displayed in Figure ii.

Throughout this report the parameter value is displayed on the vertical Y axis and bars representing the study herds are arranged along the horizontal X axis. The "best" performing herd is nearest the vertical Y axis with the worst performing herd furthest away.

The parameter described in Figure ii is the herd average SCC so the target value is at the lower end of the inter-quartile range (as a low average SCC is preferable to a high average SCC).

.

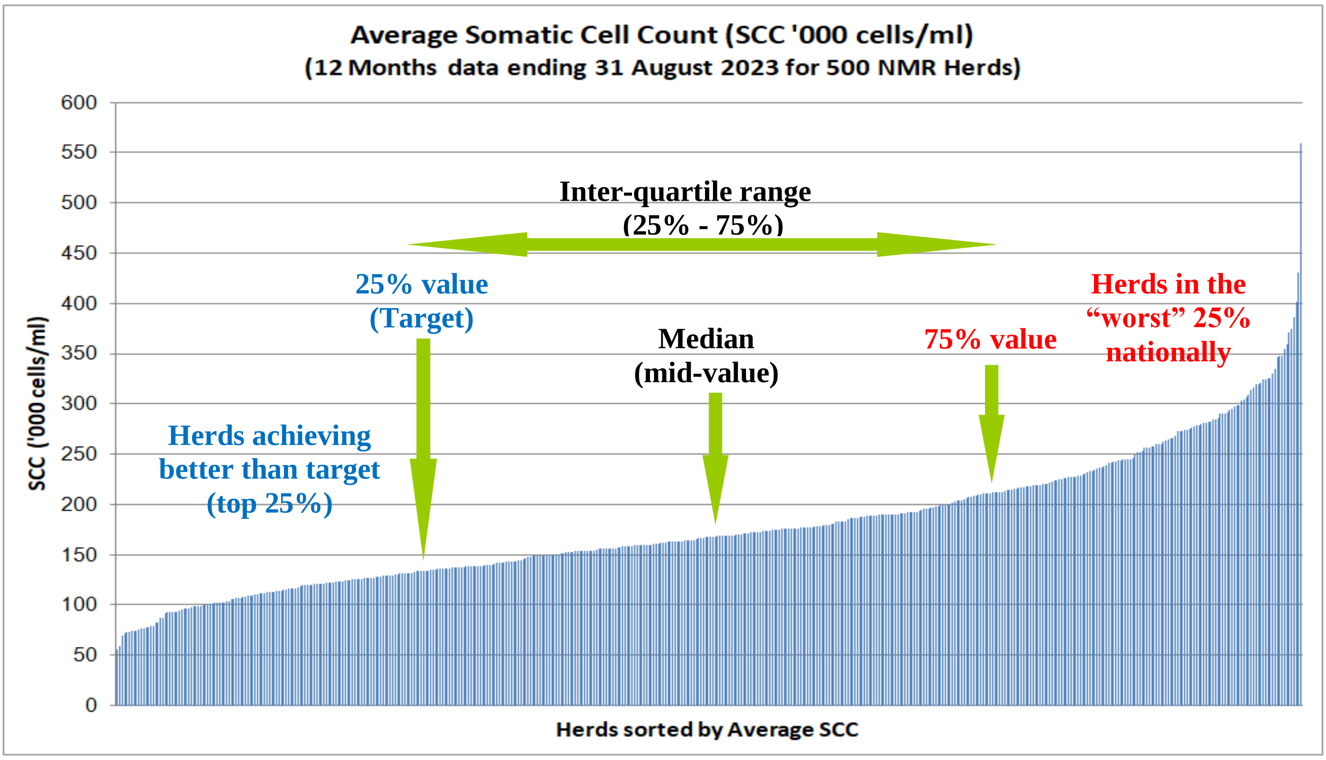


**Figure ii A description of the median, inter-quartile range and target values generated for each parameter.**

## 1.4 Changes to studies from earlier years

1. **Mastitis KPIs:** The results of the study summarized in Table KPIs A and Table KPIs B. include 4 parameters relating to mastitis, including the herd overall mastitis rate (cases/100 cows in milk/year). This is the eighth year that mastitis related parameters have been derived from the survey herds, reflecting the significant improvement in the level of reporting of mastitis by farmers in recent years. While in 2012 less than 20% of herds reported any mastitis, this has risen to 48% of herds in 2023. For the purpose of this study any herd that recorded over 5 cases/100 cows in milk/ year was considered to record mastitis. The mastitis KPIs in this report are calculated based on 242 of the 500 herds (about 48%) that qualified this year.

2. **305 day milk/protein/fat yields:** Until 2015, 305 day yield parameters were calculated from all lactations, including lactations that were shorter than 305 days. In this report, as in all years since 2016, the lactation must be at least 305 days long for inclusion in the calculation. This report also details the 305 day yields (total yield up to and including the 305$^{th}$ day of lactation) of fat and protein. As with the milk yield, these parameters are calculated from lactations that were at least 305 days in length. Note, the exclusion of lactations shorter than 305 days does not apply to the calculation of the **overall lactation yield**. In some herds, when many cows have lactation lengths below 305 days, this can result in lactation yields smaller than the 305 day yields.

# Section 2: KPI results for the year ending 31 August 2023

**Table KPIs A. Summary of culling, fertility and milk related KPIs derived from the analysis of 500 NMR milk recording herds for the year ending 31 August 2023.**

| Parameter | Median (1) | 1st – 3rd quartile (25% - 75%) (2) | Target (3) | IQR (4) |
|---|---|---|---|---|
| A. Culling rate | 28% | 22% - 34% | **22%** | 12% |
| B. Percentage culled (off take) 100 days after calving | 5% | 3% - 8% | **3%** | 5% |
| C(a). Age at exit (years) | 6.0 | 5.4 – 6.7 | **6.7** | 1.3 |
| C(b). Age at exit (days) | 2,179 | 1,964 – 2,433 | **2,433** | 469 |
| D. Age at exit by lactations | 3.6 | 3.2 – 4.2 | **4.2** | 1.0 |
| E. Percentage Served by day 80 | 59% | 44% - 69% | **69%** | 25% |
| F. Percentage conceived 100 days after calving | 39% | 26% - 46% | **46%** | 20% |
| G. Calving to 1st service interval (days) | 81 | 71 – 100 | **71** | 29 |
| H. Calving interval (days) | 394 | 383 – 410 | **383** | 27 |
| I(a). Age at 1st calving (years) | 2.2 | 2.1 – 2.4 | **2.1** | 0.3 |
| I(b). Age at 1st calving (days) | 804 | 761 – 871 | **761** | 110 |
| J. Conception rate | 39% | 32% - 45% | **45%** | 13% |
| K. %Service intervals at 18-24 days (Heat detection) | 41% | 32% - 48% | **48%** | 16% |
| L. Percentage service intervals >50 days | 19% | 12% - 29% | **12%** | 17% |
| M. %Cows eligible for service served (Submission rate) | 41% | 27% - 53% | **53%** | 26% |
| N. %Eligible for service that conceived (Pregnancy rate) | 16% | 11% - 21% | **21%** | 10% |
| O. Lifetime milk / cow / day (kg) | 12.7 | 10.5 – 14.6 | **14.6** | 4.1 |
| P. Milk / cow / year (kg) | 8,737 | 7,510 – 9,961 | **9,961** | 2,451 |
| Q. Average protein% | 3.36% | 3.29% - 3.43% | **3.43%** | 0.14% |
| R. Average fat% | 4.26% | 4.08% - 4.45% | **4.45%** | 0.37% |
| S. 305-day milk yield (kg) | 8,991 | 7,810 – 10,154 | **10,154** | 2,344 |
| T. 305-day protein yield (kg) | 298 | 259 – 335 | **335** | 76 |
| U. 305-day fat yield (kg) | 378 | 329 – 424 | **424** | 95 |

(1) The median is the middle value (so 250 herds were better and 250 were worse than this value).
(2) The **first quartile (25% value) and third quartile (75% value)** describe the lower and upper limits of performance achieved by the middle 50% of herds. 25%, or one in four, herds achieve "better" and 25% "worse" than the limits for that parameter.
(3) The **target** is set at the level achieved or bettered by the **"best" 25% of herds**. **One in four of the 500 herds in the sample achieved this level or better**.
(4) The inter-quartile range (IQR) is the difference between the **first quartile (25% value) and third quartile (75% value)**. This gives an impression of how widely herds in the middle 50% differ.

**Table KPIs B. Summary of the somatic cell count (SCC) and mastitis related KPIs derived from analysis of 500 NMR milk recording herds for the year ending 31 August 2023.**

| Parameter | Median (1) | 1st - 3rd quartile (25% - 75%) (2) | Target (3) | IQR (4) |
|---|---|---|---|---|
| V. Herd SCC ('000 cells/ml) | 168 | 133 - 214 | **133** | 81 |
| W. % milk samples with **High** SCC (*) | 15% | 12% - 20% | **12%** | 8% |
| X. % milk samples with SCC ≥500,000 cells/ml | 7% | 5% - 9% | **5%** | 4% |
| Y. % cows with **High** SCC at 1st record in lactation (*) | 15% | 12% - 20% | **12%** | 8% |
| Z. % **Chronic** milk samples (**) | 8% | 6% - 11% | **6%** | 5% |
| ZA. Dry period cure (High:Low) (***) | 77% | 69% - 86% | **86%** | 17% |
| ZB. Dry period protection (Low:Low) (***) | 86% | 80% - 90% | **90%** | 10% |
| ZC. % Low at last recording of previous lactation (*) | 79% | 70% - 84% | **84%** | 14% |
| ZD. % samples New SCC category (**) | 5% | 4% - 7% | **4%** | 3% |
| ZE. % cows dried-off with no High SCC samples in the lactation (*) | 51% | 42% - 59% | **59%** | 17% |
| ZF. Threshold Index new high / new low (****) | 1.20 | 1.07 - 1.32 | **1.07** | 0.25 |
| ZG. % of cows with New/First/Repeat sample that are Low SCC at next recording (**) | 57% | 52% - 62% | **62%** | 10% |
| ZH. % of cows with Chronic sample that are low SCC at next recording (**) | 21% | 17% - 26% | **26%** | 9% |
| ZI. Percentage drying off with no mastitis cases+ | 85% | 79% - 91% | **91%** | 12% |
| ZJ. Mastitis rate (cases/100 cows in milk/year)+ | 22 | 13 - 36 | **13** | 23 |
| ZK. Cows with Index mastitis case by Day 30+ | 3% | 2% - 6% | **2%** | 4% |
| ZL. Index mastitis rate after Day 30+ | 15% | 9% - 25% | **9%** | 16% |

(*) **HIGH** SCC is a milk sample with ≥200,000 cells/ml milk;
**LOW** SCC is a milk sample with below 200,000 cells/ml milk

(**) **CHRONIC** / **NEW** / **FIRST** and **REPEAT** are the Herd Companion categories describing high SCC cows. See Appendix 2 for definitions.

(***) **Dry period protection (High:Low):** The percentage of cows finishing a lactation with a HIGH SCC sample that starts the new lactation with a LOW SCC sample;
**Dry period cure (Low:Low):** The percentage of cows finishing a lactation with a LOW SCC sample that starts the new lactation with a LOW SCC sample.

(****) **Threshold index:** The total cows changing from Low to High SCC divided by the total cows changing from High to Low SCC at consecutive milk recordings.

(1) The median is the middle value (so 250 herds were better and 250 were worse than this value).

(2) The **first quartile (25% value) and third quartile (75% value)** describe the lower and upper limits of performance achieved by the middle 50% of herds. 25%, or one in four, herds achieve "better" and 25% "worse" than the limits for that parameter.

(3) The Target is set at the level achieved or bettered by the **"best" 25% of herds**. **One in four of the 500 herds in the sample achieved this level or better**.

(4) The inter-quartile range is the difference between the **first quartile (25% value) and third quartile (75% value)**. This gives an impression of how widely herds in the middle 50% differ.

\+ **The mastitis parameters are derived from a group of 242 herds (within the 500 herds in the study) where mastitis rate >5 cases per 100 cows in milk / year.**

**A. Culling/death rate: The % of cows that left the herd (culled/sold/died) in the last 12 months.**

| | |
|---|---|
| Target (top ¼ of herds' level): | 22% |
| Median: | 28% |
| 75% level: | 34% |
| Inter-quartile range: | 12% |

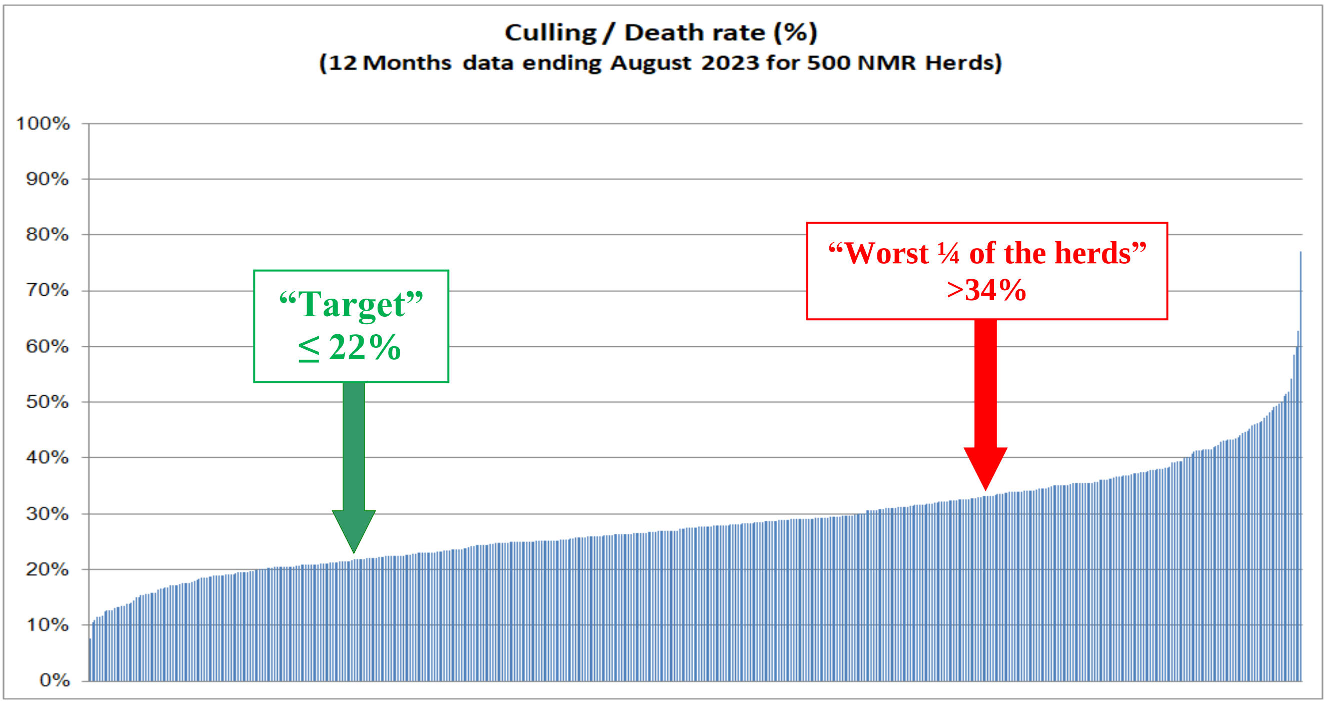


**B. Percentage of cows/heifers calving during the last 12 months that were culled (off take) / died during the first 100 days after calving. A possible indicator of "involuntary culling".**

| | |
|---|---|
| Target (top ¼ of herds' level): | 3% |
| Median: | 5% |
| 75% level: | 8% |
| Inter-quartile range: | 5% |

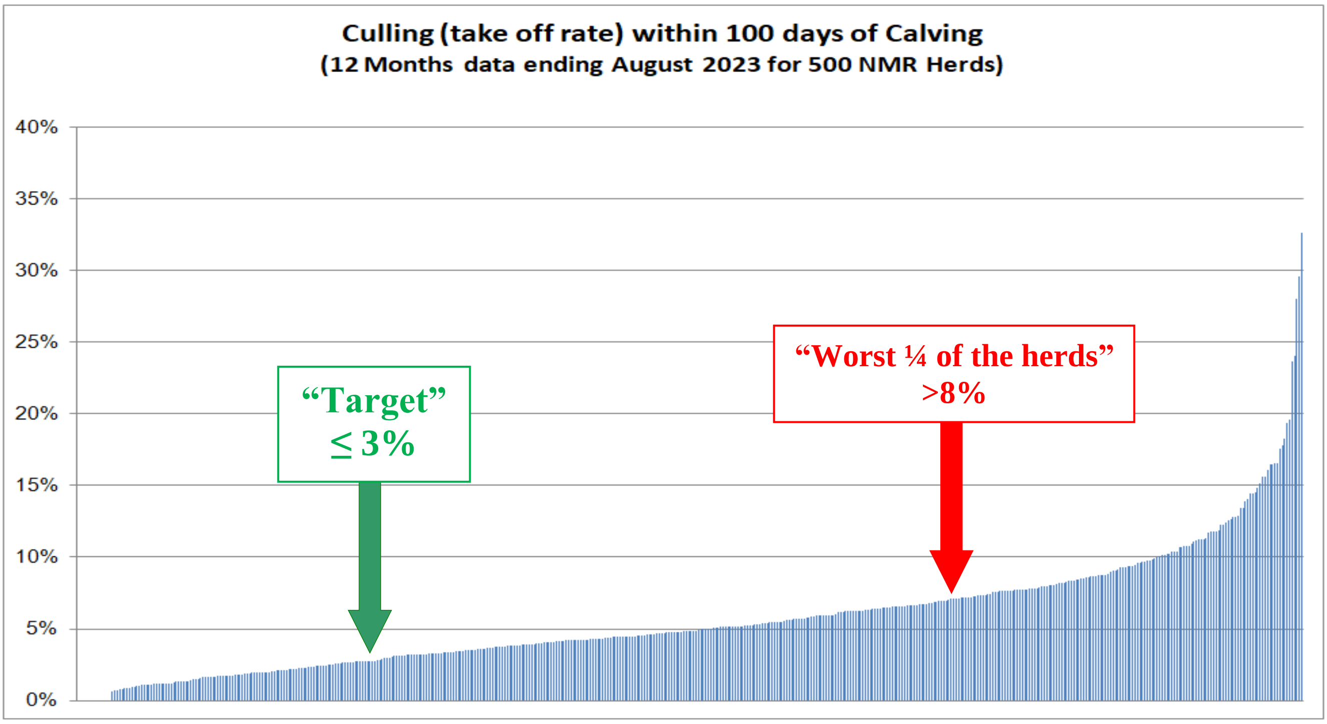

**C. Average age (in years) at exit: The average age of cows leaving the herd in the last 12 months at the time of exit. A potential measure of longevity.**

| | |
|---|---|
| **Target (top ¼ of herds' level):** | **6.7 (2433 days)** |
| Median: | 6.0 (2179 days) |
| 75% level: | 5.4 (1964 days) |
| Inter-quartile range: | 1.3 (469 days) |

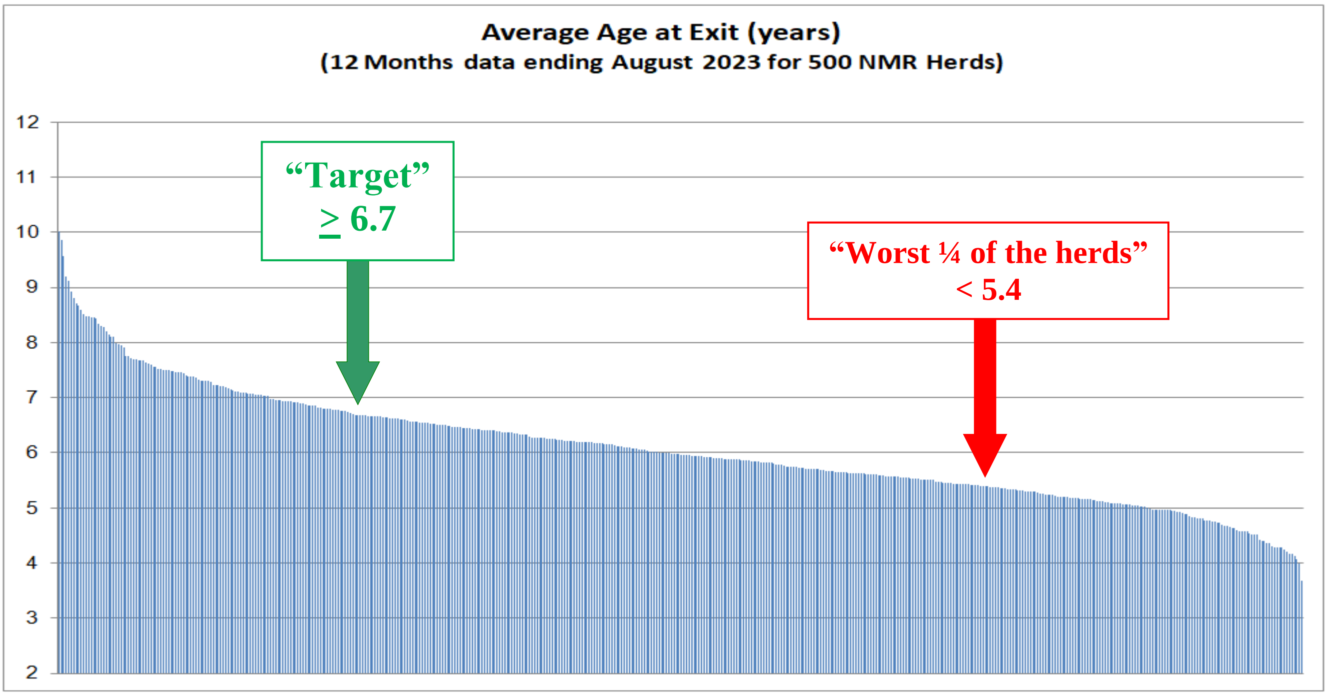


**D. Average Age at exit by lactations: The average lactation number of cows leaving the herd in the last 12 months. A potential measure of longevity.**

| | |
|---|---|
| **Target (top ¼ of herds' level):** | **4.2** |
| Median: | 3.6 |
| 75% level: | 3.2 |
| Inter-quartile range: | 1.0 |

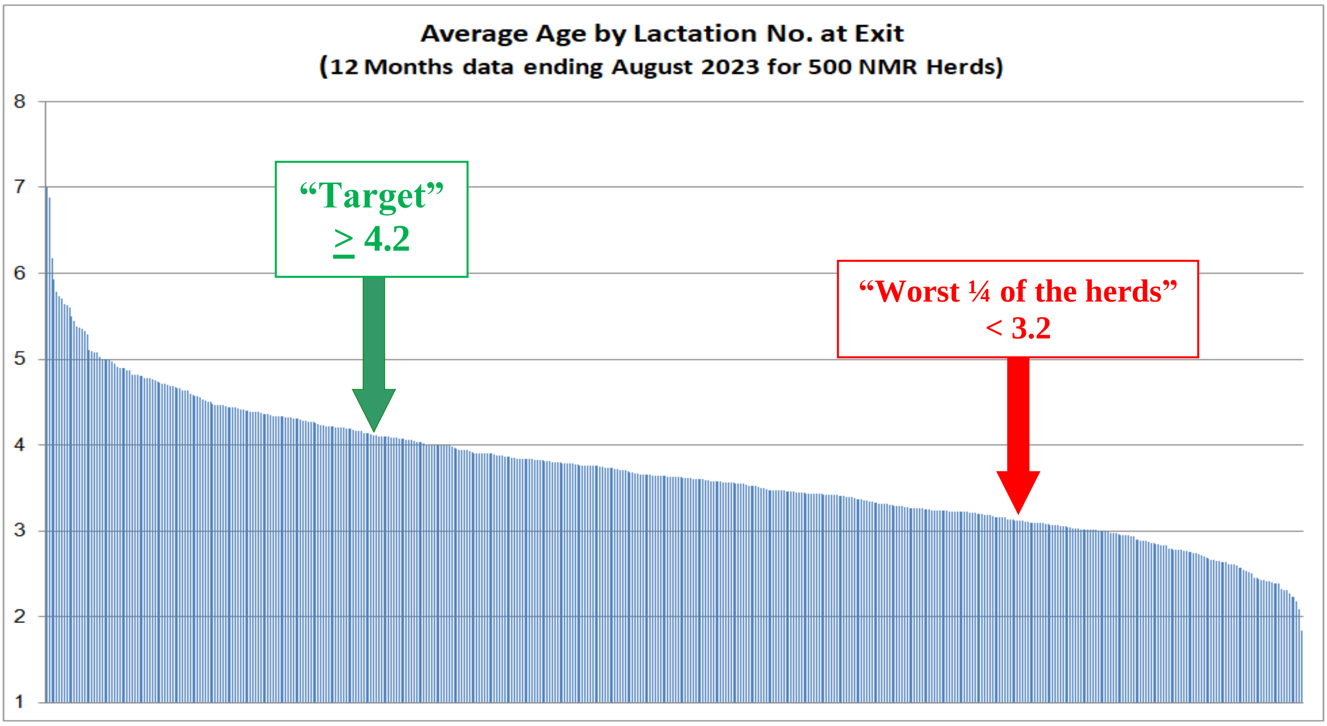

**E. % Served by day 80: The percentage of calving cows served at least once within 80 days of calving.**

| | |
|---|---|
| **Target (top ¼ of herds' level):** | **69%** |
| Median: | 59% |
| 75% level: | 44% |
| Inter-quartile range: | 25% |

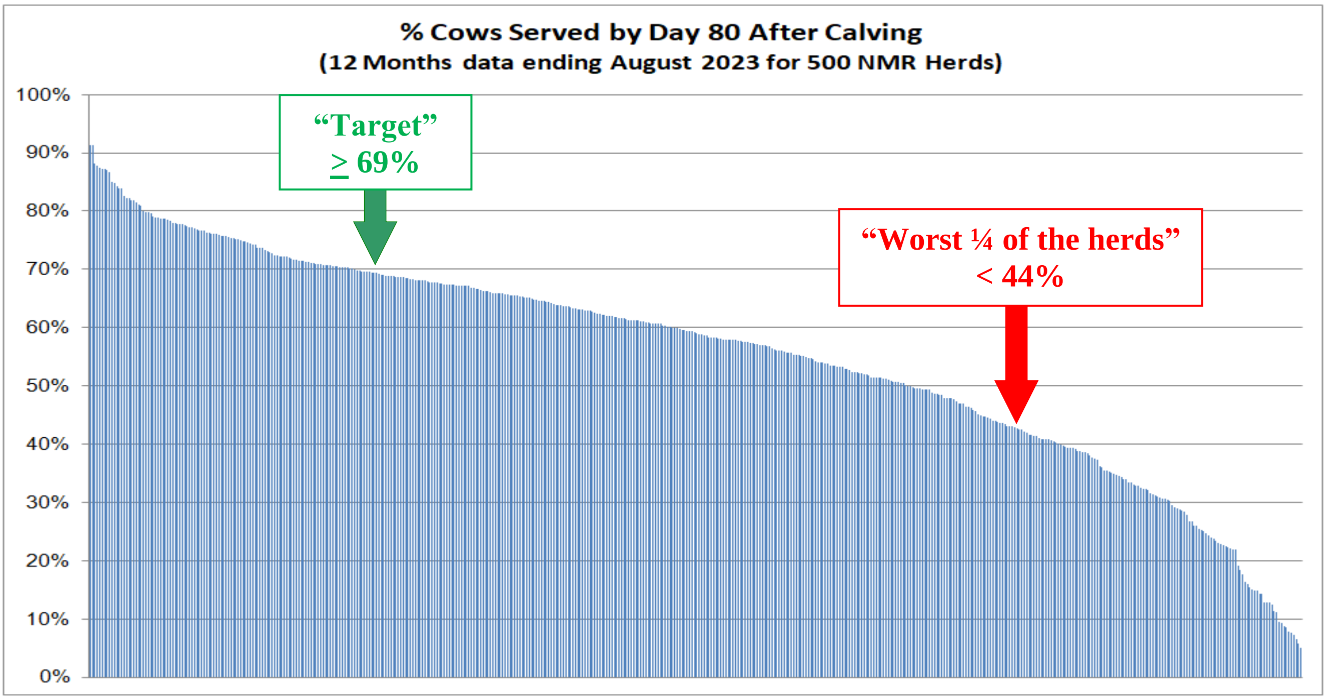


**F. % Conceived 100 days after calving: The percentage of calving cows that had conceived within 100 days of calving.**

| | |
|---|---|
| **Target (top ¼ of herds' level):** | **46%** |
| Median: | 39% |
| 75% level: | 26% |
| Inter-quartile range: | 20% |

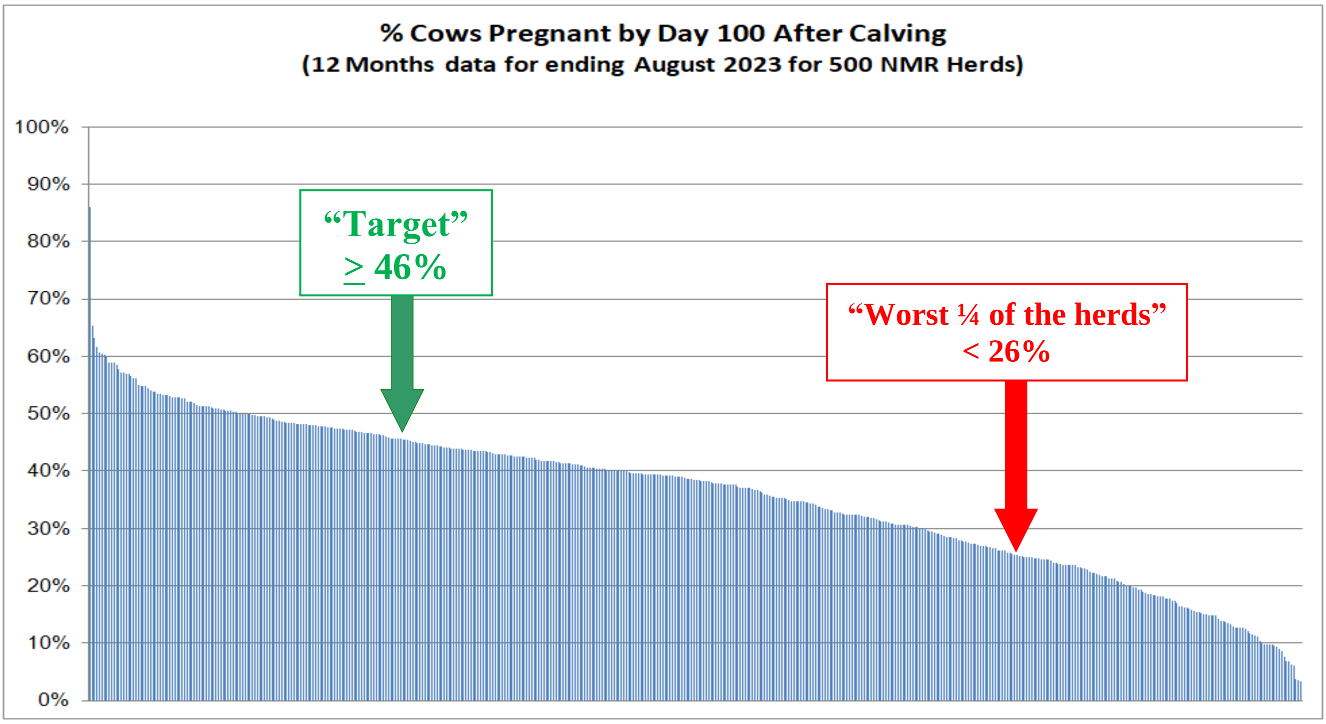

**G. Calving to 1st service interval: The average number of days between calving and 1st service.**

| | |
|---|---|
| Target (top ¼ of herds' level): | 71 |
| Median: | 81 |
| 75% level: | 100 |
| Inter-quartile range: | 29 |

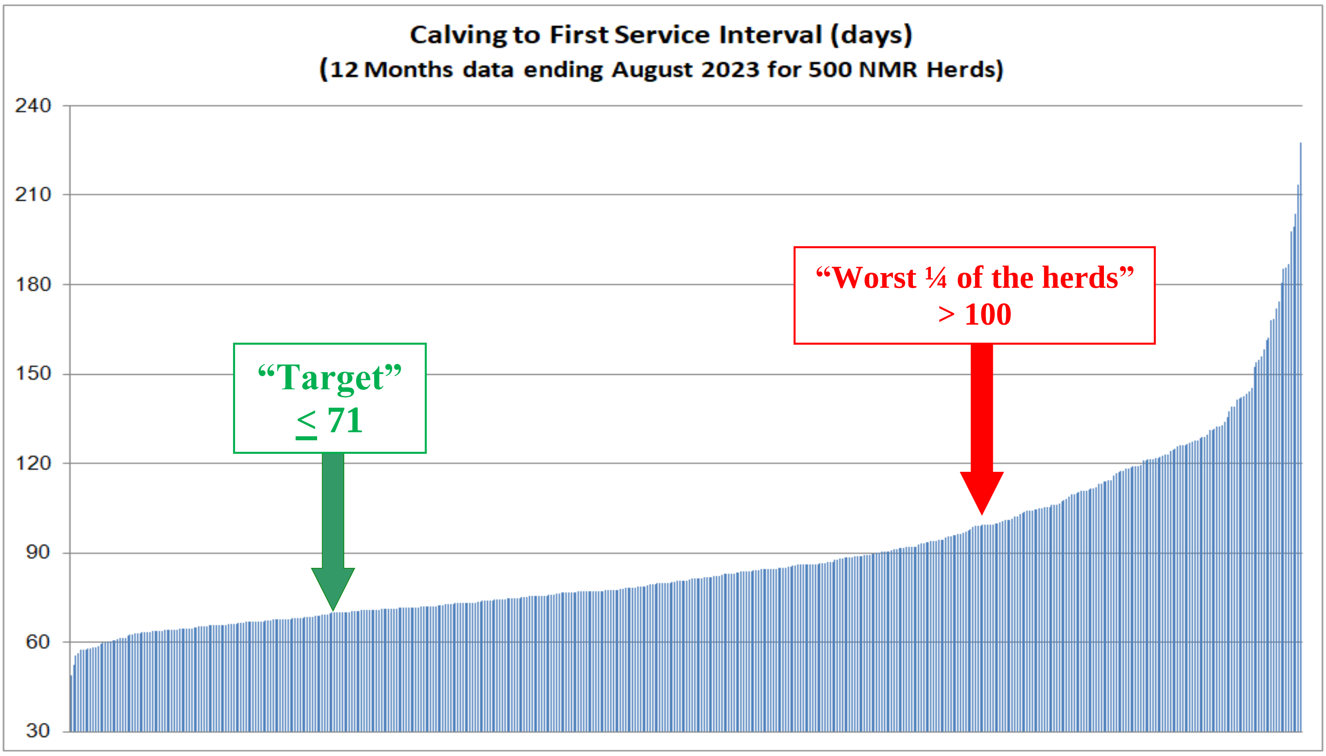


**H. Calving interval: The average interval between consecutive calvings (in days).**

| | |
|---|---|
| Target (top ¼ of herds' level): | 383 |
| Median: | 394 |
| 75% level: | 410 |
| Inter-quartile range: | 27 |

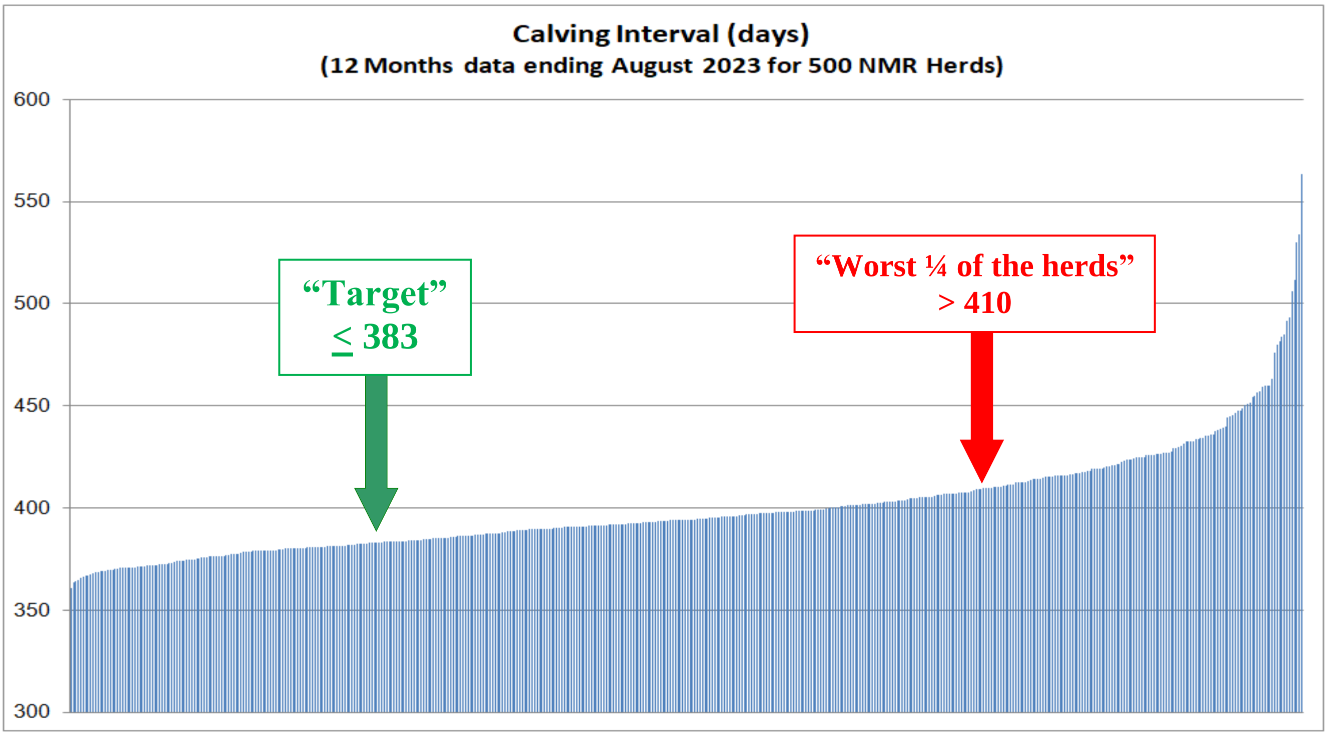

**I. Age at 1st calving: The average age (in years) of heifers calving down over the last year.**

| | |
|---|---|
| Target (top ¼ of herds' level): | 2.1 (761 days) |
| Median: | 2.2 (804 days) |
| 75% level: | 2.4 (871 days) |
| Inter-quartile range: | 0.3 (110 days) |

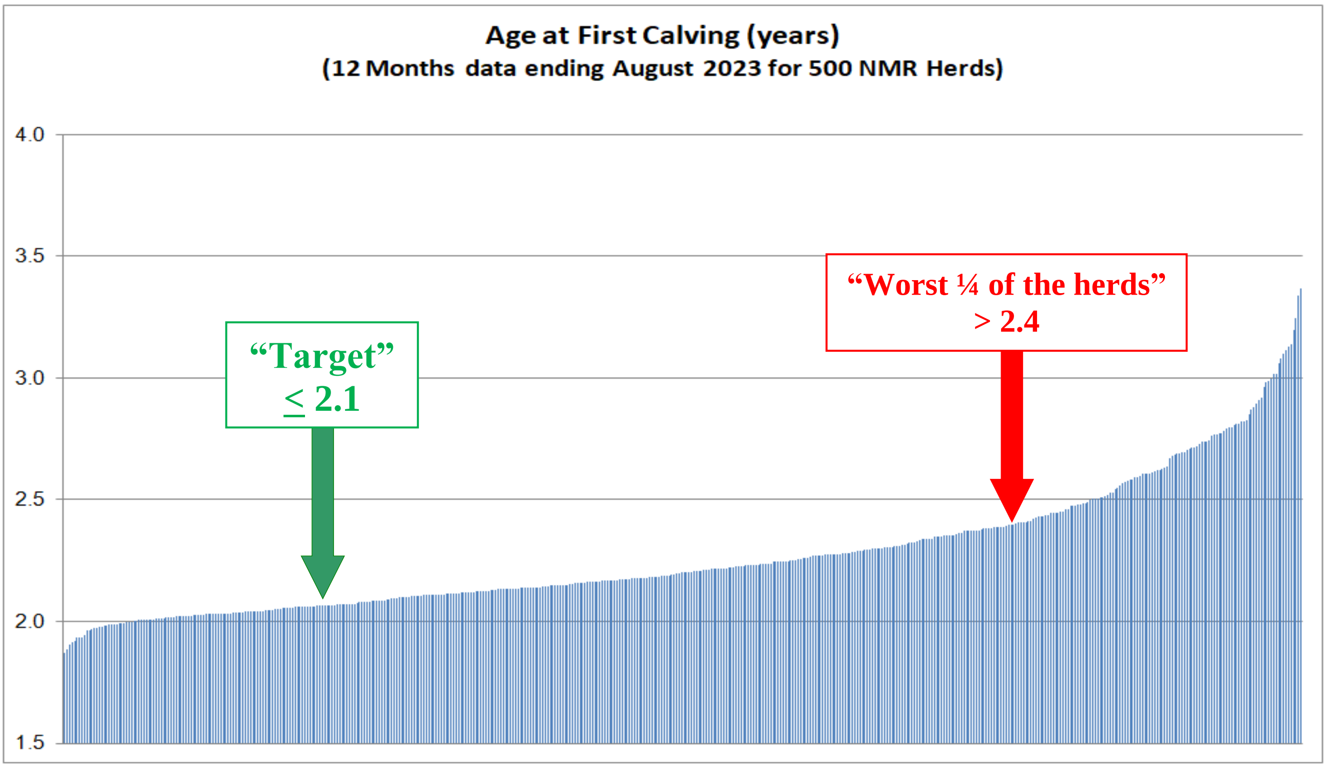


**J. Conception rate: The average conception rate for all services in the last 12 months.**

| | |
|---|---|
| Target (top ¼ of herds' level): | 45% |
| Median: | 39% |
| 75% level: | 32% |
| Inter-quartile range: | 13% |

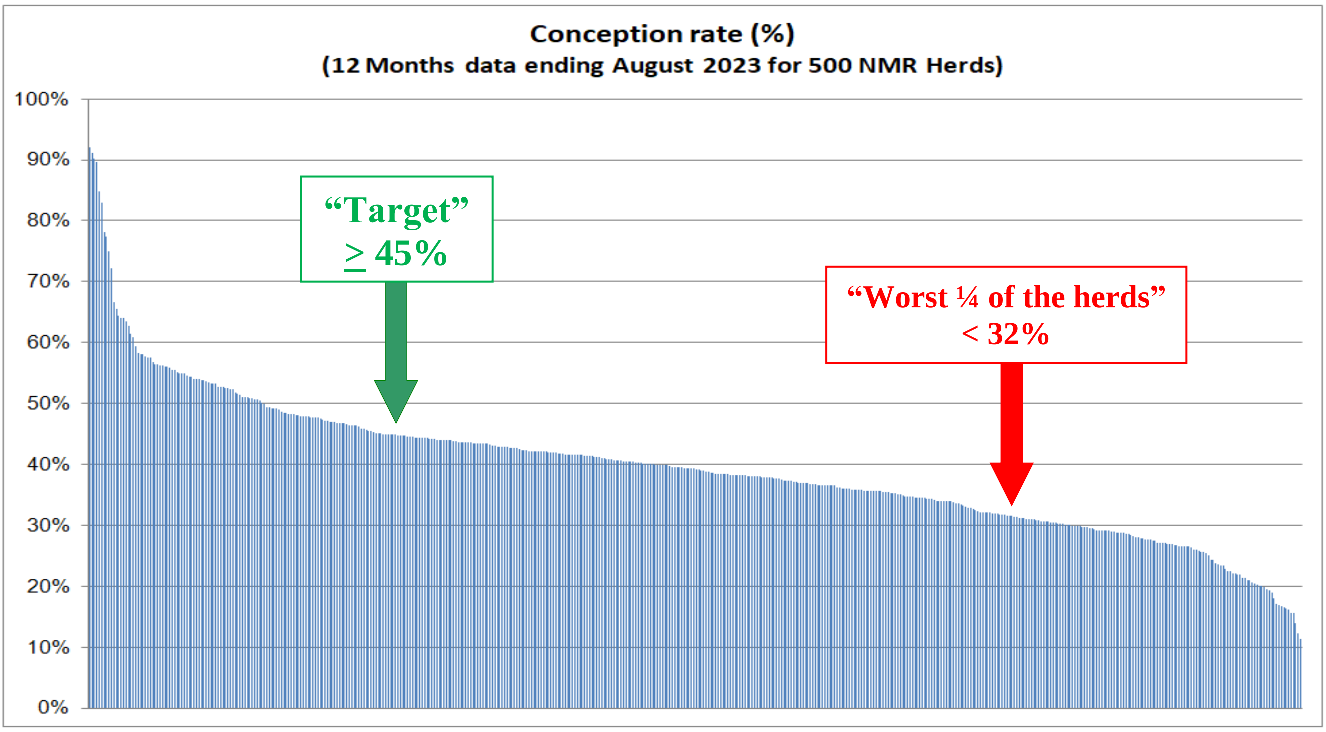

**K. % service intervals at 18-24 days (Heat detection): The percentage of all repeat services occurring 18-24 days (one oestrous cycle) after the previous service.**

| | |
|---|---|
| **Target (top ¼ of herds' level):** | **48%** |
| Median: | 41% |
| 75% level: | 32% |
| Inter-quartile range: | 16% |

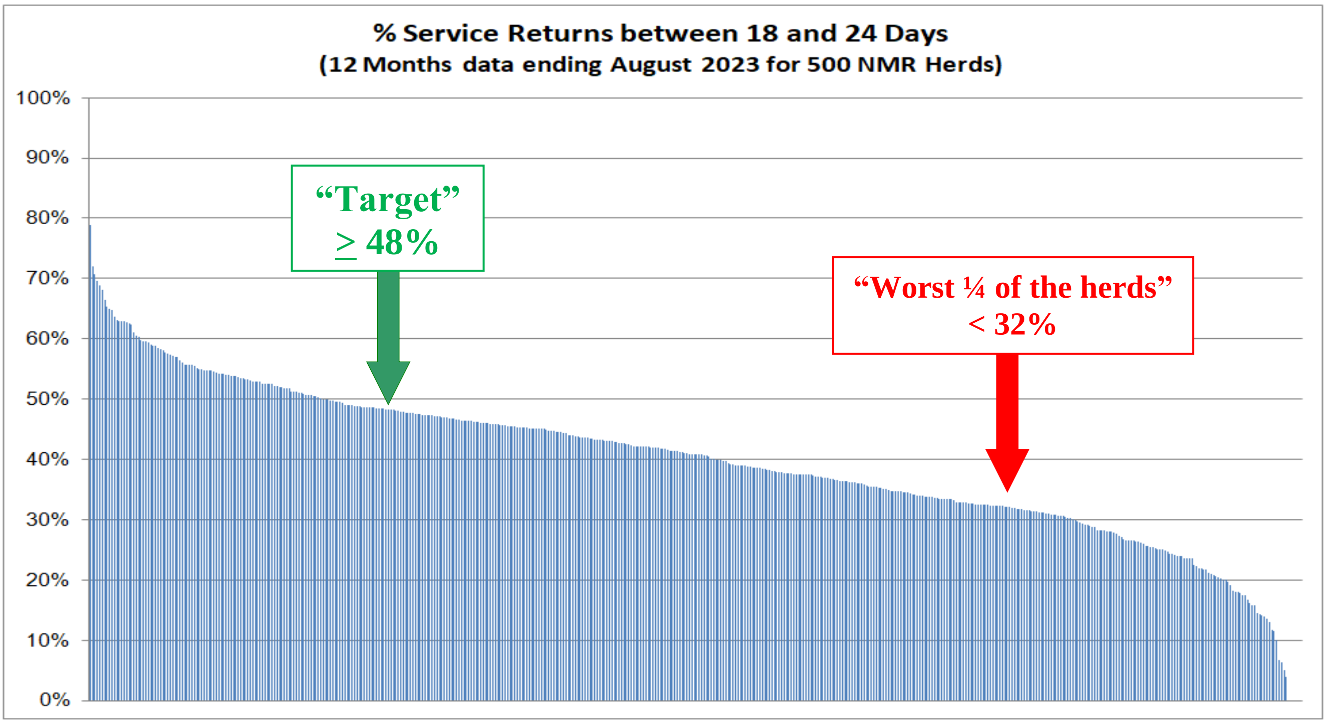


**L. % service intervals >50 days: The percentage of all repeat services with an interval of over 50 days since the previous service. A potential indicator of poor heat detection.**

| | |
|---|---|
| **Target (top ¼ of herds' level):** | **12%** |
| Median: | 19% |
| 75% level: | 29% |
| Inter-quartile range: | 17% |

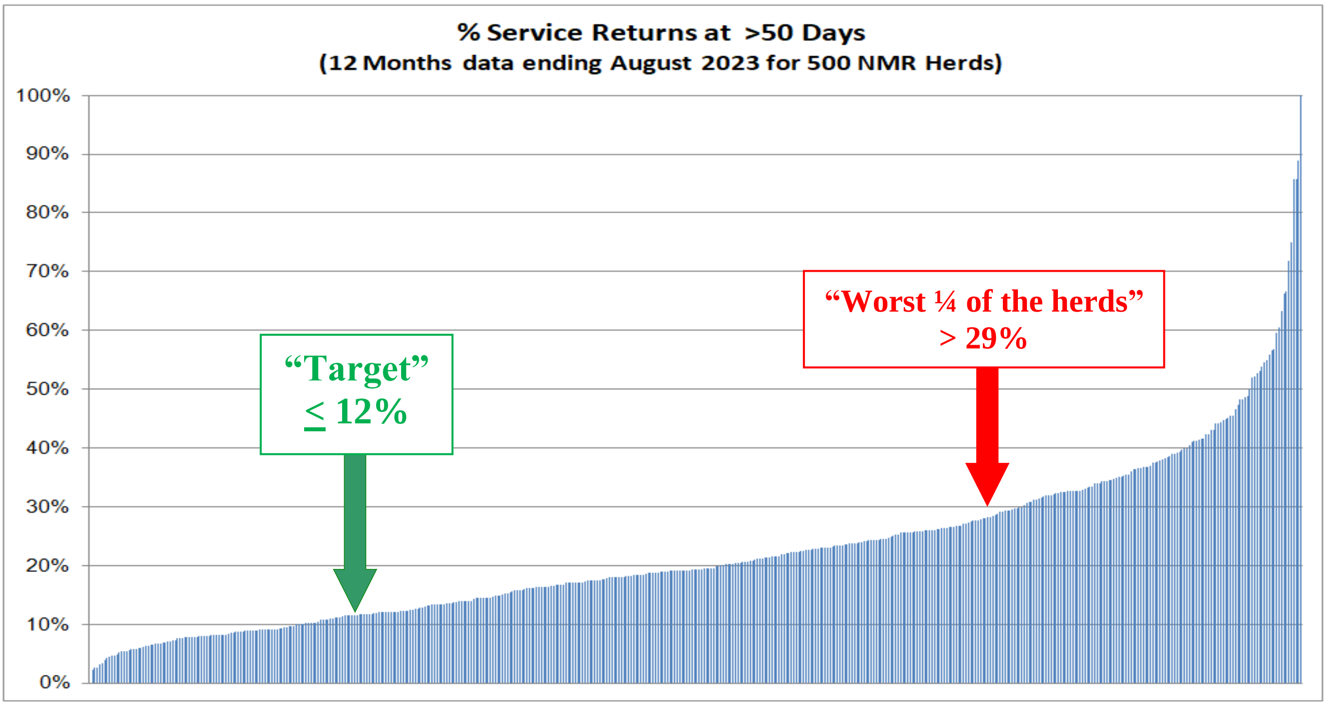

**M. Percentage of cows eligible for service (>42 days calved, not barren, not pregnant) that were served per 21 day oestrous period (Submission rate).**

| | |
|---|---|
| **Target (top ¼ of herds' level):** | **53%** |
| Median: | 41% |
| 75% level: | 27% |
| Inter-quartile range: | 26% |

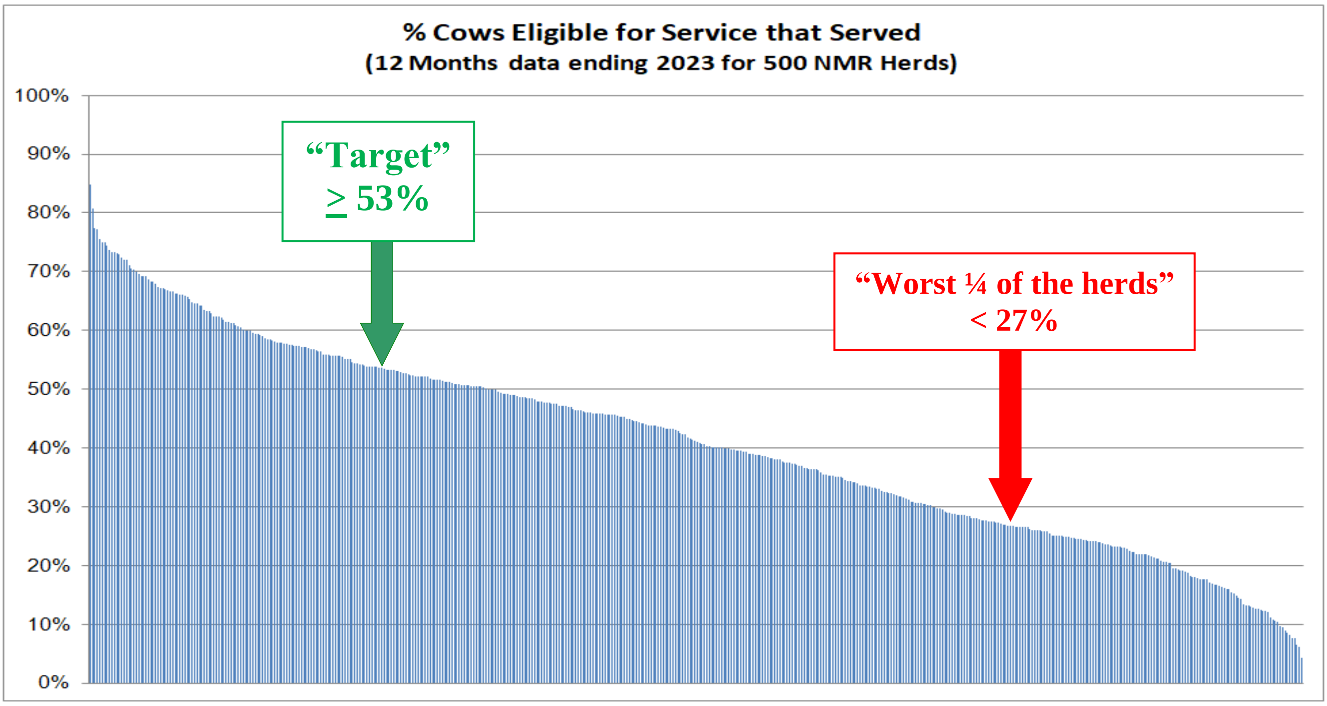


**N. Percentage eligible for service (>42 days calved, not barren, not pregnant) that conceived per 21 day oestrus period (Pregnancy rate).**

| | |
|---|---|
| **Target (top ¼ of herds' level):** | **21%** |
| Median: | 16% |
| 75% level: | 11% |
| Inter-quartile range: | 10% |

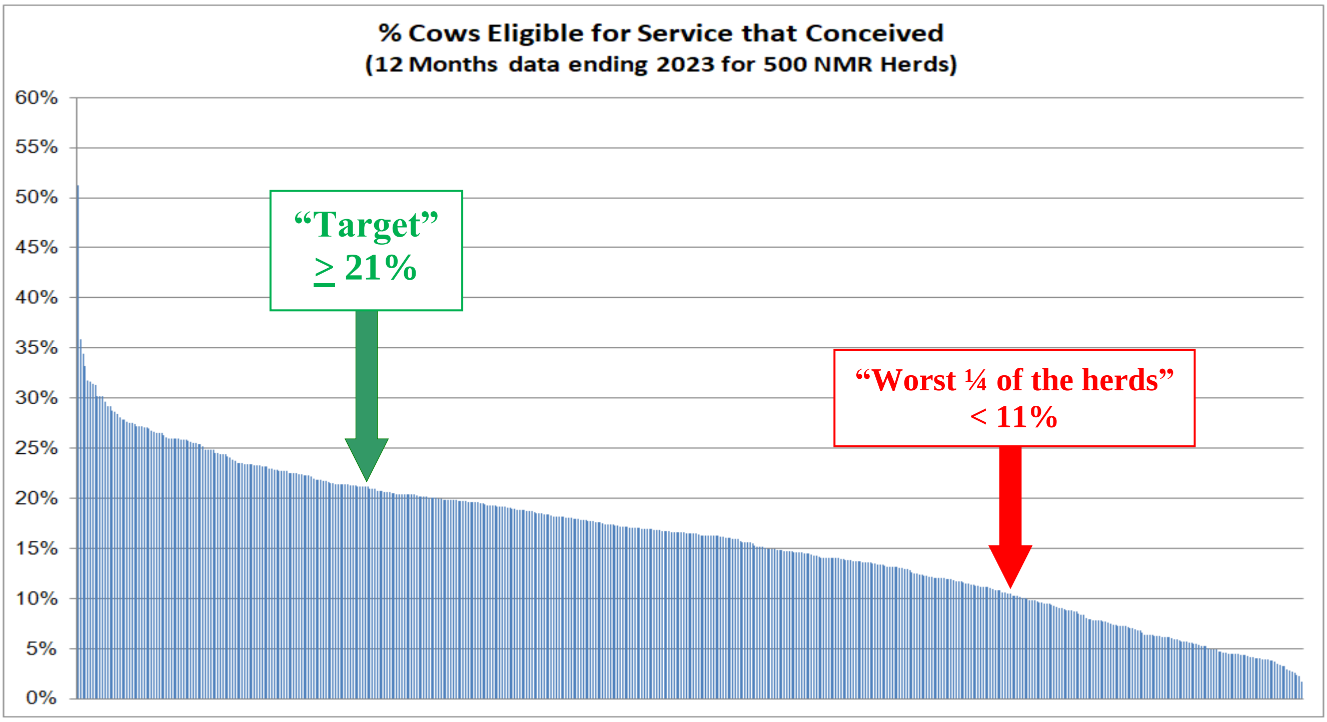

**O. Lifetime milk / cow / day (kg): Equates to the average daily milk yield of cows across their whole lifetime (including unproductive periods: time as a heifer, dry periods).**

| | |
|---|---|
| **Target (top ¼ of herds' level):** | **14.6** |
| Median: | 12.7 |
| 75% level: | 10.5 |
| Inter-quartile range: | 4.1 |

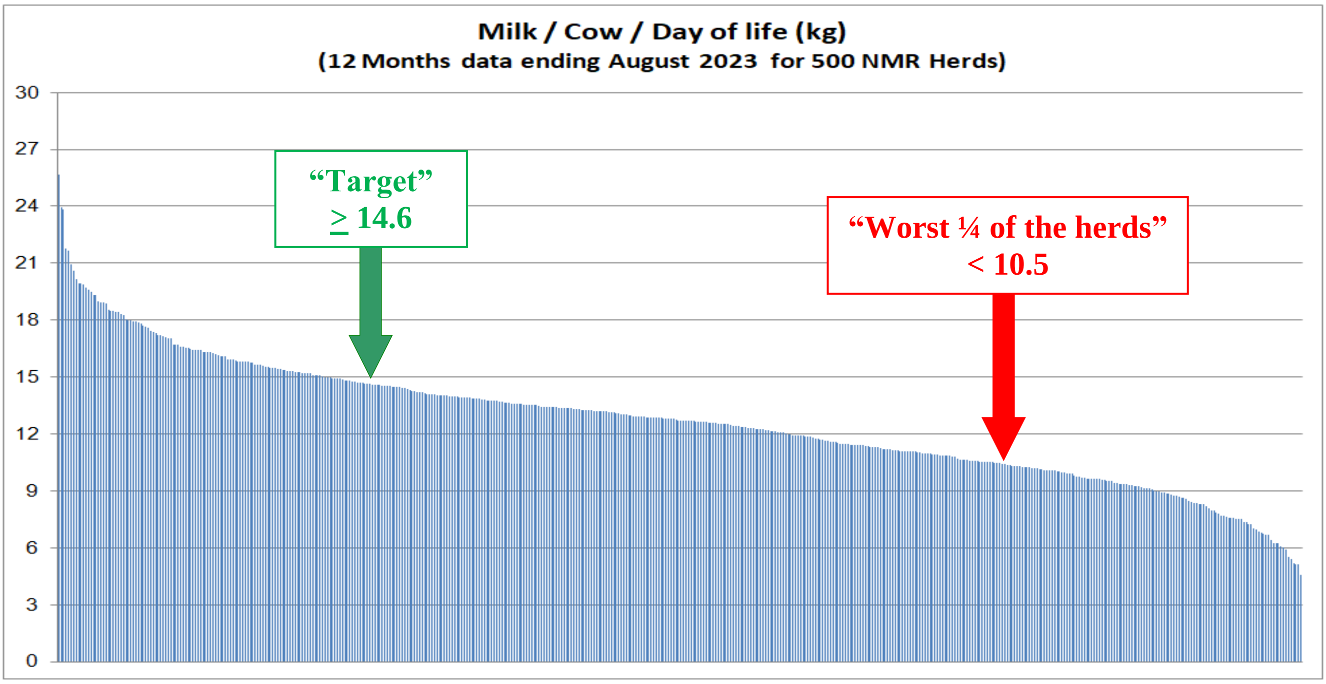


**P. Milk / cow / year (kg): The average annual milk yield of all cows in the year. Total milk divided by the average cow population. A measure of milk yield per cow place in the herd.**

| | |
|---|---|
| **Target (top ¼ of herds' level):** | **9,961** |
| Median: | 8,737 |
| 75% level: | 7,510 |
| Inter-quartile range: | 2,451 |

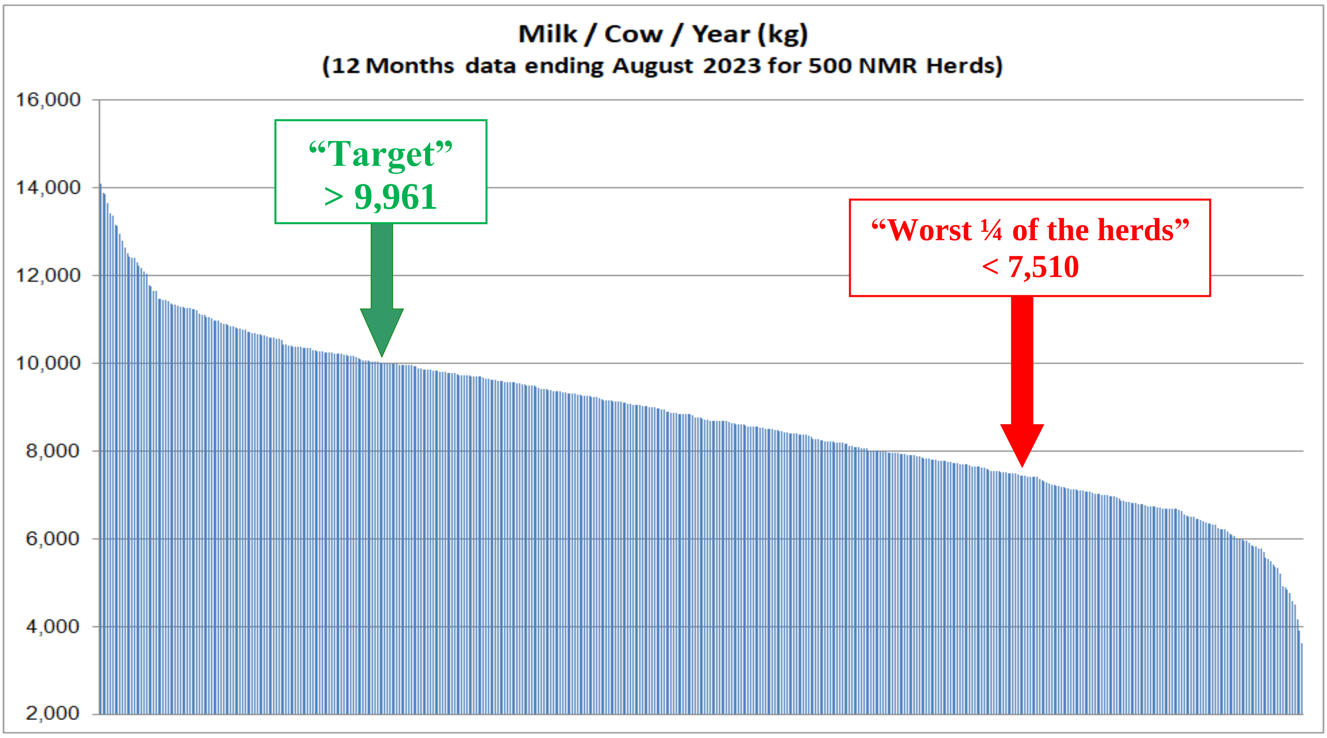

**Q. Average protein (%): The average % protein of all milk samples taken over the year.**

| | |
|---|---|
| Target (top ¼ of herds' level): | 3.43% |
| Median: | 3.36% |
| 75% level: | 3.29% |
| Inter-quartile range: | 0.14% |

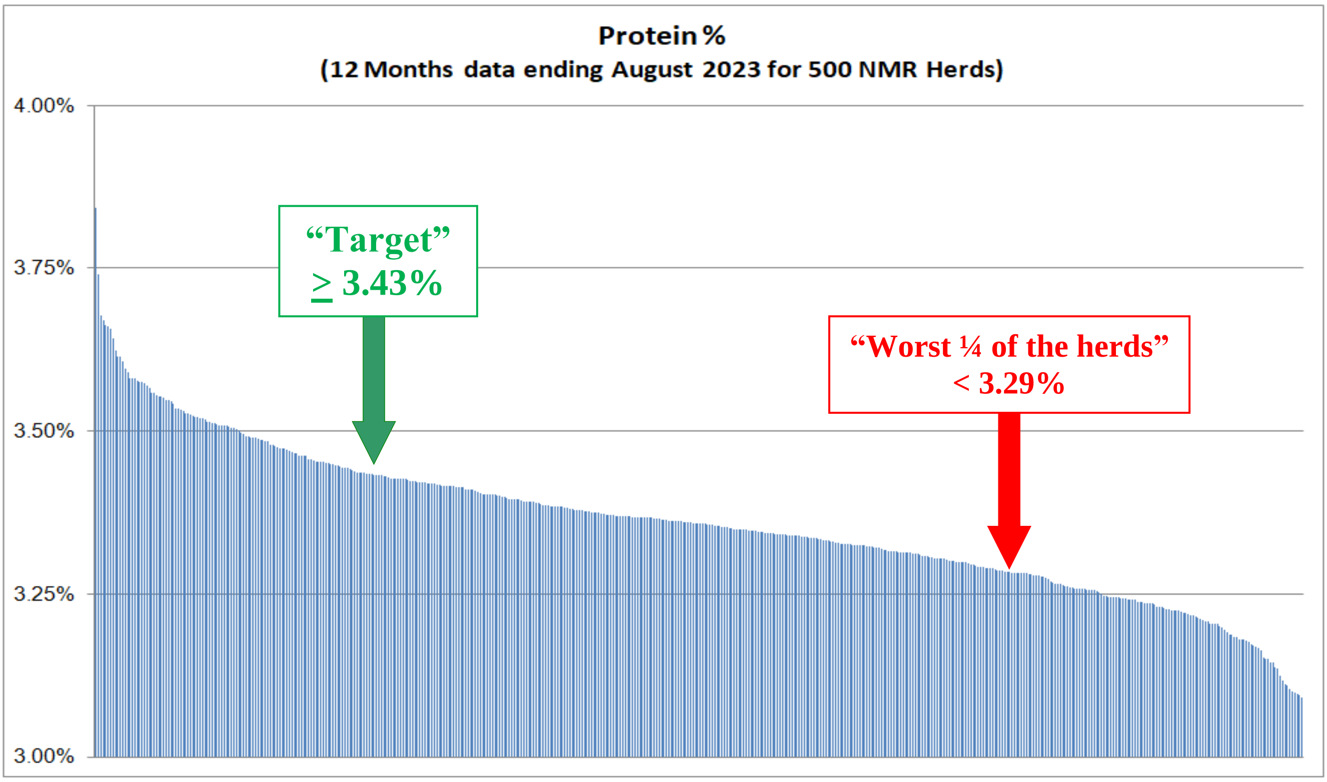


**R. Average fat (%): The average % fat of all milk samples taken over the year.**

| | |
|---|---|
| Target (top ¼ of herds' level): | 4.45% |
| Median: | 4.26% |
| 75% level: | 4.08% |
| Inter-quartile range: | 0.37% |

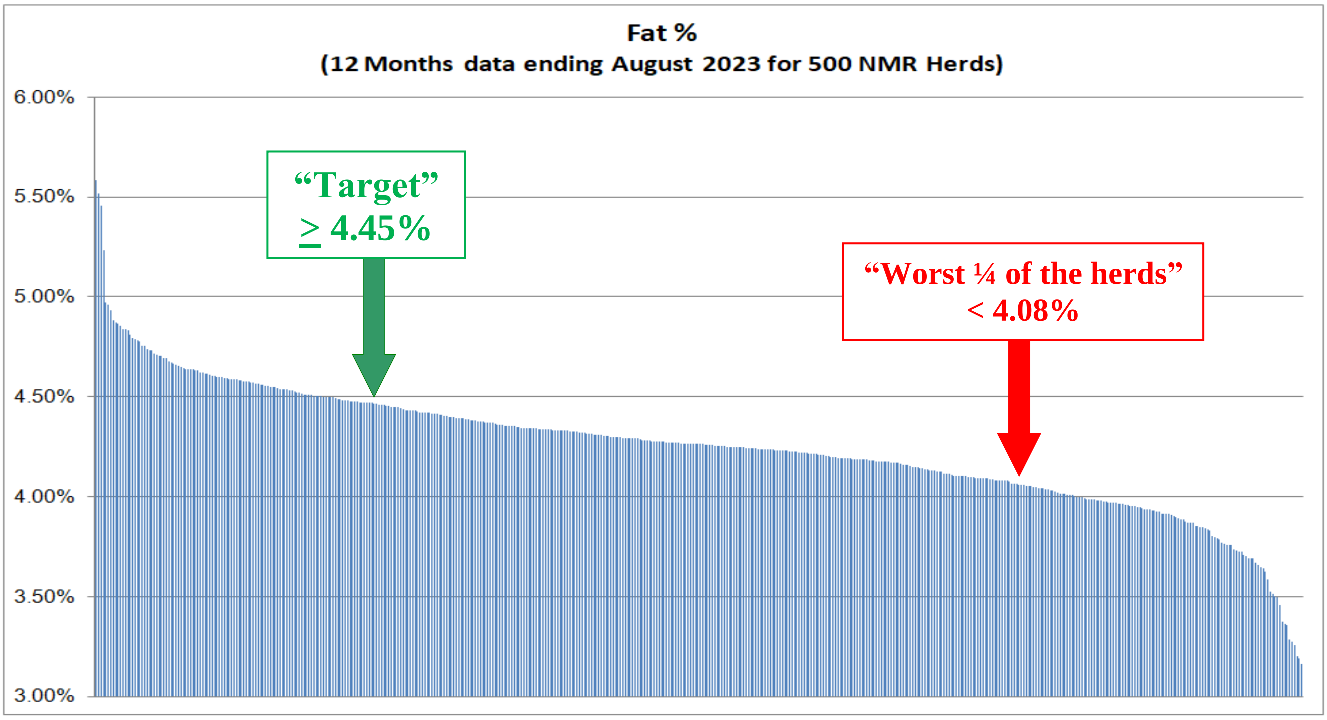

**S. 305 day yield (kg): The average yield of cows by day 305 of the lactation.**

| | |
|---|---|
| Target (top ¼ of herds' level): | 10,154 |
| Median: | 8,991 |
| 75% level: | 7,810 |
| Inter-quartile range: | 2,344 |

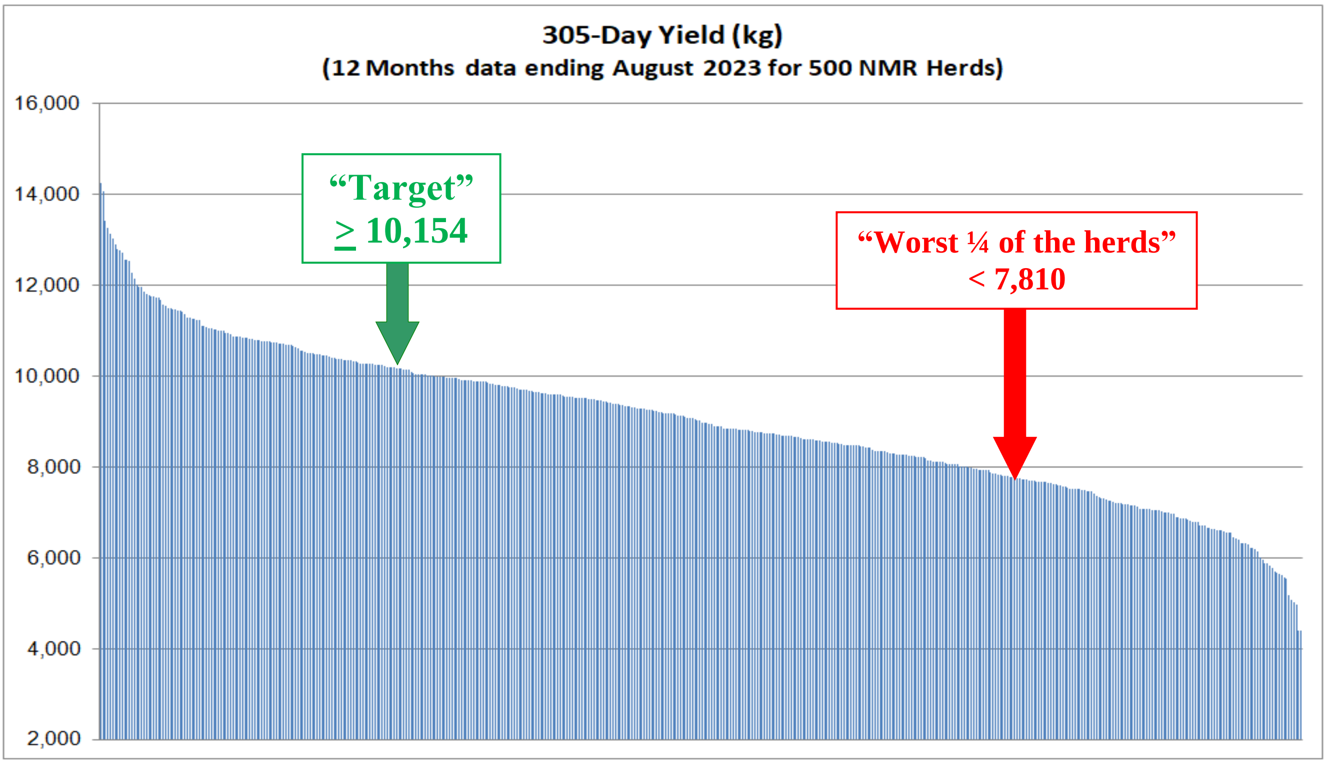


**T. 305 day protein (kg): The average milk protein yield of cows by day 305 of the lactation.**

| | |
|---|---|
| Target (top ¼ of herds' level): | 335 |
| Median: | 298 |
| 75% level: | 259 |
| Inter-quartile range: | 76 |

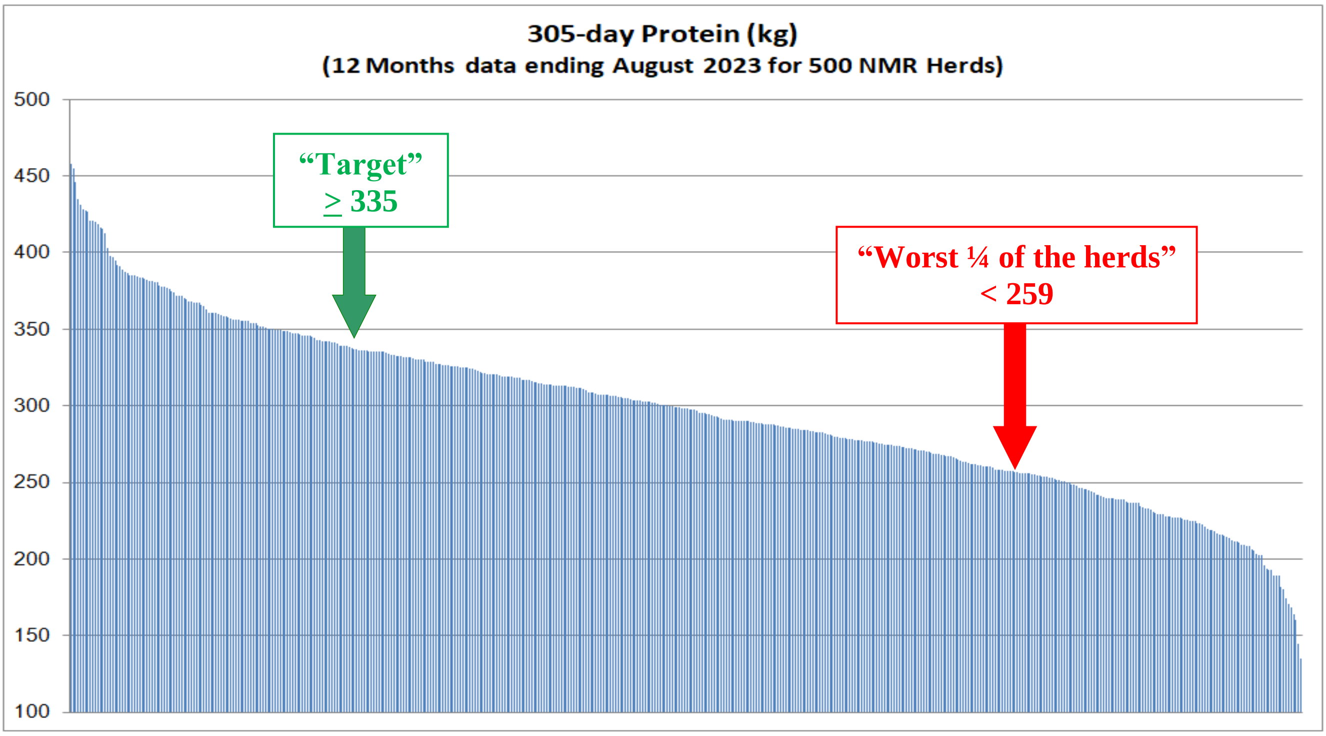

**U. 305 day fat (kg): The average milk fat yield of cows by day 305 of the lactation.**

| | |
|---|---|
| **Target (top ¼ of herds' level):** | **424** |
| Median: | 378 |
| 75% level: | 329 |
| Inter-quartile range: | 95 |

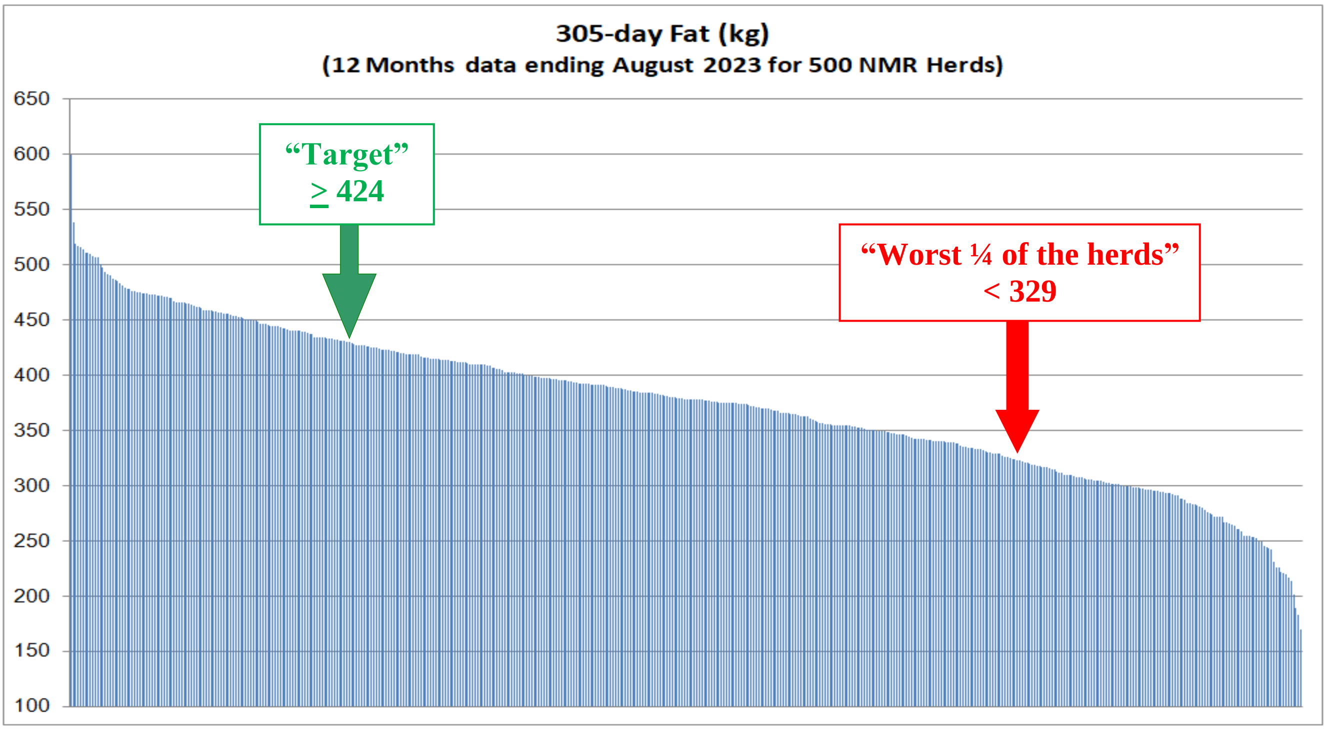


**V. Herd SCC ('000 cells/ml): The weighted average SCC of all milk samples taken in the last 12 months (weighted for volume of production of each cow producing the sample).**

| | |
|---|---|
| **Target (top ¼ of herds' level):** | **133** |
| Median: | 168 |
| 75% level: | 214 |
| Inter-quartile range: | 81 |

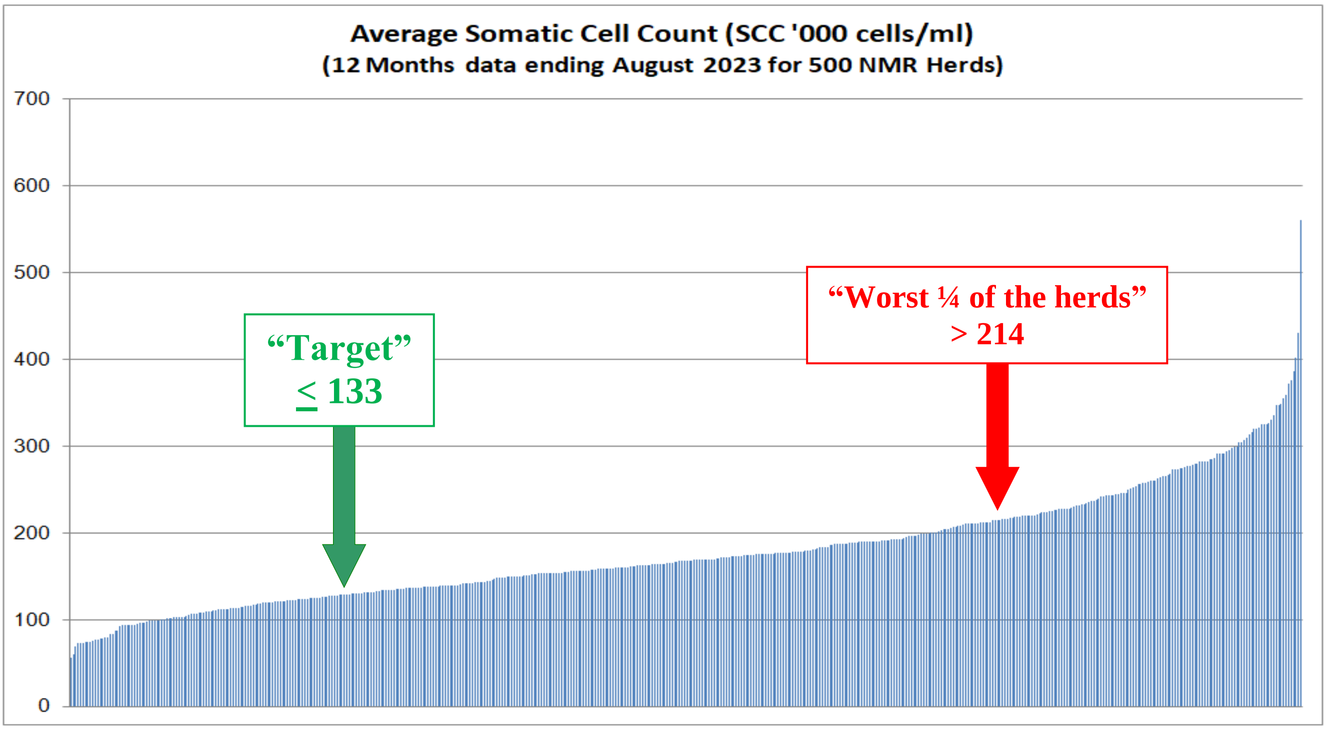

**W. % of milk samples with high SCC: The percentage of milk samples in the last 12 months with a SCC ≥ 200,000 cells/ml of milk. Indicates the size of any reservoir of infection.**

| | |
|---|---|
| **Target (top ¼ of herds' level):** | **12%** |
| Median: | 15% |
| 75% level: | 20% |
| Inter-quartile range: | 8% |

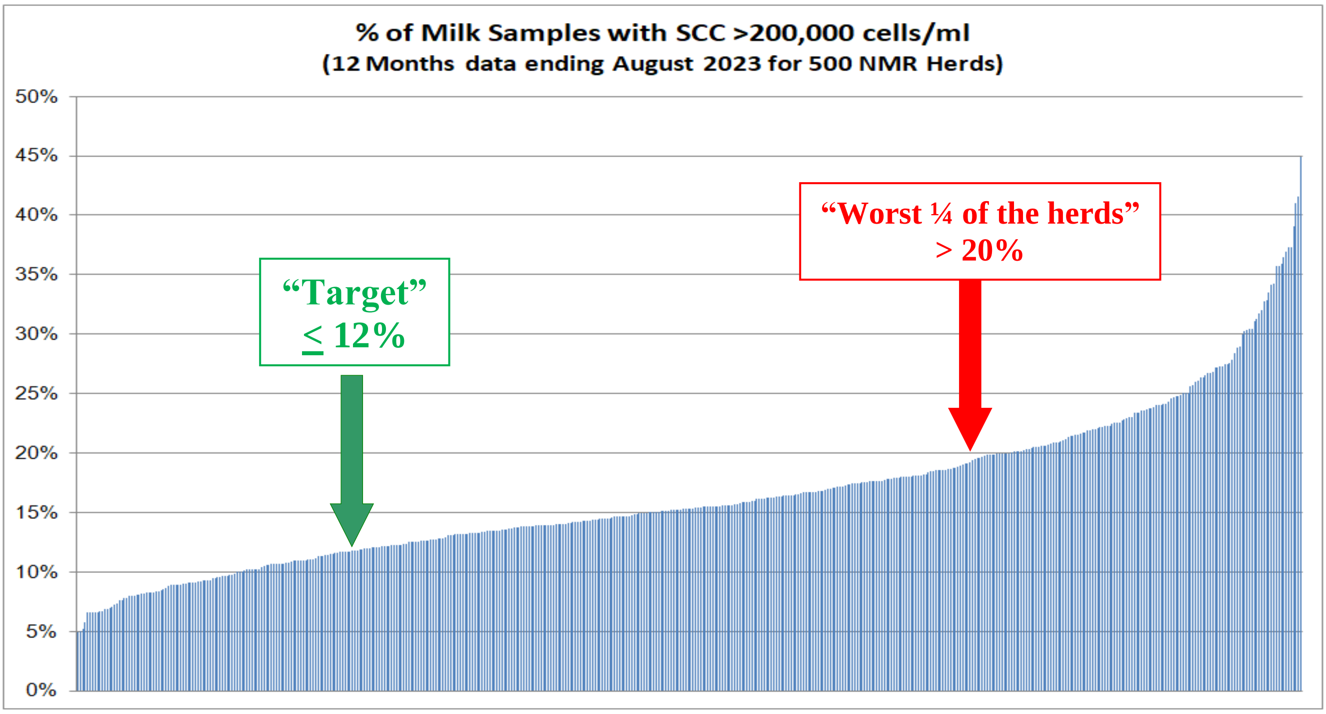


**X. % of milk samples with SCC ≥ 500,000 cells/ml: The percentage of milk samples taken in the last 12 months with a SCC ≥ 500,000 cells/ml of milk.**

| | |
|---|---|
| **Target (top ¼ of herds' level):** | **5%** |
| Median: | 7% |
| 75% level: | 9% |
| Inter-quartile range: | 4% |

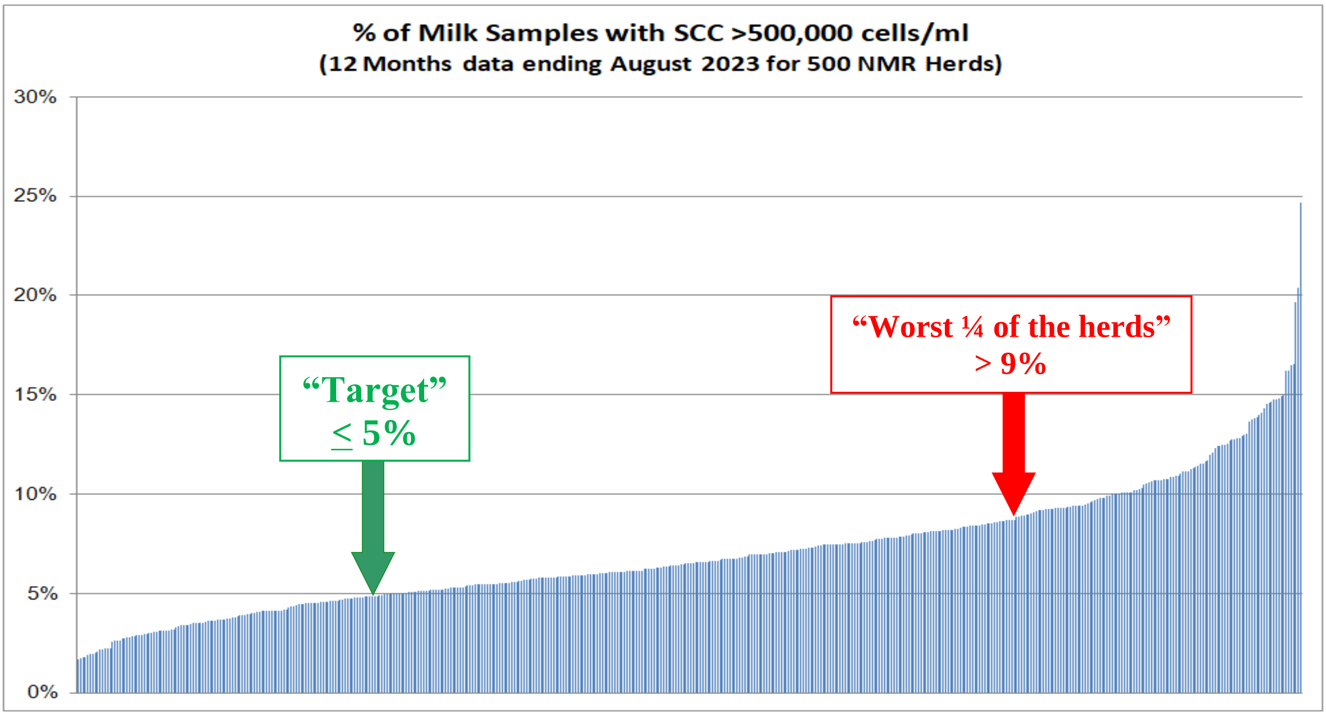

**Y. % 1st recording SCC ≥ 200,000 cells/ml: The percentage of new lactations in the last year starting with a high SCC (≥ 200,000 cells) at the first milk recording.**

| | |
|---|---|
| **Target (top ¼ of herds' level):** | **12%** |
| Median: | 15% |
| 75% level: | 20% |
| Inter-quartile range: | 8% |

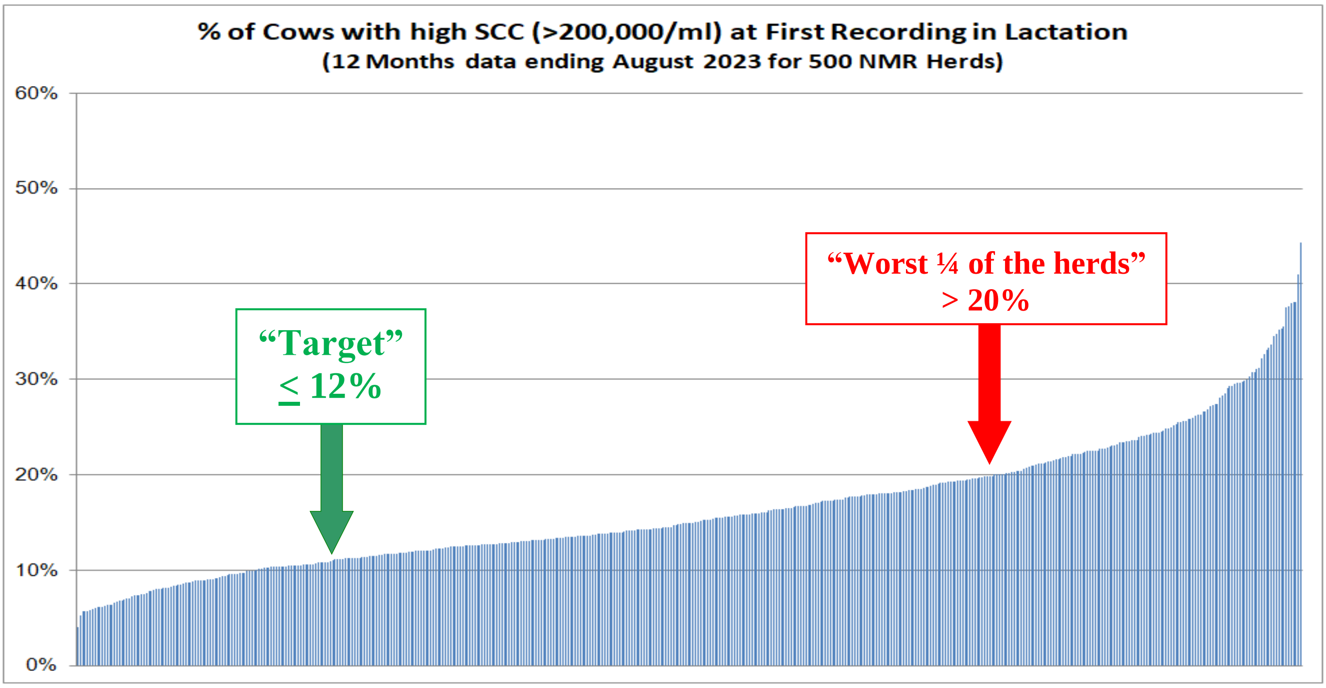


**Z. Percentage chronic SCC ≥ 200,000 cells/ml: The % of all milk samples taken over the last 12 months that were from CHRONIC cows (cows whose milk was ≥ 200,000 cells at both the CURRENT AND PREVIOUS milk recordings).**

| | |
|---|---|
| **Target (top ¼ of herds' level):** | **6%** |
| Median: | 8% |
| 75% level: | 11% |
| Inter-quartile range: | 5% |

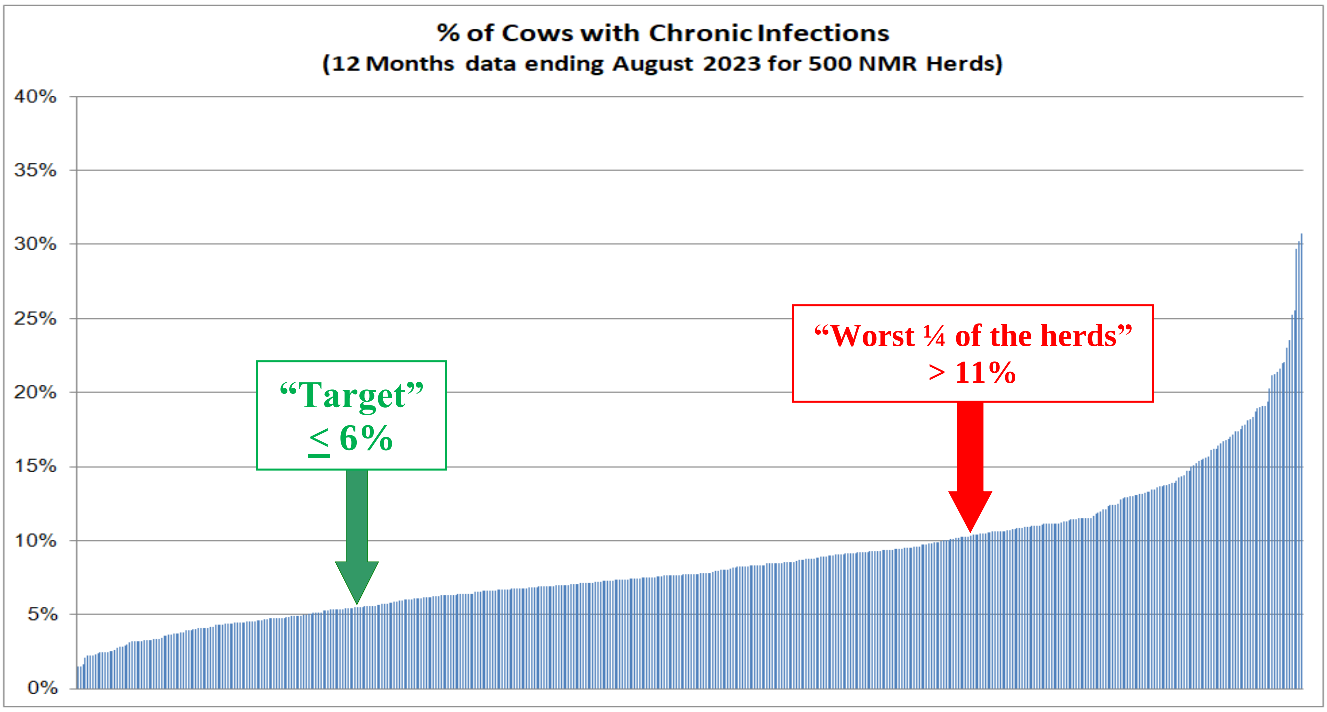

**ZA. Dry period cure (High:Low): The % of cows calving in the last year that ended their previous lactation with a high SCC (≥ 200,000 cells), started the new lactation with a LOW cell count (< 200,000 cells). The % of high SCC cows cured in the dry period.**

| | |
|---|---|
| Target (top ¼ of herds' level): | 86% |
| Median: | 77% |
| 75% level: | 69% |
| Inter-quartile range: | 17% |

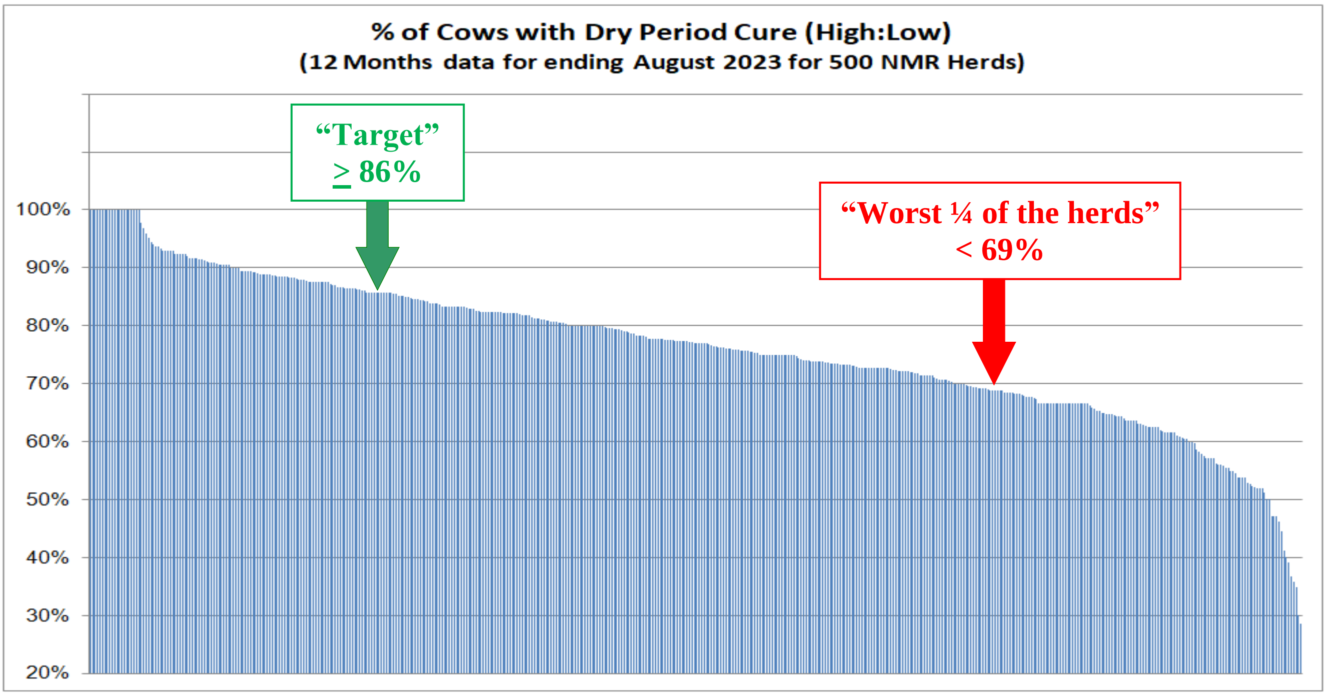


**ZB. Dry period protection (Low:Low): The % of cows calving in the last year that ended the previous lactation with a LOW SCC (< 200,000 cells) then started the new lactation with a LOW cell count (< 200,000 cells). The % of low SCC cows "protected" in the dry period.**

| | |
|---|---|
| Target (top ¼ of herds' level): | 90% |
| Median: | 86% |
| 75% level: | 80% |
| Inter-quartile range: | 10% |

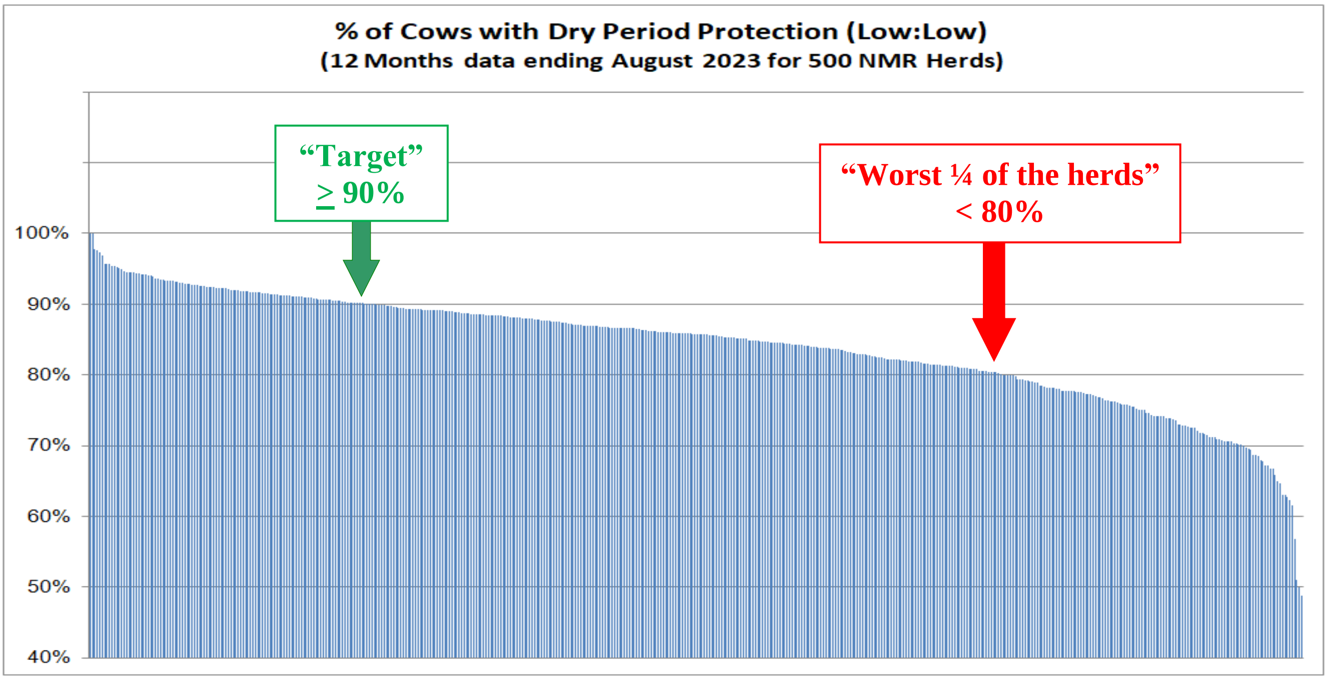

**ZC. % Low at the end of previous lactation: The percentage of cows calving in the last year that ended their previous lactation with a LOW SCC (< 200,000 cells).**

| | |
|---|---|
| **Target (top ¼ of herds' level):** | **84%** |
| Median: | 79% |
| 75% level: | 70% |
| Inter-quartile range: | 14% |

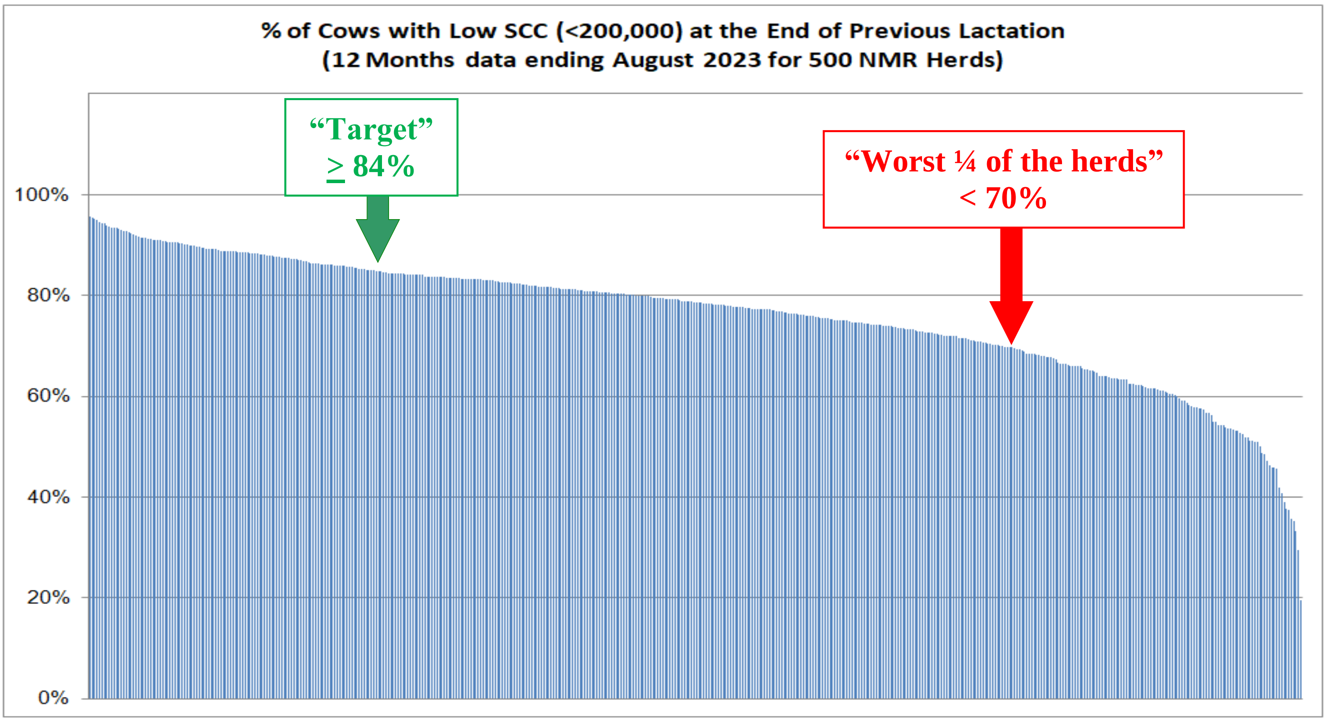


**ZD. % NEW SCC milk samples: Of all milk samples, the percentage that were of the NEW Herd Companion SCC Category (the first HIGH SCC (≥ 200,000cells/ml) in a lactation following one or more low SCC samples).**

| | |
|---|---|
| **Target (top ¼ of herds' level):** | **4%** |
| Median: | 5% |
| 75% level: | 7% |
| Inter-quartile range: | 3% |

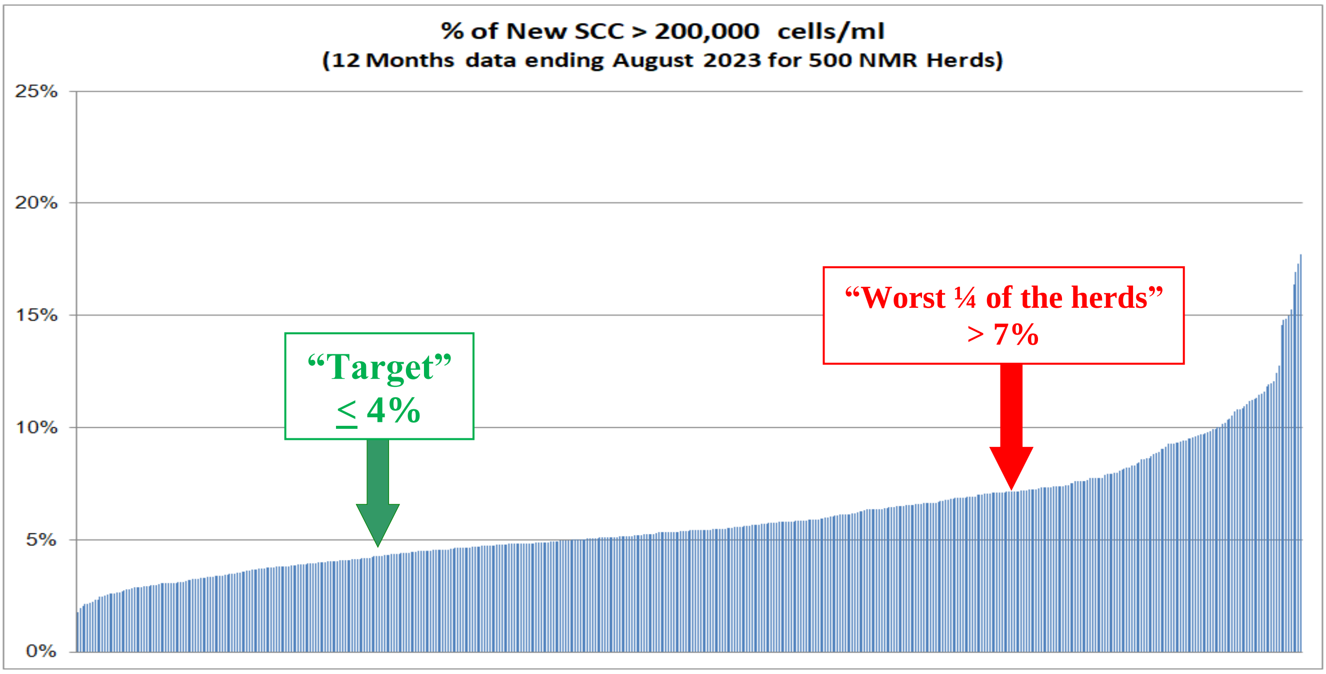

**ZE. % dried-off with no SCC ≥ 200,000 cells/ml: The percentage of cows recording only LOW SCC samples (< 200,000 cells/ml) in completed lactations.**

| | |
|---|---|
| **Target (top ¼ of herds' level):** | **59%** |
| Median: | 51% |
| 75% level: | 42% |
| Inter-quartile range: | 17% |

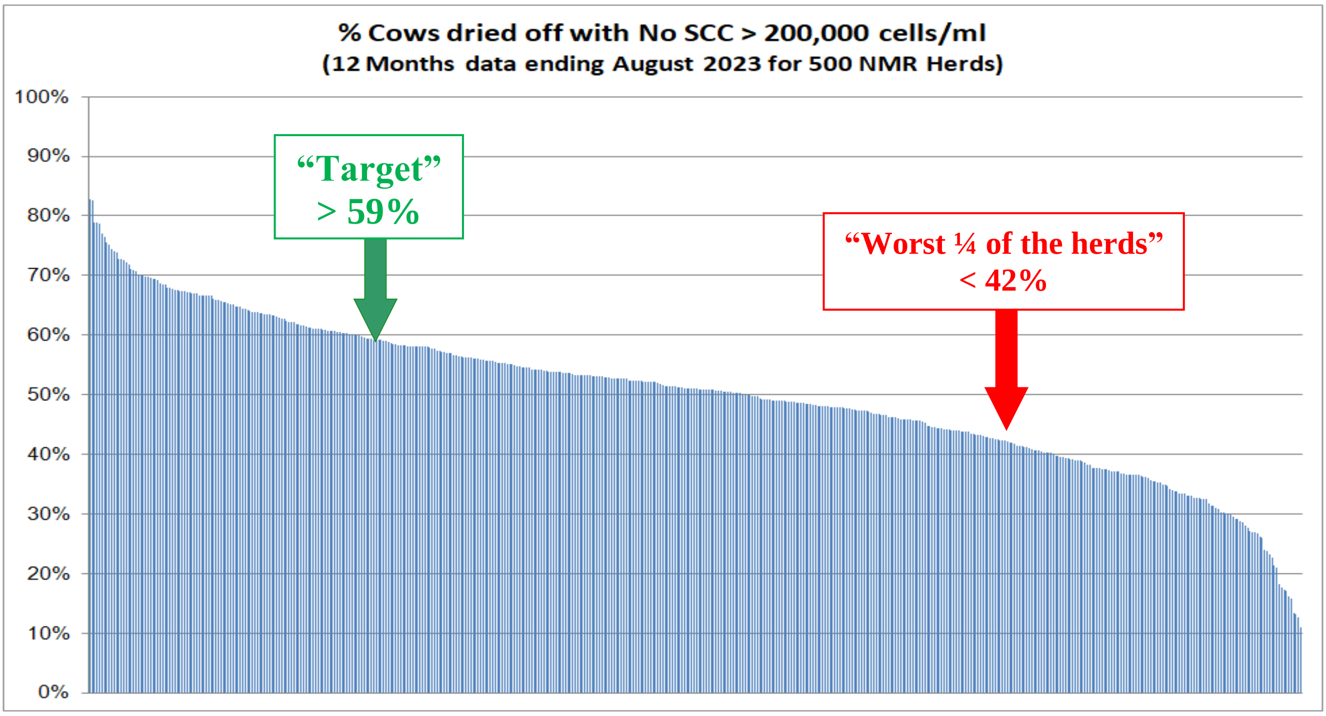


**ZF. Threshold Index new high / new low: The total cows changing from Low to High SCC divided by the total cows changing from High to Low SCC at consecutive recordings.**

| | |
|---|---|
| **Target (top ¼ of herds' level):** | **1.07** |
| Median: | 1.20 |
| 75% level: | 1.32 |
| Inter-quartile range: | 0.25 |

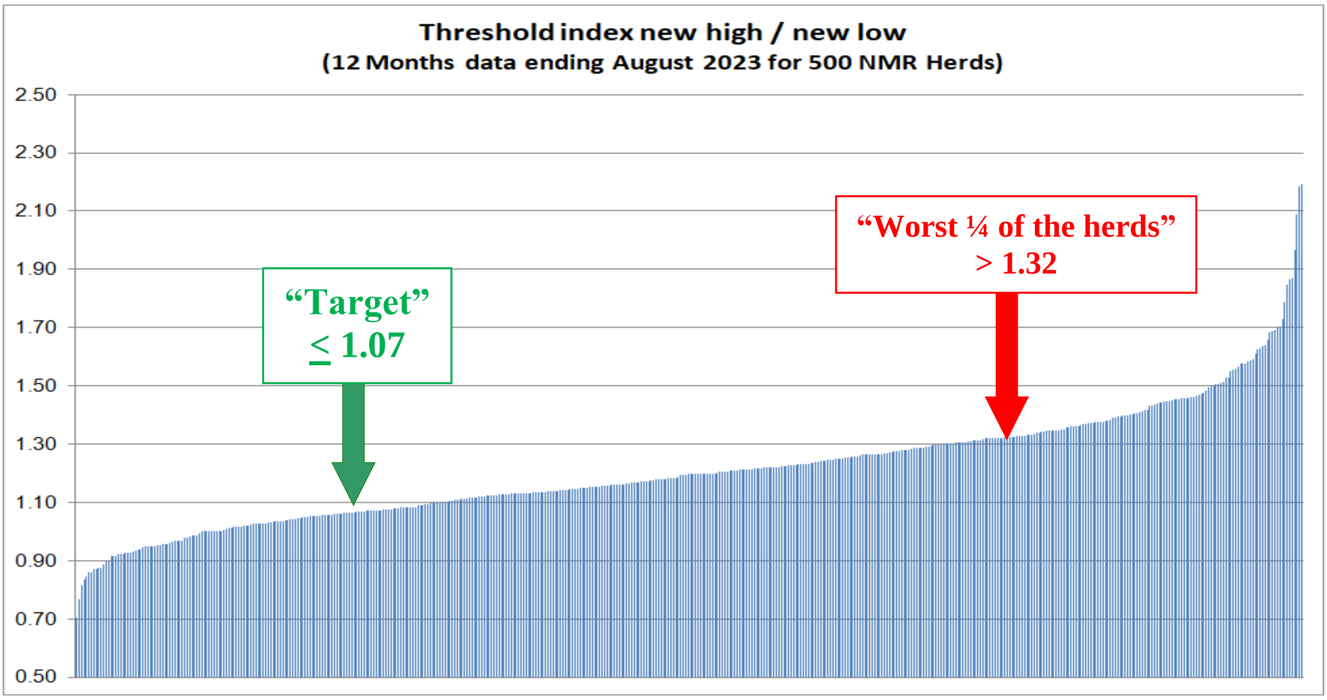

**ZG. Recovery % of New/First/Repeat infections: Of HIGH SCC cows (≥ 200,000cells/ml) that at the previous recording were either low SCC or not yet in milk, the percentage that were LOW SCC (< 200,000 cells/ml) at the following recording.**

| | |
|---|---|
| **Target (top ¼ of herds' level):** | **62%** |
| Median: | 57% |
| 75% level: | 52% |
| Inter-quartile range: | 10% |

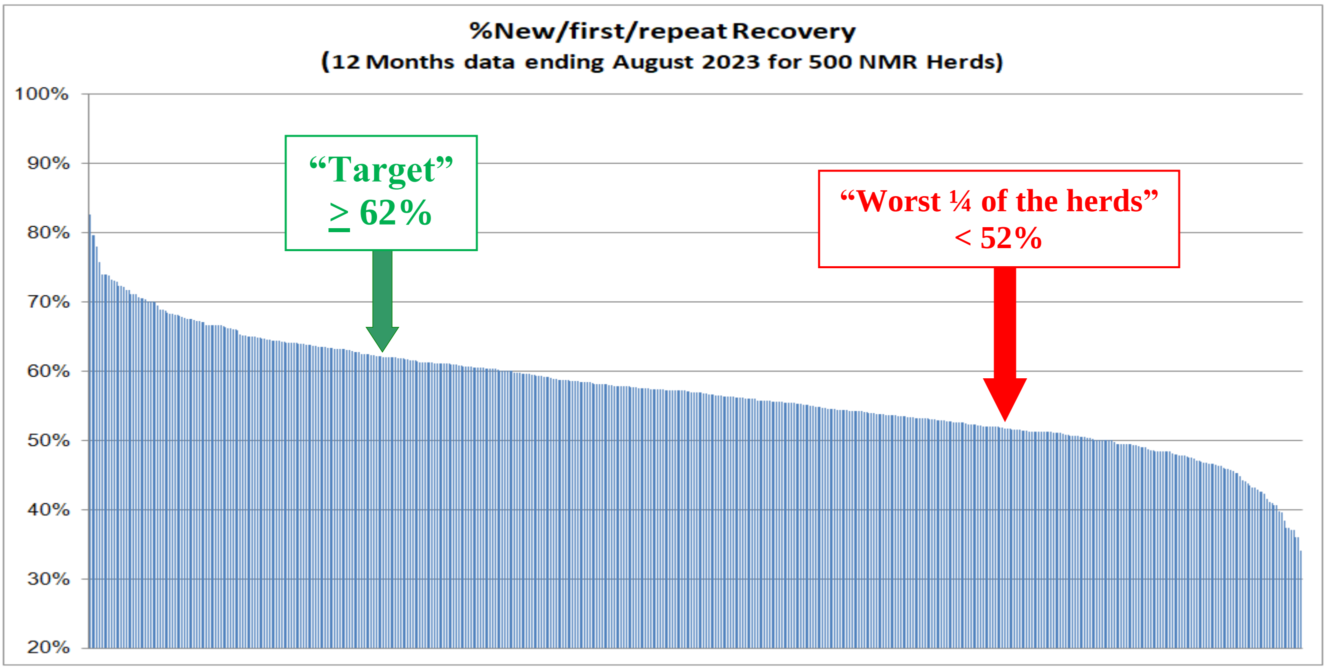


**ZH. Recovery % of chronic infections: Of cows with two or more consecutive HIGH SCC recordings (≥ 200,000 cells/ml), the percentage that recorded a LOW SCC (< 200,000 cells/ml) at the following recording.**

| | |
|---|---|
| **Target (top ¼ of herds' level):** | **26%** |
| Median: | 21% |
| 75% level: | 17% |
| Inter-quartile range: | 9% |

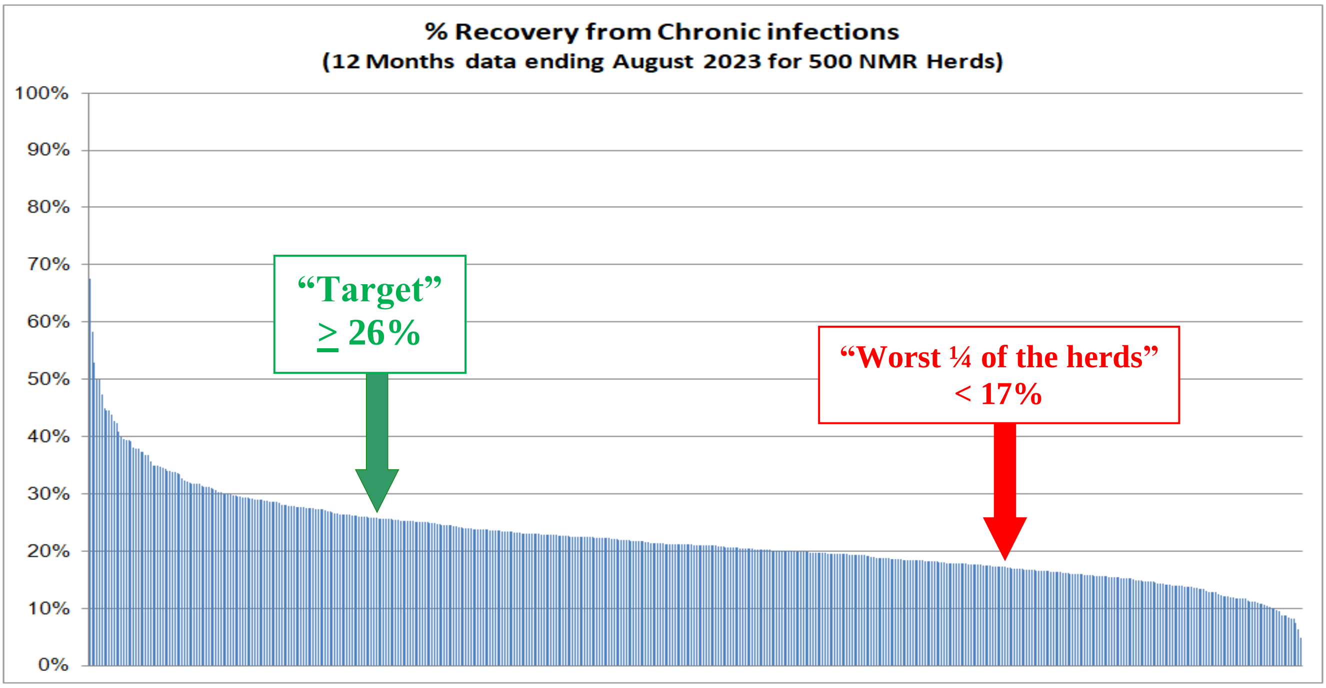

**ZJ. Mastitis rate: Number of clinical mastitis cases per 100 cows in milk in the herd over a year.**

| | |
|---|---|
| Target (top ¼ of herds' level): | 13 |
| Median: | 22 |
| 75% level: | 36 |
| Inter-quartile range: | 23 |

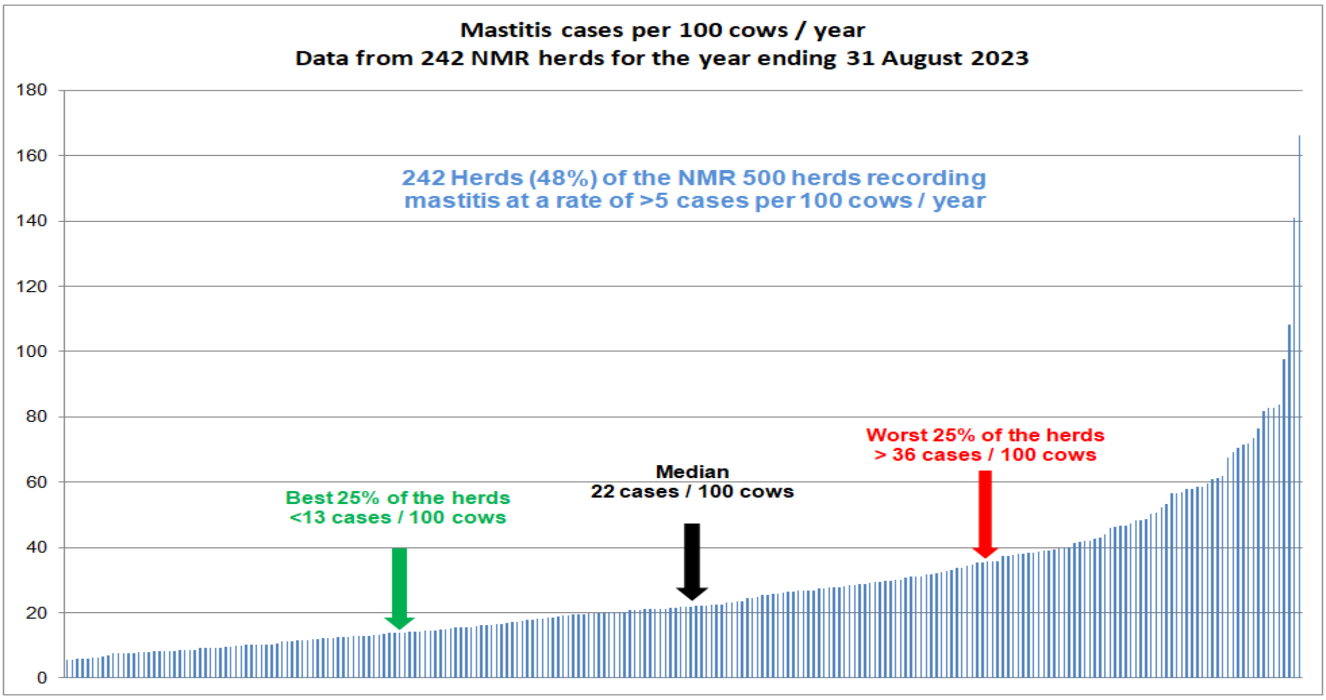

# Section 3: Trends in KPIs from 2010 to 2023

## 3.1 Summary of trends since 2010.

From 2010 to 2023, the median herd size increased by 38% from 129 to 178 cows. Herd size increased steadily to 2018 and since fluctuated between 169 and 178 cows. Productive life decreased by 12% from 1,511 days in 2010 to 1,323 days in 2021. Productive life improved slightly to 1,359 days in 2022 and 1,367 in 2023 but remains 10% below 2010 levels. Age at exit decreased from 6.6 to 6.0 years. Age at exit was stable in 2011 and 2012, steadily decreased from 6.5 years in 2013 to 5.9 years in 2021 and improved slightly to 6.0 years in 2022 and 2023. Cull rates increased from 24% to 28%. Cull rates increased from 2011 to 2013 but returned to 2010 levels, before increasing with fluctuations from 2016 onwards.

Age at first calving was stable from 2011 to 2014 at 2.4 years, but decreased in 2015 to 2.3 years and again in 2021 to 2.2 years, an overall decrease of 8%. The % served by 80 DPP increased from 46% to 60% between 2010 and 2017 and has since fluctuated between 57% and 61%. The % service intervals 18-24 days increased with fluctuations from 30% in 2010 to 41% in 2022 and 2023. Calving to 1st service interval decreased from 105 days in 2010 to 81 days in 2017 and has since fluctuated between 79 days and 81 days, an overall reduction of 23%. The % conceived 100 DPP steadily increased from 26% in 2010 to 39% in 2022 and 2023. Conception rate increased from 32% to 39%. Conception rate decreased in 2011 and 2012 but has increased since 2016, especially in 2021, 2022 and 2023. Calving interval has gradually decreased by 7% from 424 in 2010 to 394 in 2022 and 2023.

Milk/cow/year increased with fluctuations from 7,665 kg in 2010 to 9,008 kg in 2021. Milk/cow/year was slightly lower in 2022 at 8,708 kg and in 2023 at 8,737 kg. Lifetime milk/cow/day increased by 25%, albeit with fluctuations, from 10.5kg in 2010 to 13.1 kg in 2021. Lifetime milk/cow/day was slightly lower in 2022 and 2023 at 12.7 kg. Milk protein fluctuated between 3.24% and 3.30% from 2011 and 2018, but a small, consistent increase has been present since 2019, whereby milk protein has increased to 4.26%. Milk fat fluctuated between 3.96% and 4.03% until 2019, but a relatively large increase was observed from 2020 onwards up to 4.26% in 2023.

Average SCC decreased by 20% from 210,000 cells/ml in 2010 to 168,000 cells/ml in 2023. The proportion of recorded cows with SCC >=200,000 cells/ml decreased gradually from 24% in 2010 to 15% in 2022. The proportion of recorded cows with SCC >=500,000 cells/ml decreased gradually from 9% in 2010 to 7% in 2015. The proportion has remained stable at 7% for the past 8 years. The proportion of recorded cows with chronic (repeat) SCC >=200,000 cells/ml decreased gradually from 14% in 2010 to 8% in 2020, it has been stable for the past 4 years.

Table Trends 1 and 2 below shows changes in the median and target (top 25% performance) values for each parameter in 2010, 2022 and 2023. The majority of parameters have improved (**green**) over the period with the exception of overall culling, age and number of lactations at exit which show some deterioration (**red**). Some of the parameters have continued to improve since last year (2022). Median values for conception rate, milk per cow/year, protein & fat %, %SCC >=200,000 cells/ml and %Low SCC (<200,000 cells/ml) at end of previous lactation, indicate slight improvement in 2023 compared to figures from 2022.

**Table Trends 1. Comparison of longevity and fertility median and target values derived from the study of 500 NMR recording herds in 2022 and 2023 with the original study in 2010.**

| Parameter | Median | | | Target "Best 25%" | | |
|---|---|---|---|---|---|---|
| ***Year of the Study*** | ***2010*** | ***2022*** | ***2023*** | ***2010*** | ***2022*** | ***2023*** |
| A. Culling rate | 24% | 26% | 28% | 18% | 22% | 22% |
| B. Percentage culled (off take) / died 100 days after calving | 7% | 5% | 5% | 4% | 3% | 3% |
| C(a). Age at exit (years) | 6.6 | 6.0 | 6.0 | 7.4 | 6.7 | 6.7 |
| C(b). Age at exit (days) | 2,393 | 2,180 | 2,179 | 2,699 | 2,442 | 2,433 |
| D. Age at exit by lactations | 3.9 | 3.6 | 3.6 | 4.5 | 4.1 | 4.2 |
| E. Percentage Served by day 80 | 46% | 60% | 59% | 59% | 70% | 69% |
| F. Percentage conceived 100 days after calving | 26% | 39% | 39% | 33% | 46% | 46% |
| G. Calving to 1st service interval (days) | 105 | 80 | 81 | 87 | 70 | 71 |
| H. Calving interval (days) | 424 | 394 | 394 | 409 | 384 | 383 |
| I(a). Age at 1st calving (years) | 2.4 | 2.2 | 2.2 | 2.3 | 2.1 | 2.1 |
| I(b). Age at 1st calving (days) | 894 | 799 | 804 | 828 | 756 | 761 |
| J. Conception rate | 32% | 38% | 39% | 40% | 45% | 45% |
| K. %Service intervals at 18-24 days (Heat detection) | 30% | 41% | 41% | 38% | 47% | 48% |
| L. %Service intervals >50 days | 32% | 19% | 19% | 22% | 13% | 12% |
| M. %Cows eligible for service served (Submission rate) | 27% | 41% | 41% | 37% | 53% | 53% |

**Table Trends 2. Comparison of milk, composition and SCC median and target values derived from the study of 500 NMR recording herds in 2022 and 2023 with the original study in 2010.**

| Parameter | Median | | | Target "Best 25%" | | |
|---|---|---|---|---|---|---|
| ***Year of the Study*** | ***2010*** | ***2022*** | ***2023*** | ***2010*** | ***2022*** | ***2023*** |
| O. Lifetime milk / cow / day (kg) | 10.5 | 12.7 | 12.7 | 12.6 | 14.7 | 14.6 |
| P. Milk / cow / year (kg) | 7,665 | 8,708 | 8,737 | 8,760 | 9,763 | 9,961 |
| Q. Average protein% | 3.27% | 3.33% | 3.36% | 3.33% | 3.40% | 3.43% |
| R. Average fat% | 3.96% | 4.18% | 4.26% | 4.12% | 4.34% | 4.45% |
| V. Average SCC ('000 cells/ml) | 210 | 166 | 168 | 169 | 130 | 133 |
| W. % SCC ≥200,000 cells/ml | 24% | 16% | 15% | 19% | 12% | 12% |
| X. % SCC ≥500,000 cells/ml | 9% | 7% | 7% | 7% | 5% | 5% |
| Y. % 1st recording SCC ≥200,000 cells/ml | 20% | 15% | 15% | 15% | 12% | 12% |
| Z. % chronic SCC ≥200,000 cells/ml | 14% | 8% | 8% | 10% | 6% | 6% |
| ZA. % Dry period cure (High:Low) | 74% | 78% | 77% | 80% | 84% | 86% |
| ZB. % Dry period protection (Low:Low) | 84% | 86% | 86% | 89% | 90% | 90% |
| ZC. % Low at end of previous lactation (SCC<200,000 cells/ml) | 60% | 78% | 79% | 70% | 84% | 84% |

## 3.2 Trends in herd size since 2010.

From 2010 to 2023, the median herd size increased by 38% from 129 to 178 cows. Herd size increased steadily to 2018 and since fluctuated between 169 and 178 cows (Figure 1).

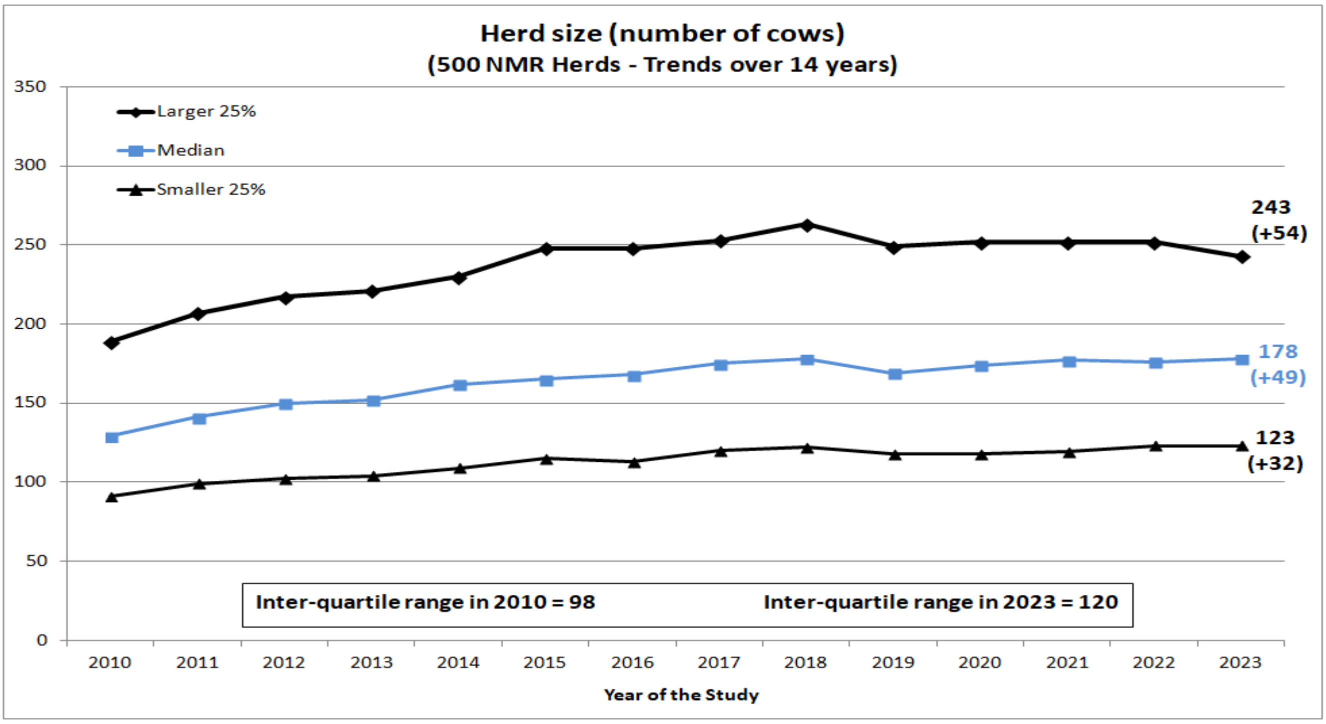


**Figure 1. Herd size (number of cows).**

The figures below show the changes over the fourteen annual KPI studies (2010 to 2023) for a number of important parameters. The three lines represent the "better" quartile, median and "poorer" quartile values each year for each parameter.

## 3.3 Trends in culling and longevity parameters since 2010.

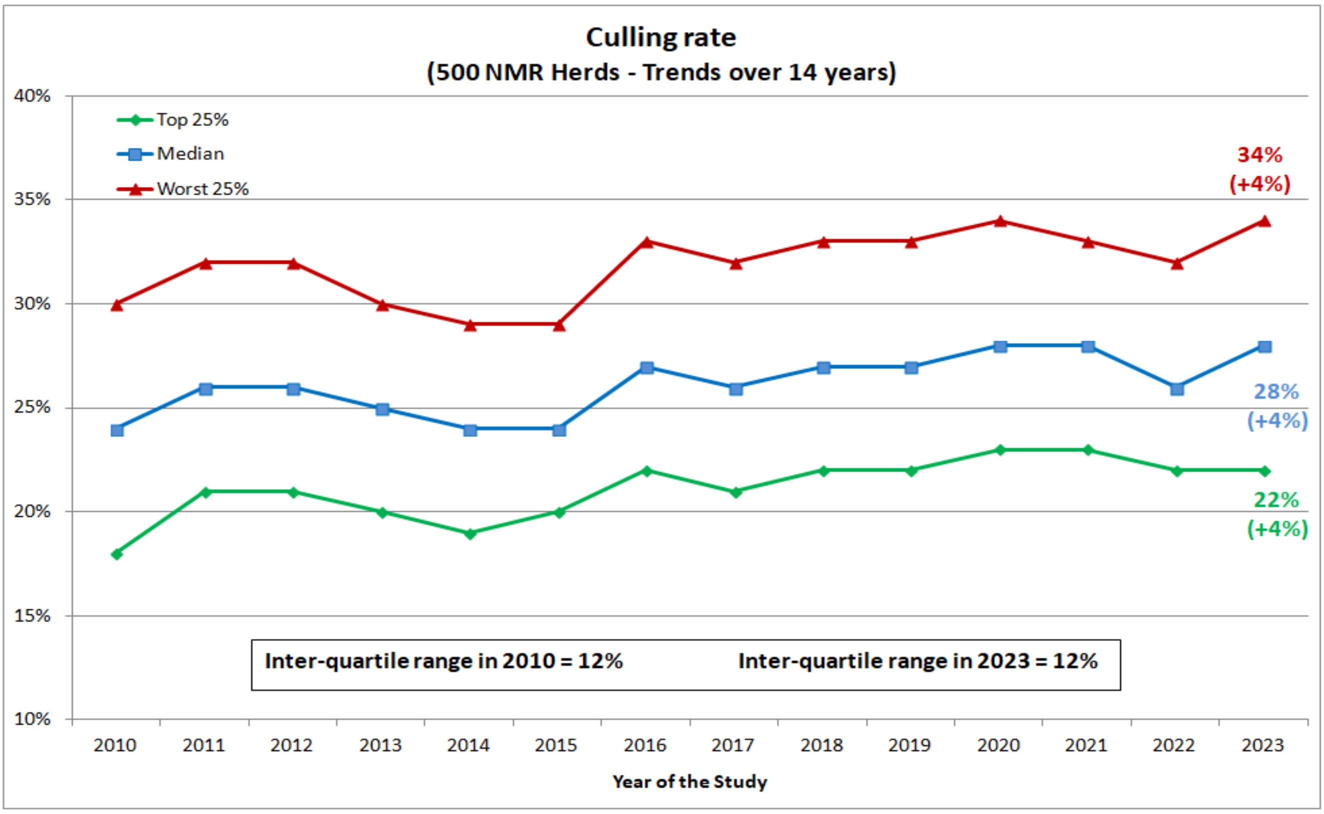


**Figure 2. % of cows which exited the herd (culled, died or sold).**

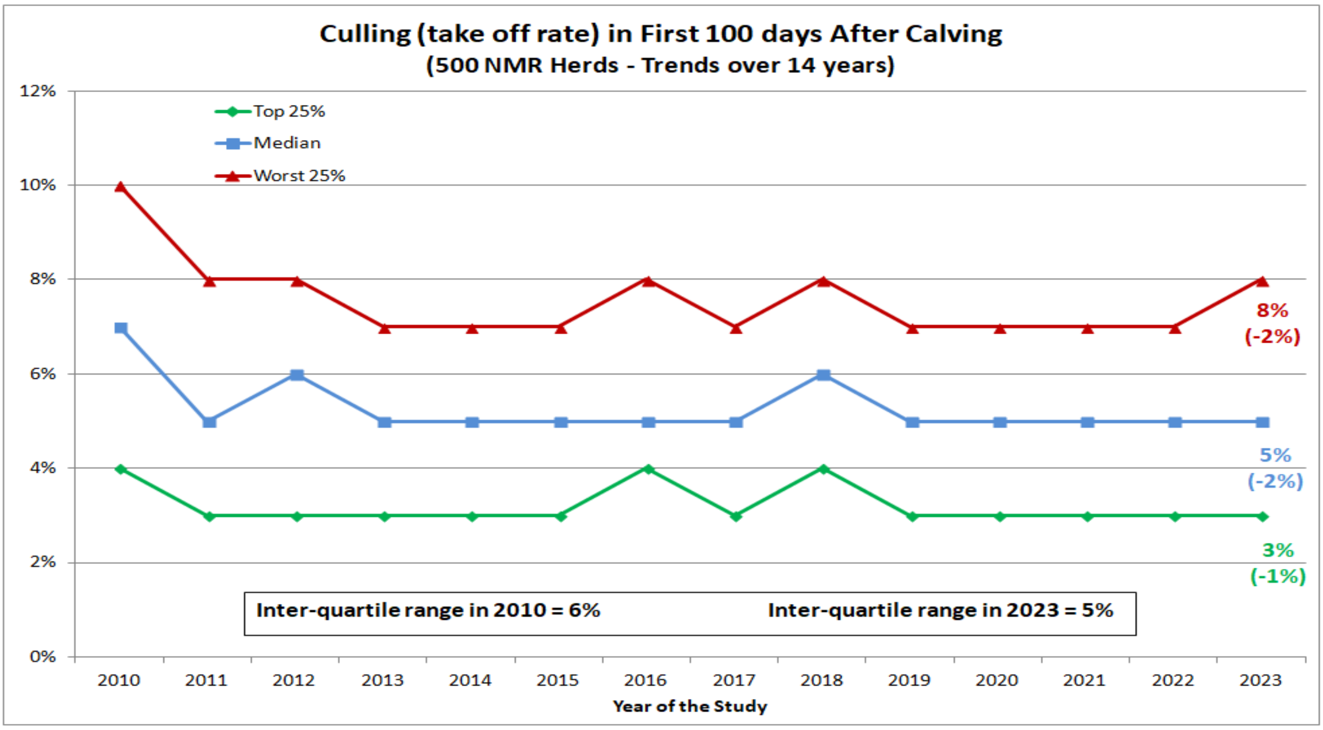


**Figure 3. % of cows which exited the herd (culled, died or sold) within 100 DPP.**

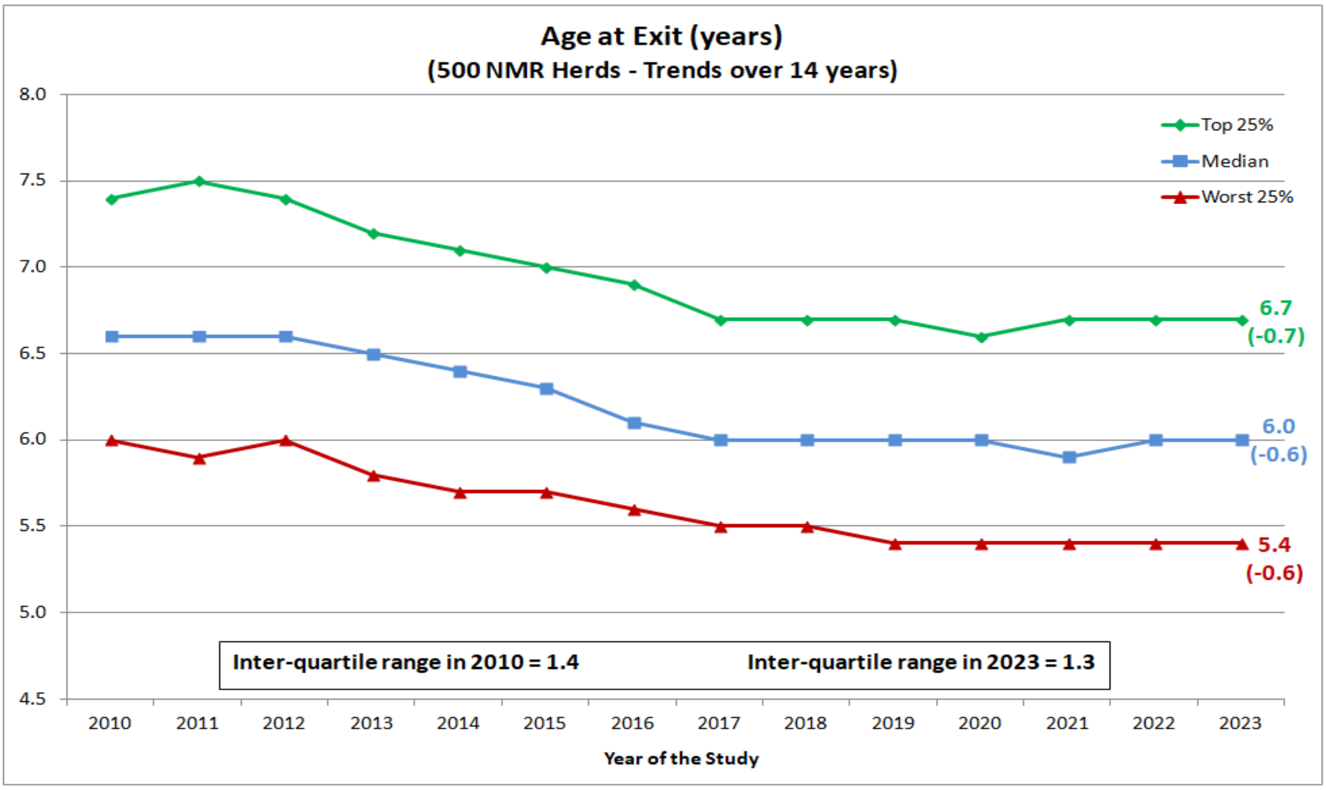


**Figure 4. Age at exit (years).**

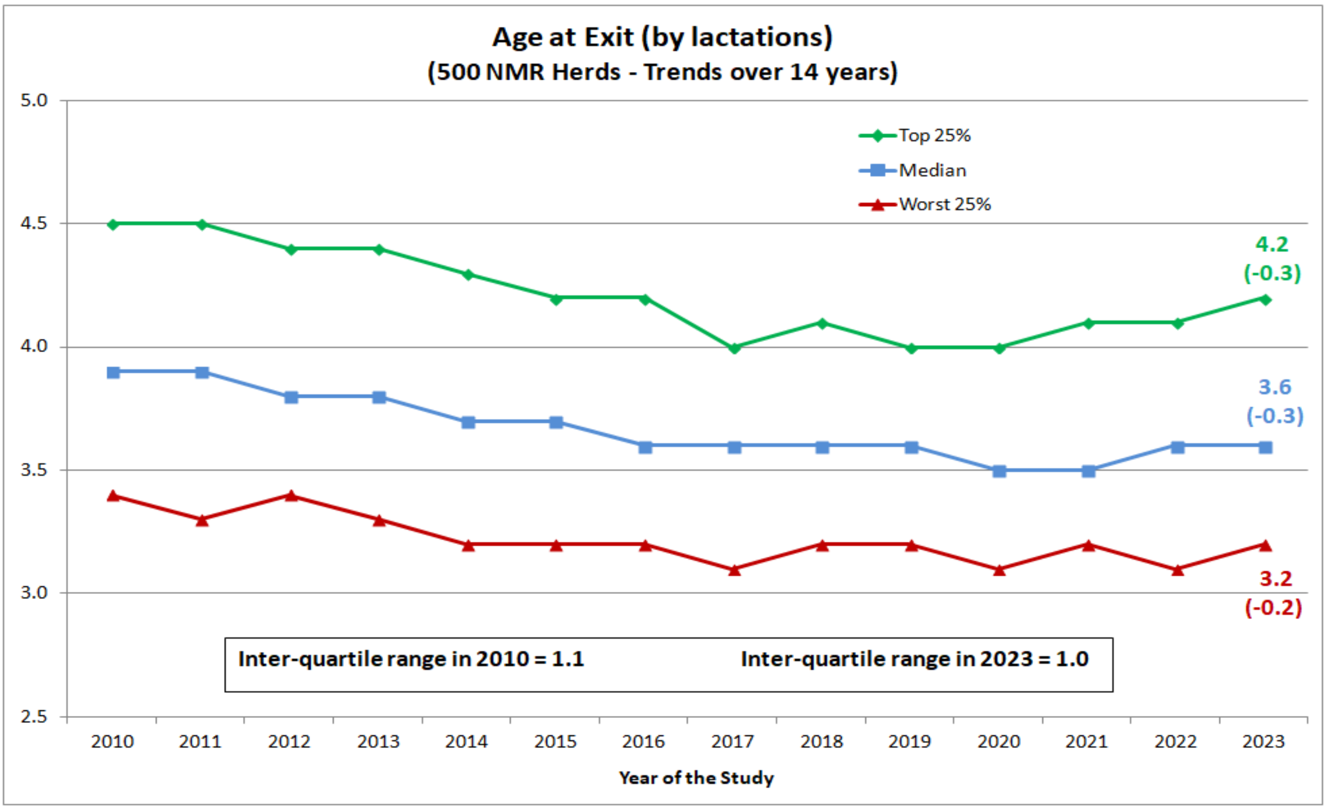


**Figure 5. Age at exit (lactations).**

## 3.4 Trends in key fertility parameters since 2020.

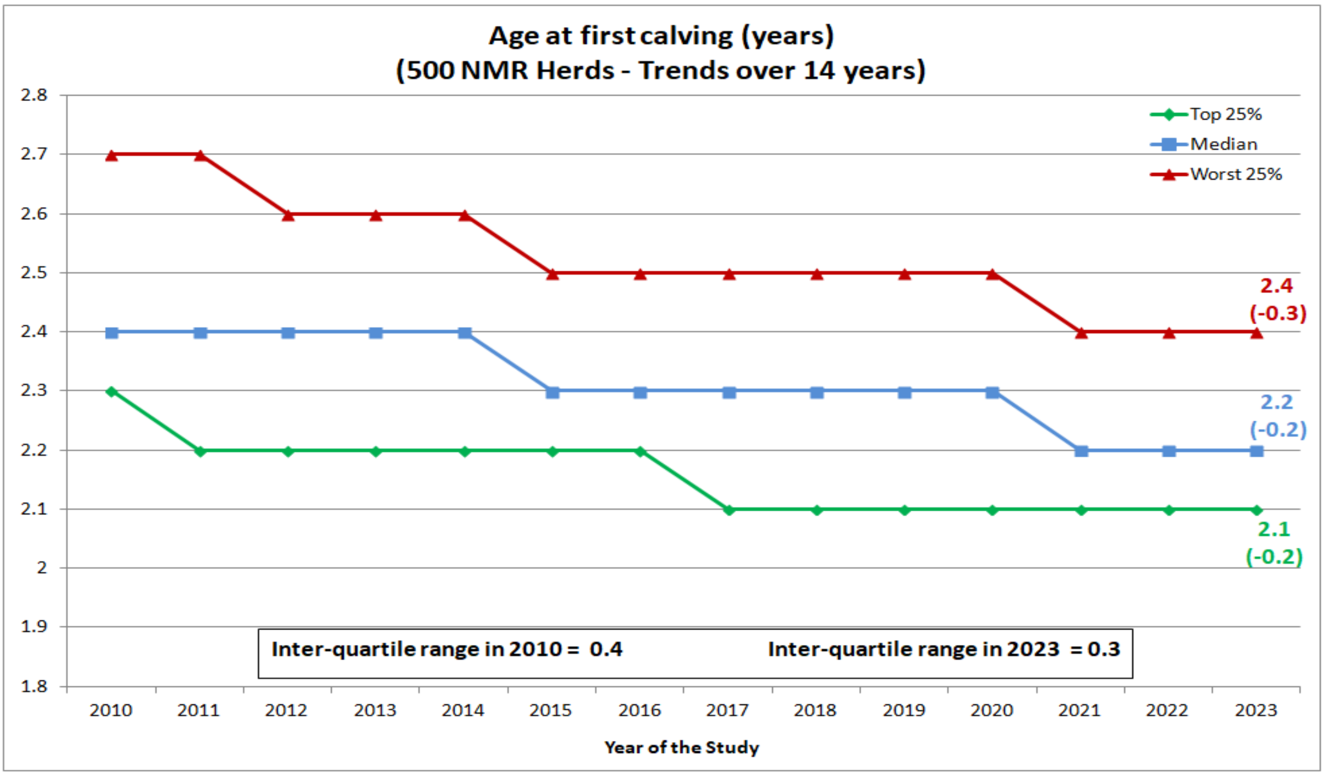


**Figure 6. Age at first calving (years).**

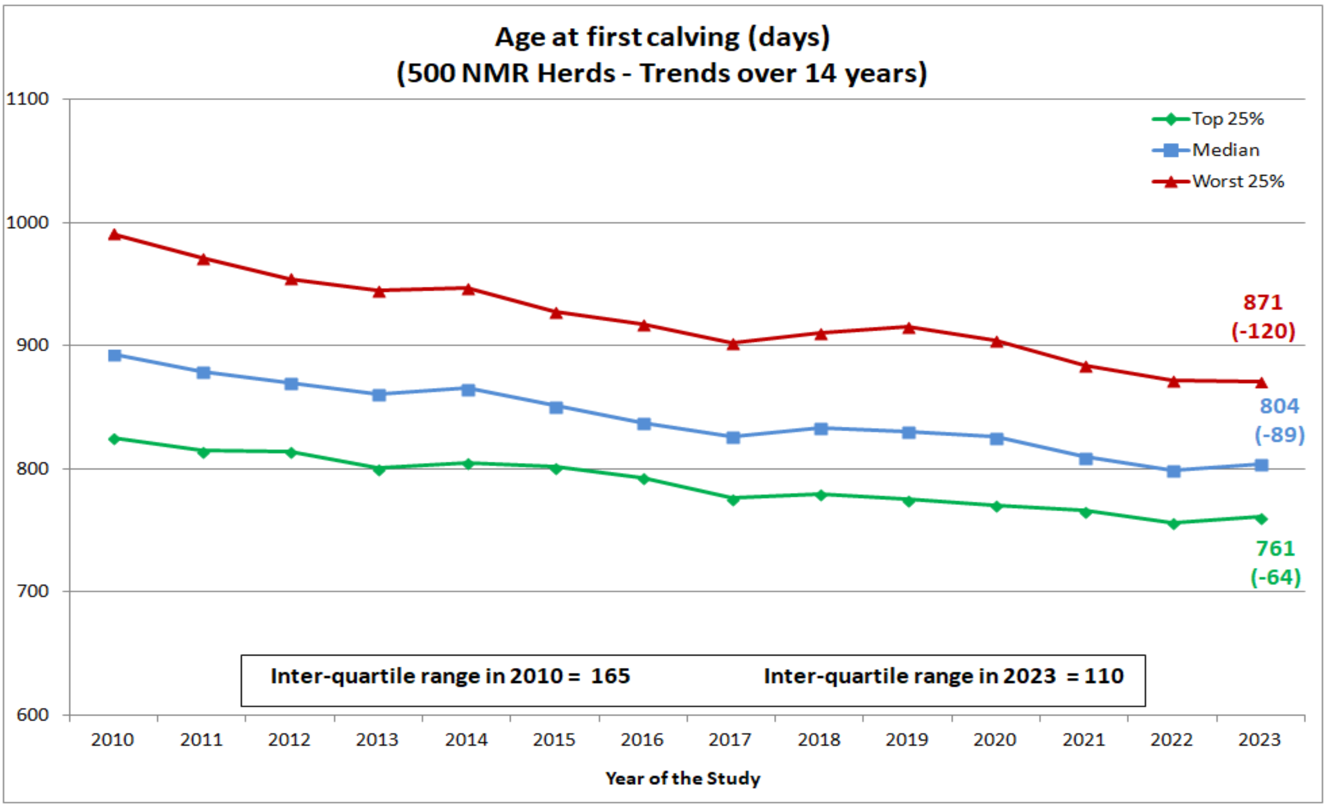


**Figure 7. Age at first calving (days).**

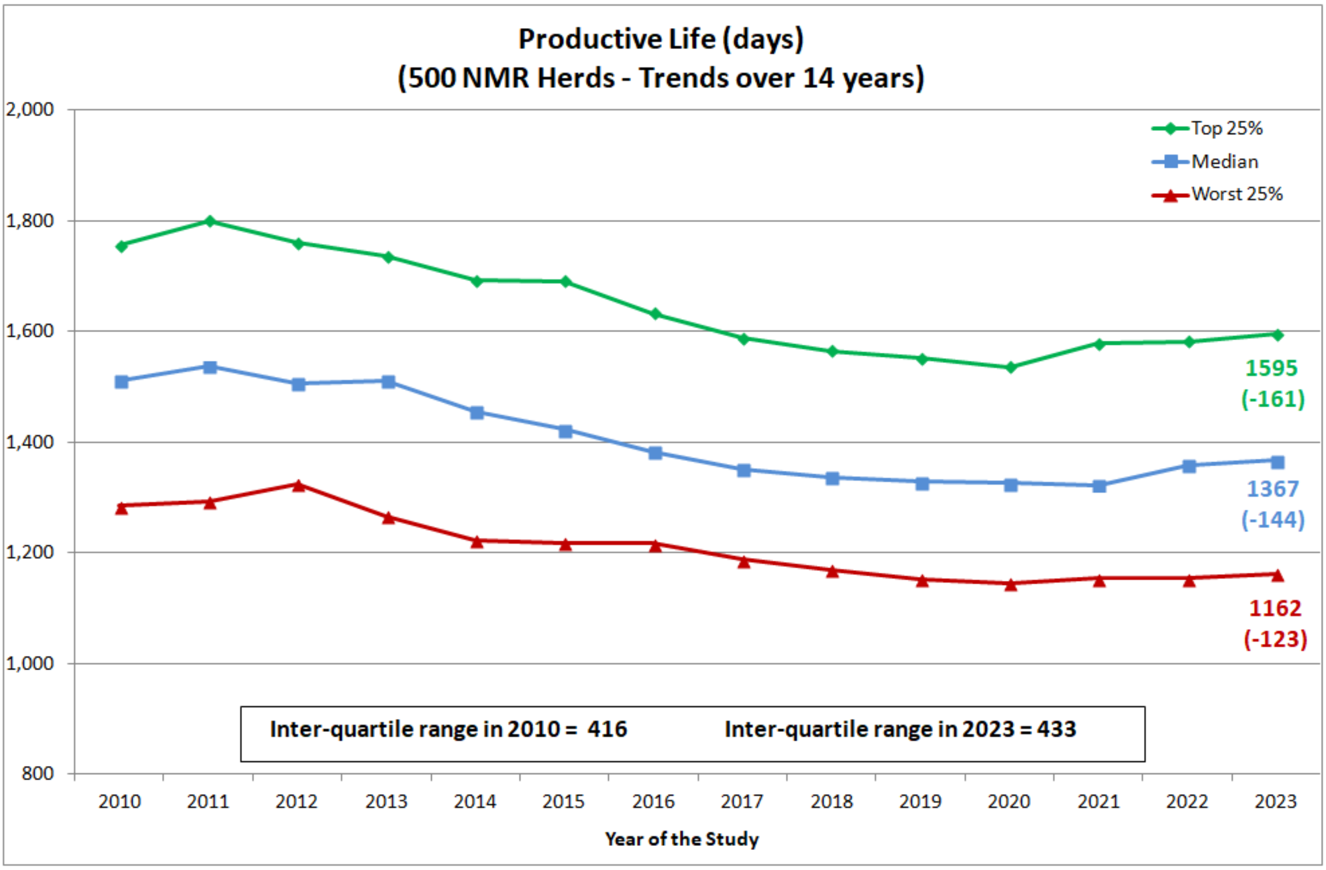


**Figure 8. Productive life (age at exit - age at 1st calving) (days).**

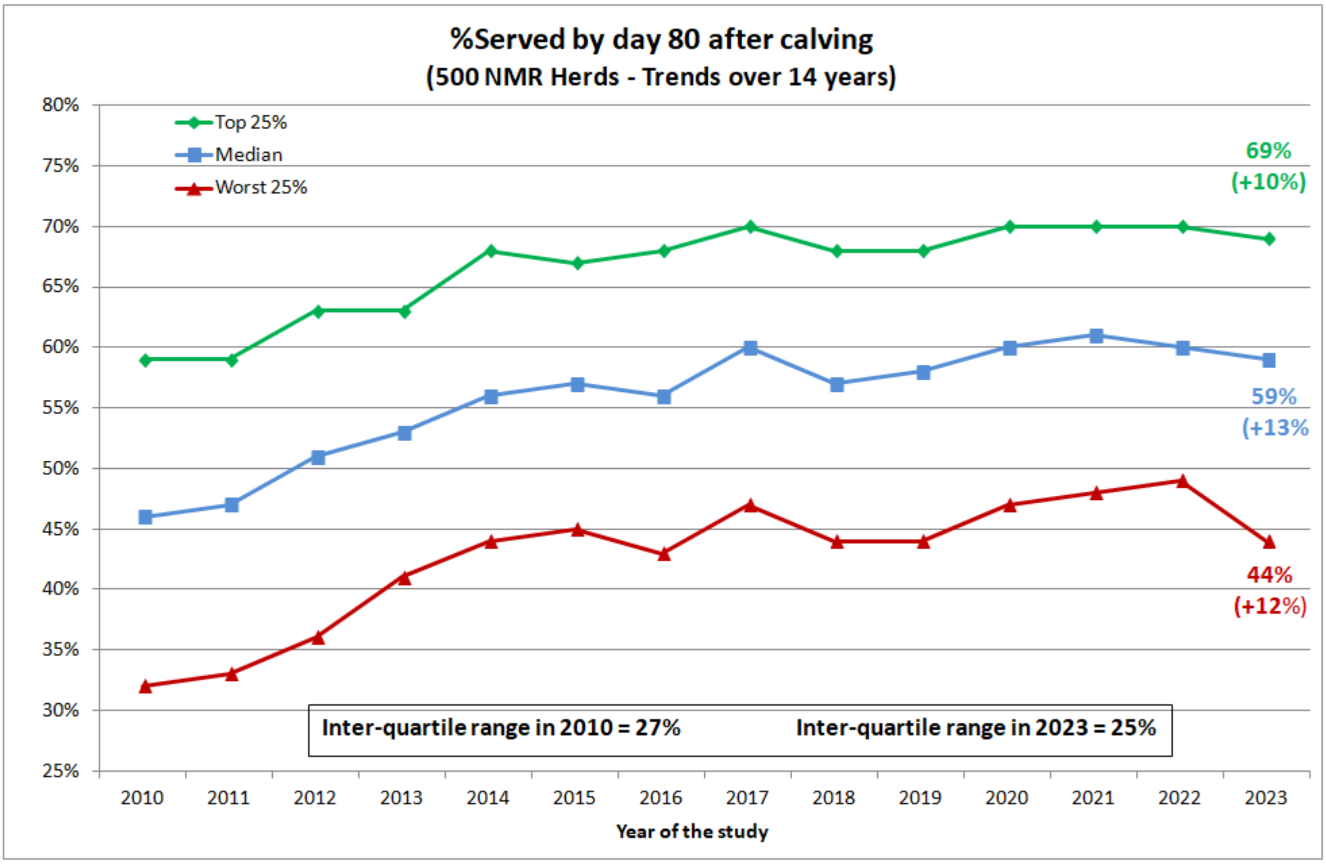


**Figure 9. % served by 80 days after calving.**

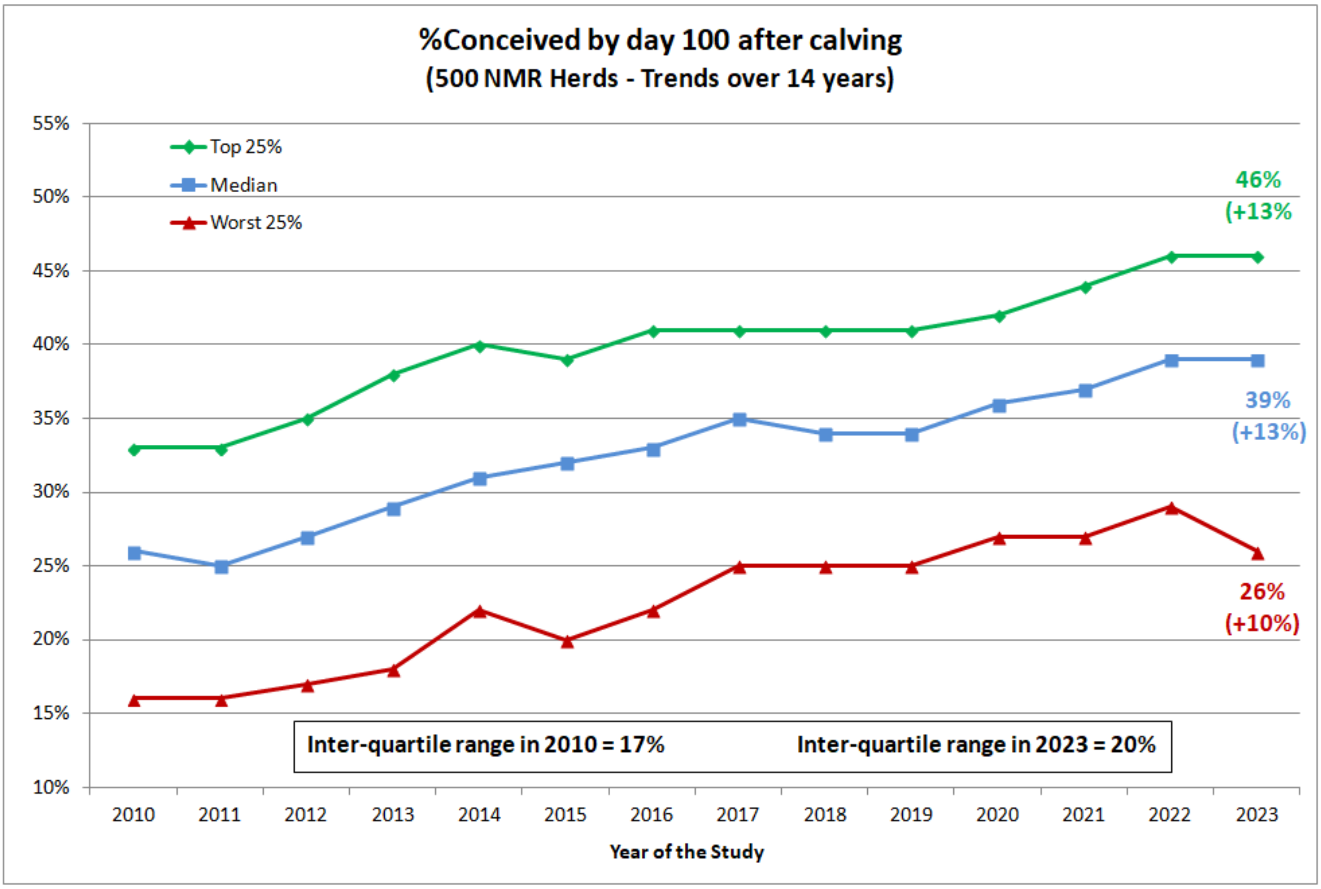


**Figure 10. % conceived 100 days after calving.**

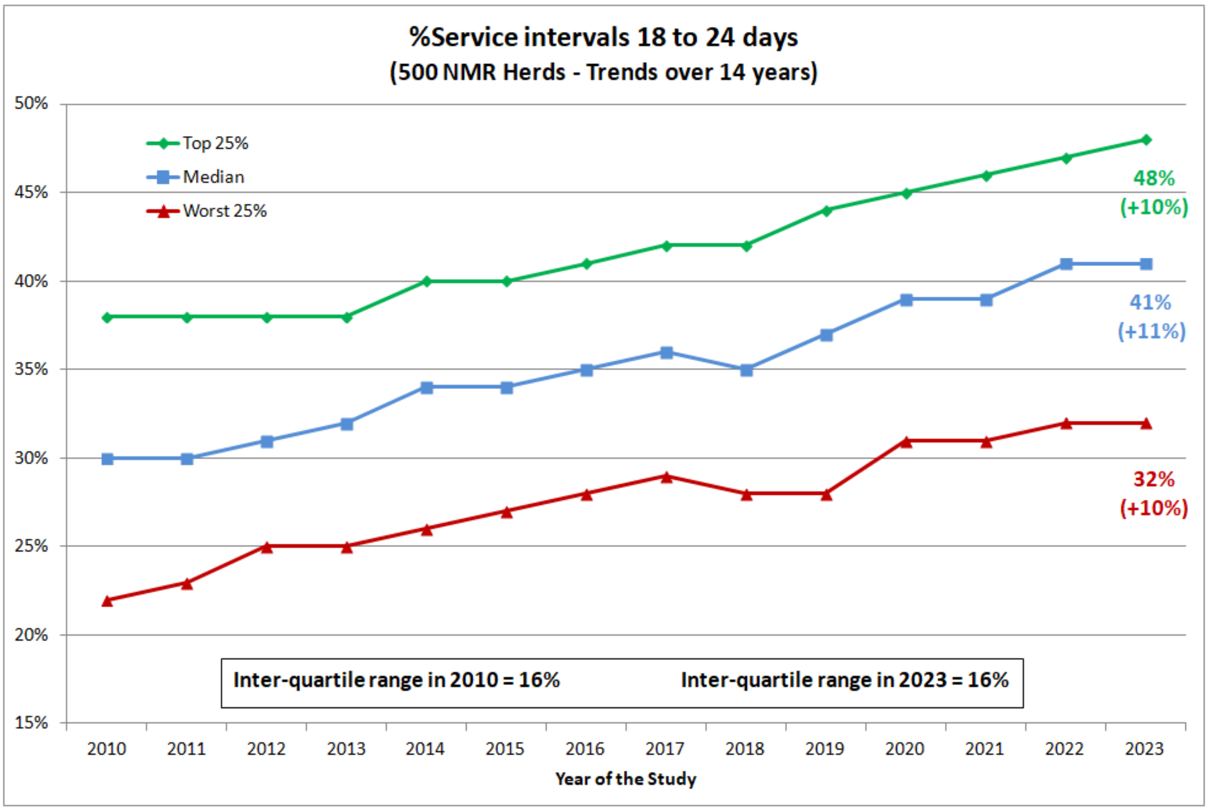


**Figure 11. Heat detection (%). The proportion of all repeat services that are 18-24 days after the previous service.**

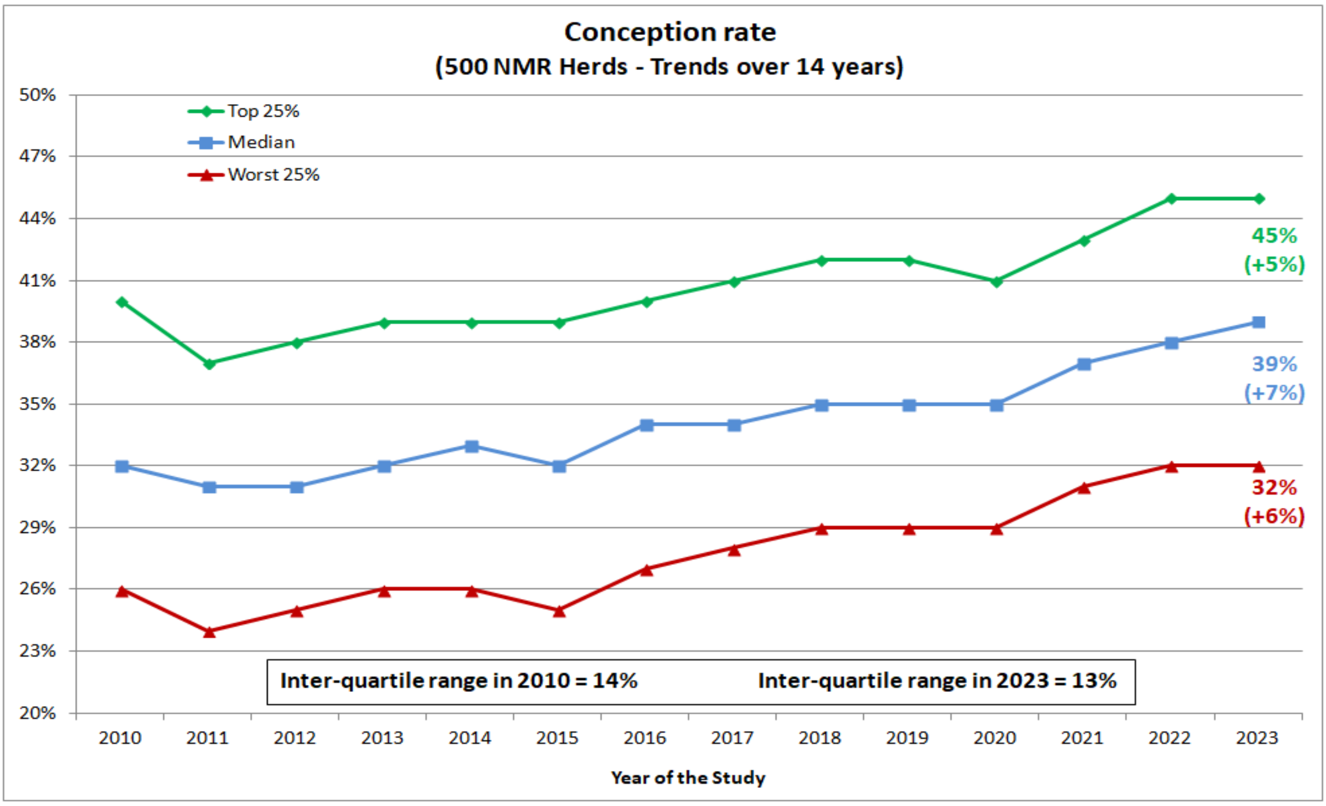


**Figure 12. Conception rate (%).**

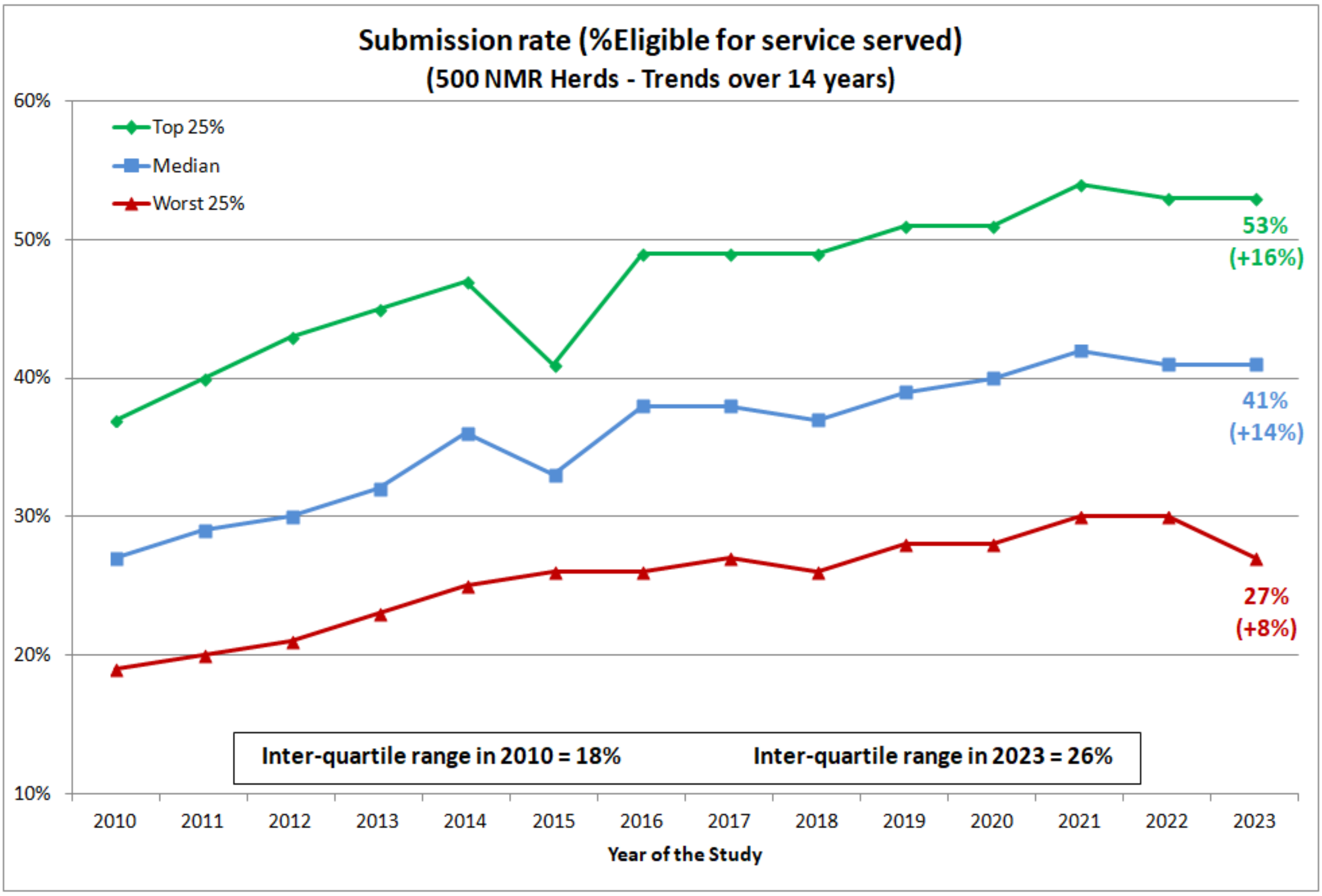


**Figure 13. Submission rate (%).**

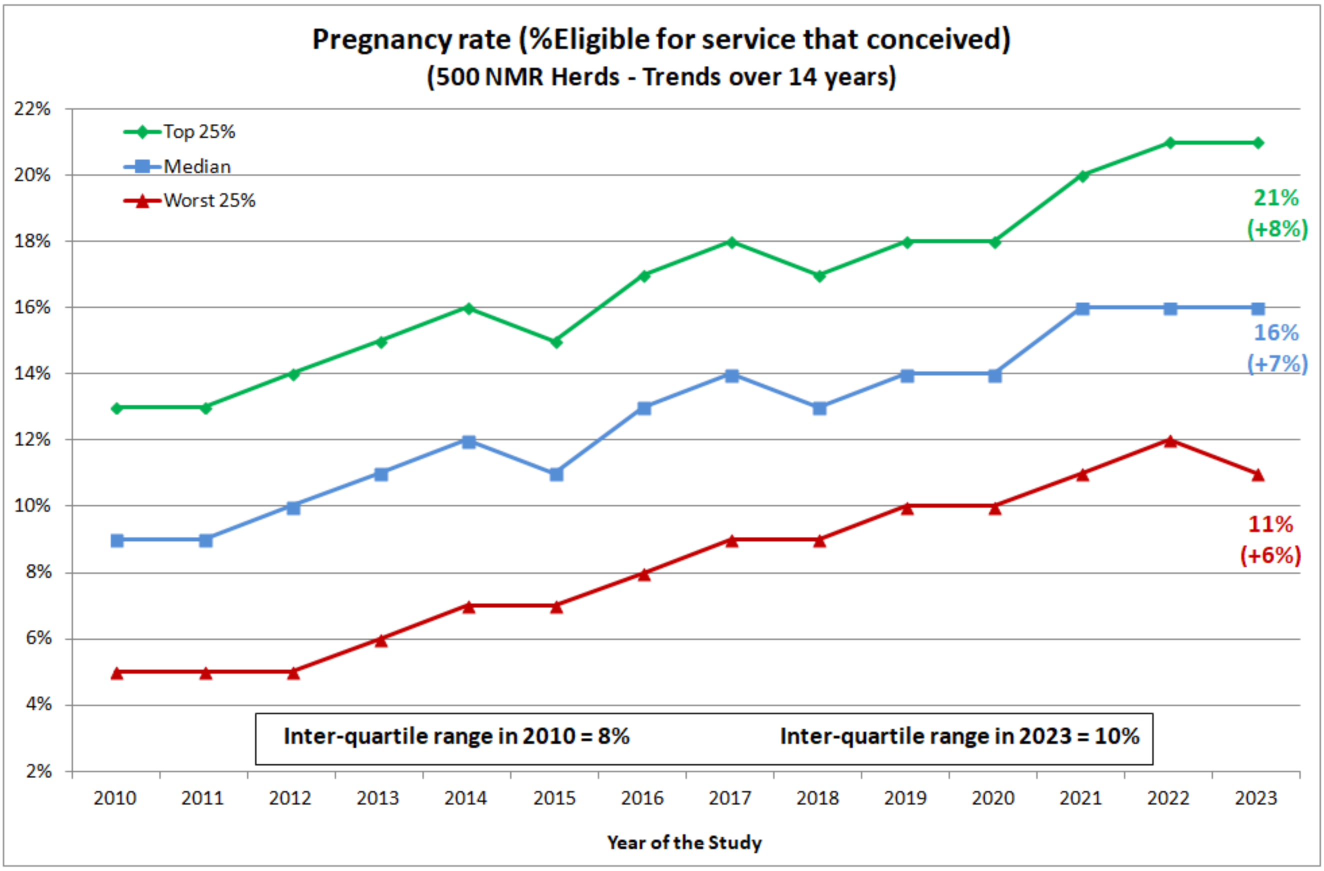


**Figure 14. Pregnancy rate (%).**

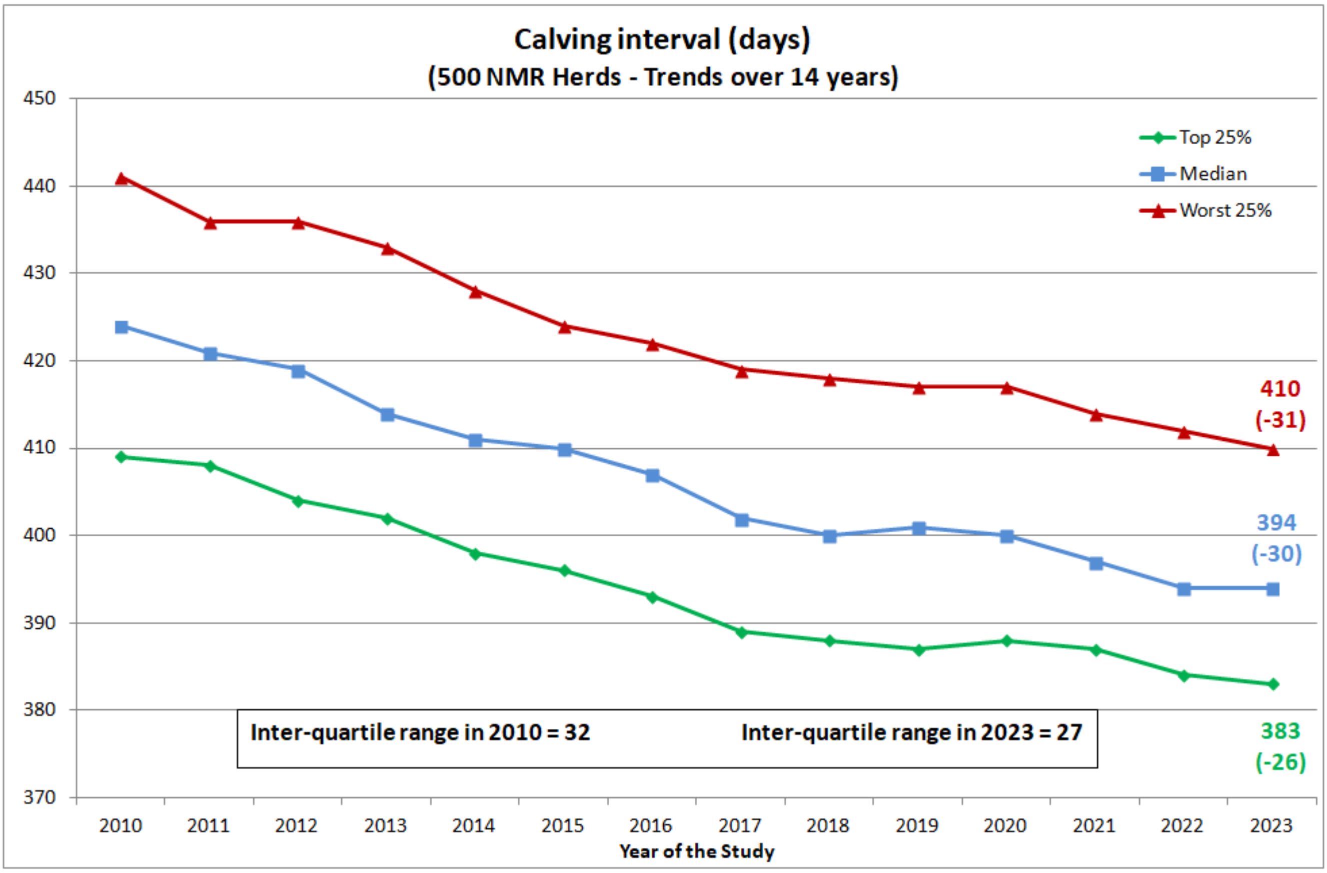


**Figure 15. Calving interval (days).**

## 3.5 Trends in milk production parameters since 2010.

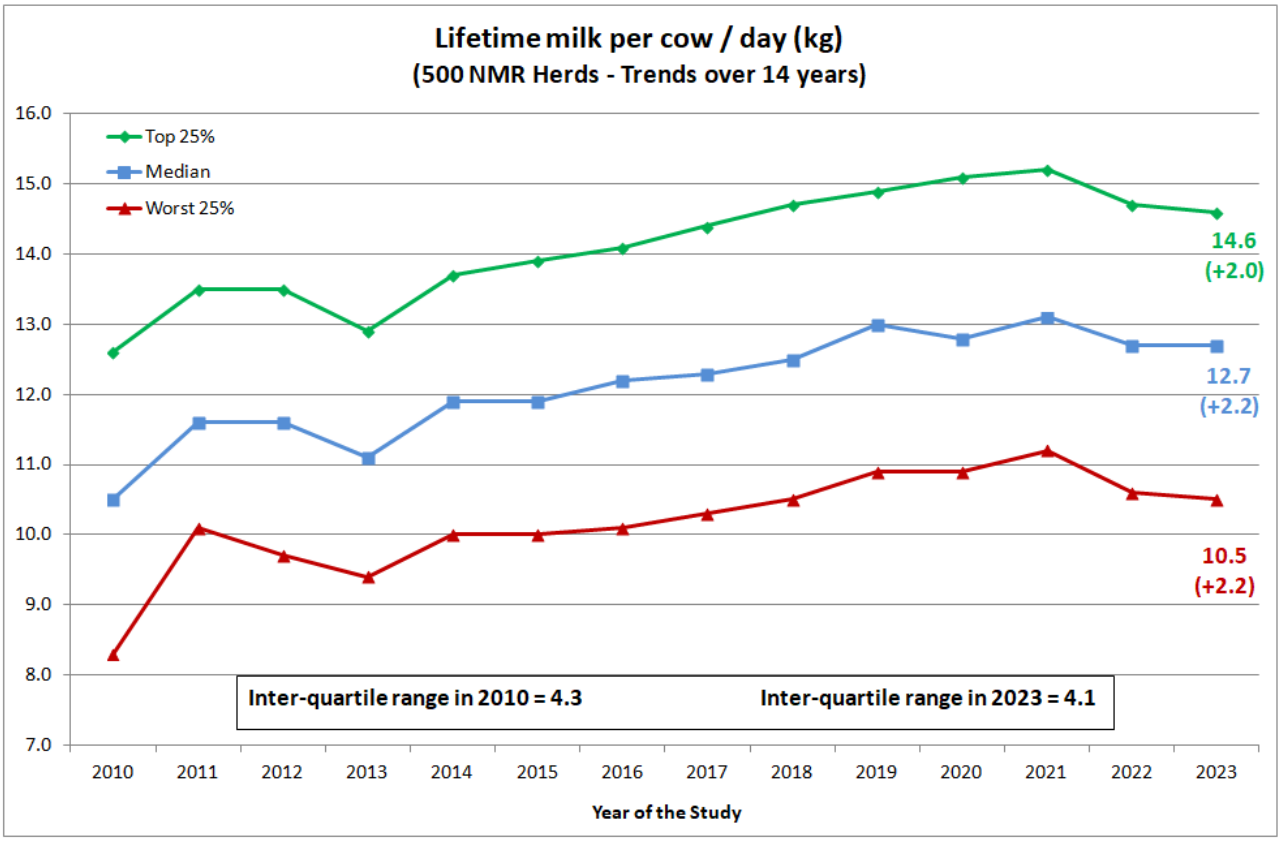


**Figure 16. Lifetime milk per cow / day (kg). This includes days between birth and first calving.**

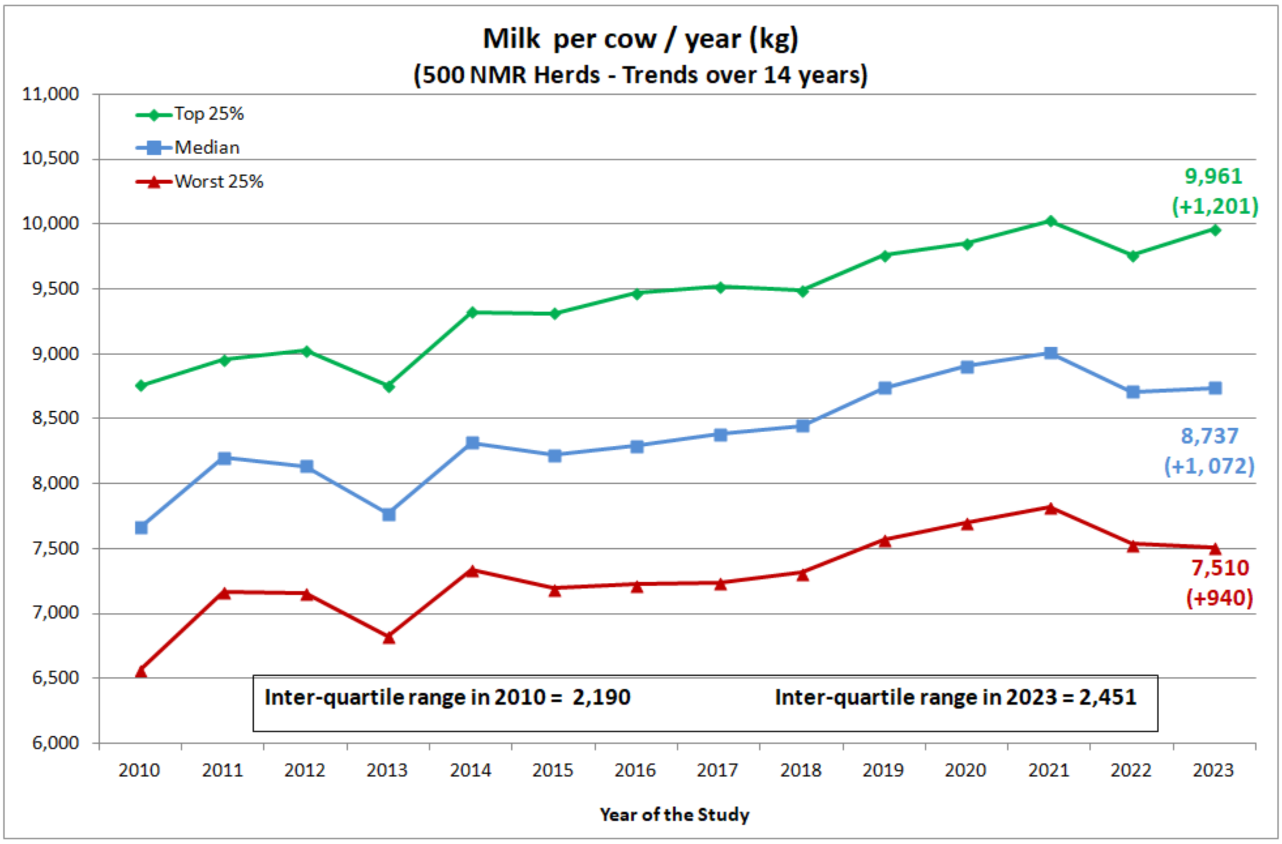


**Figure 17. Milk per cow / year (kg).**

## 3.6 Trends in SCC parameters and mastitis rate since 2010 (SCC) or 2016 (mastitis).

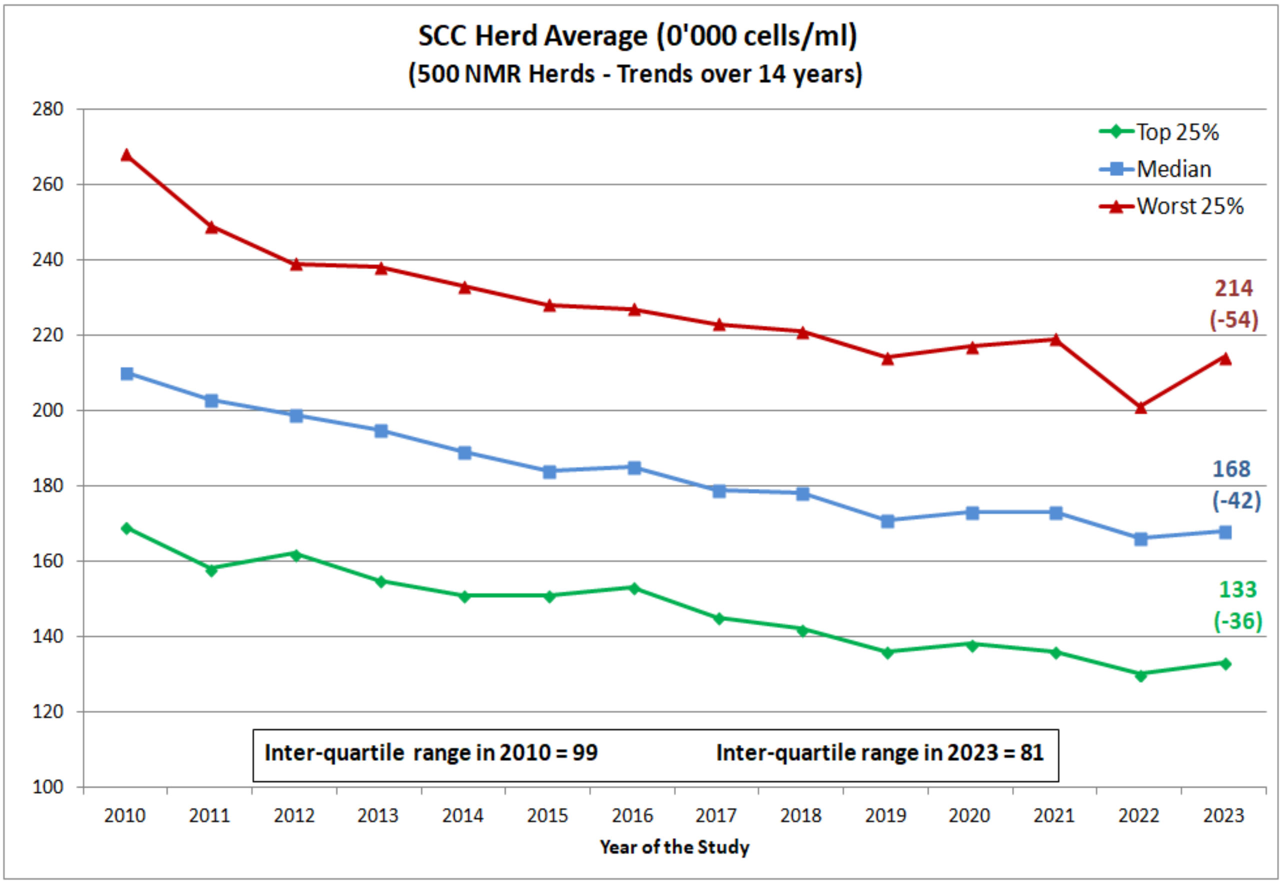


**Figure 18. Herd average SCC (0’000 cells/ml).**

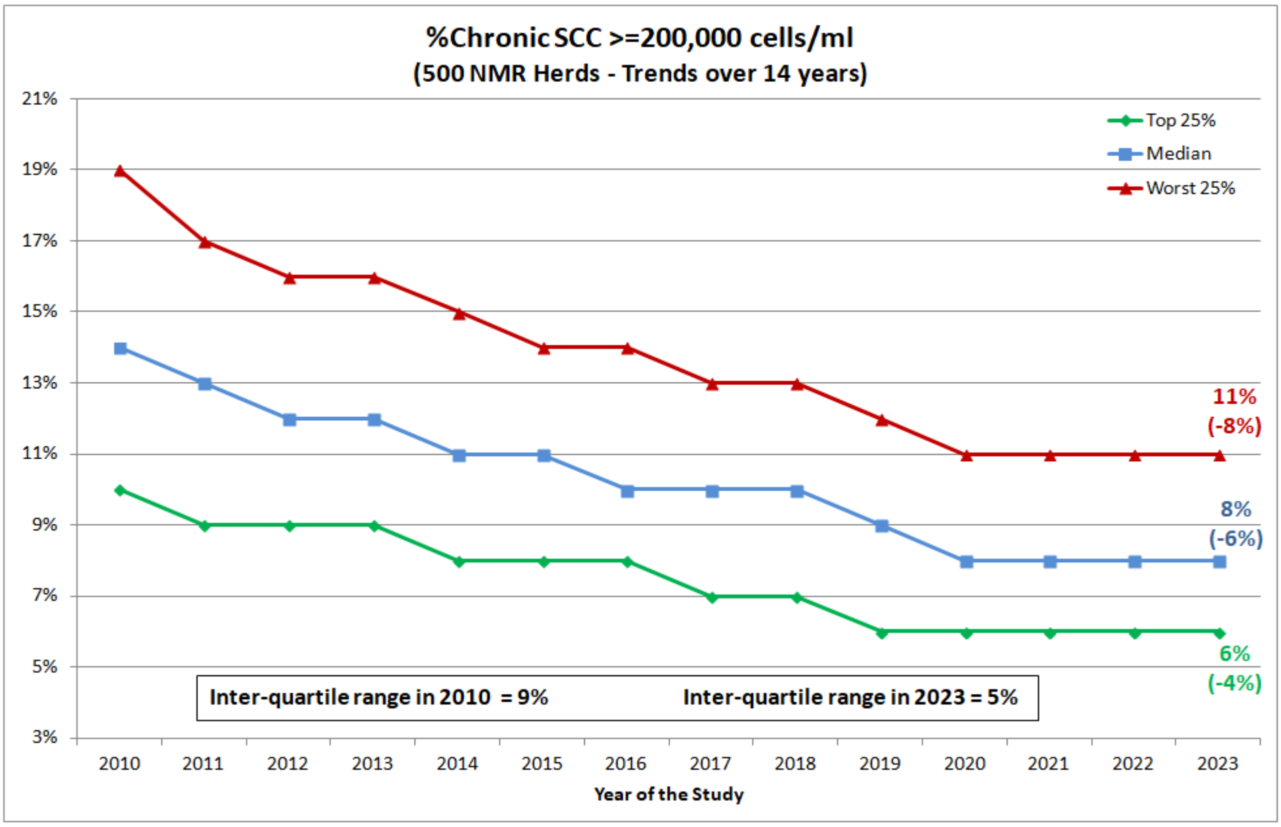


**Figure 19. % milk samples originating from chronic (repeat) high SCC cows.**

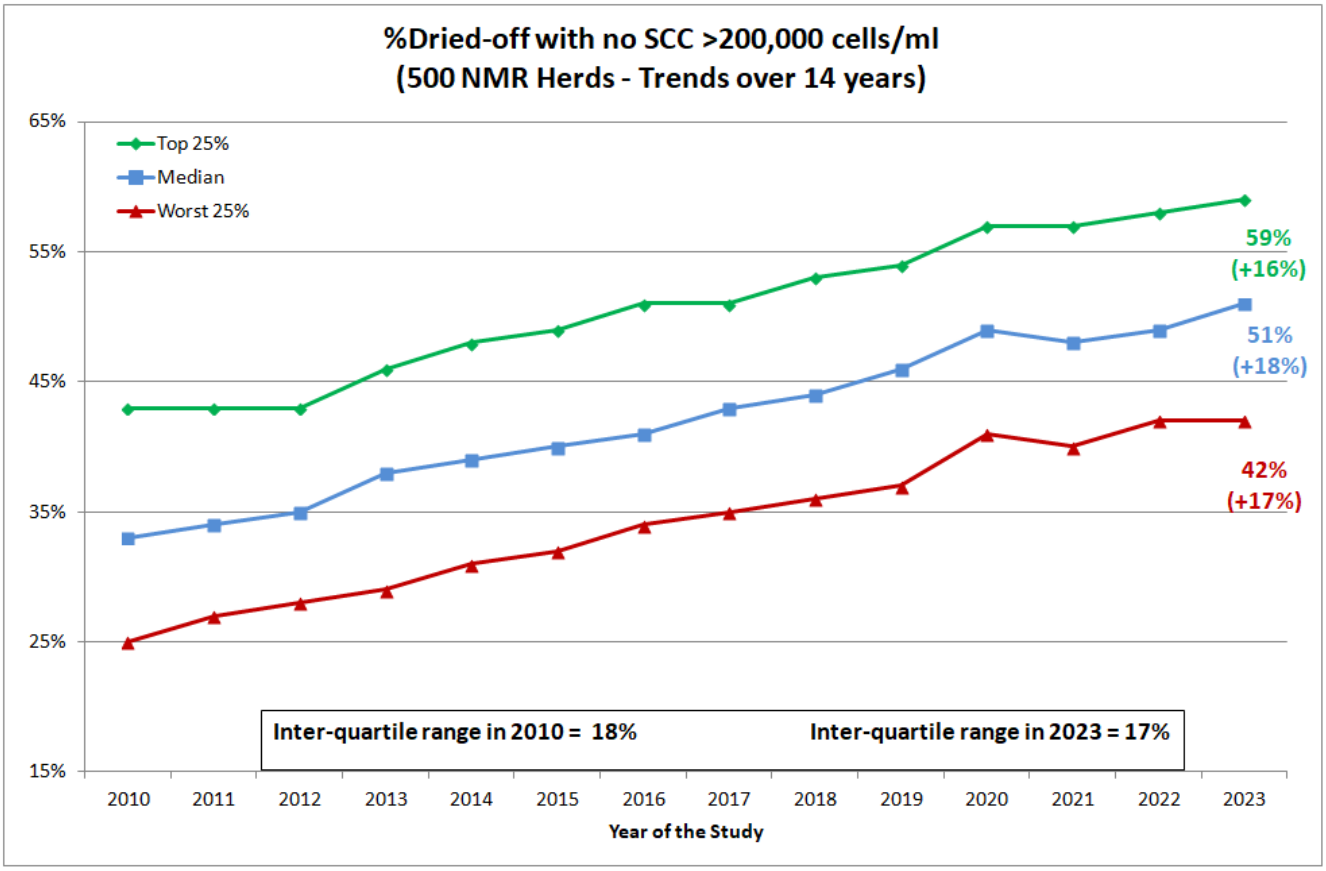


**Figure 20. % dried-off with no high SCC in lactation.**

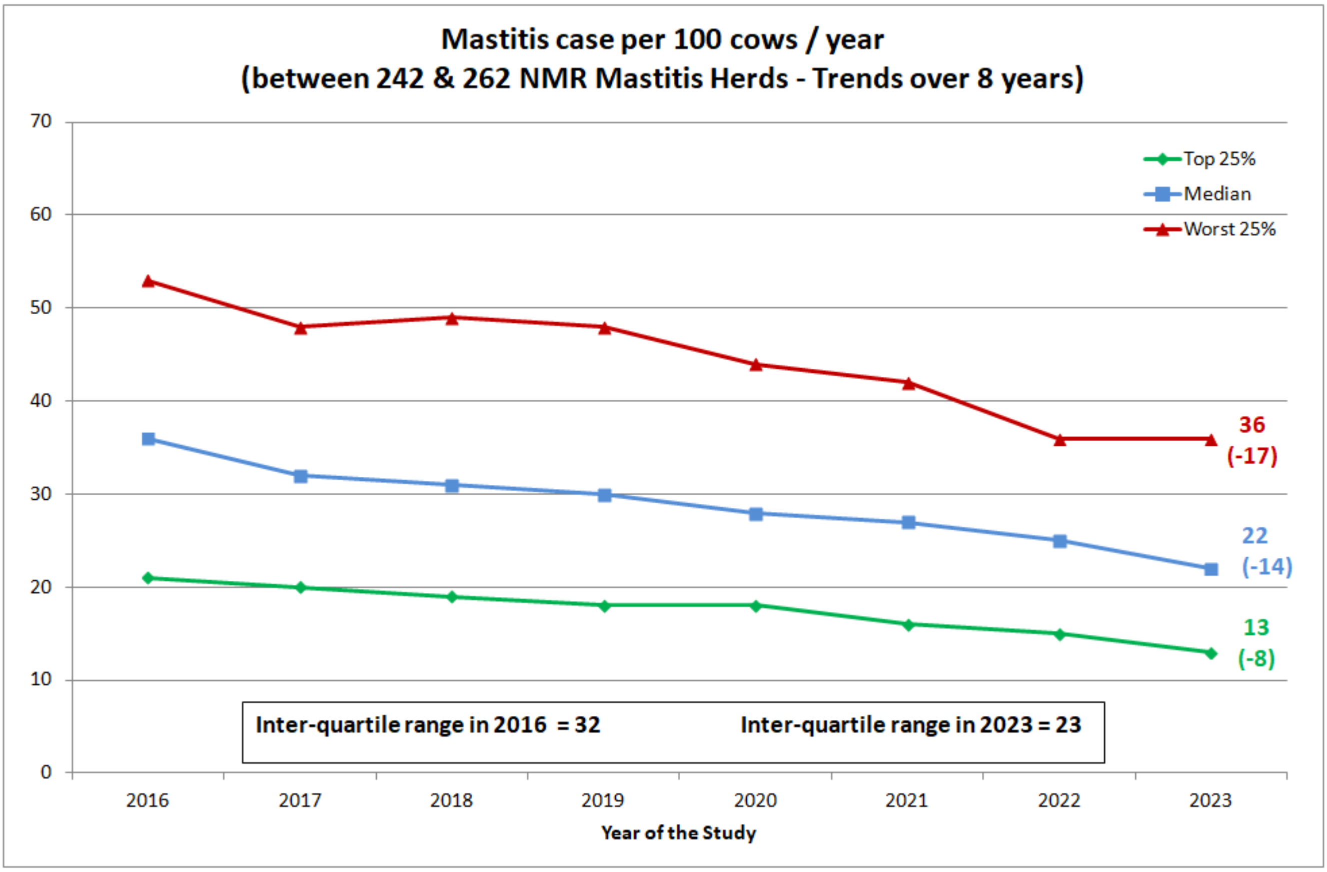


**Figure 21. Mastitis rate (cases / 100 cows in milk / year) – mastitis groups of herds since 2016.**

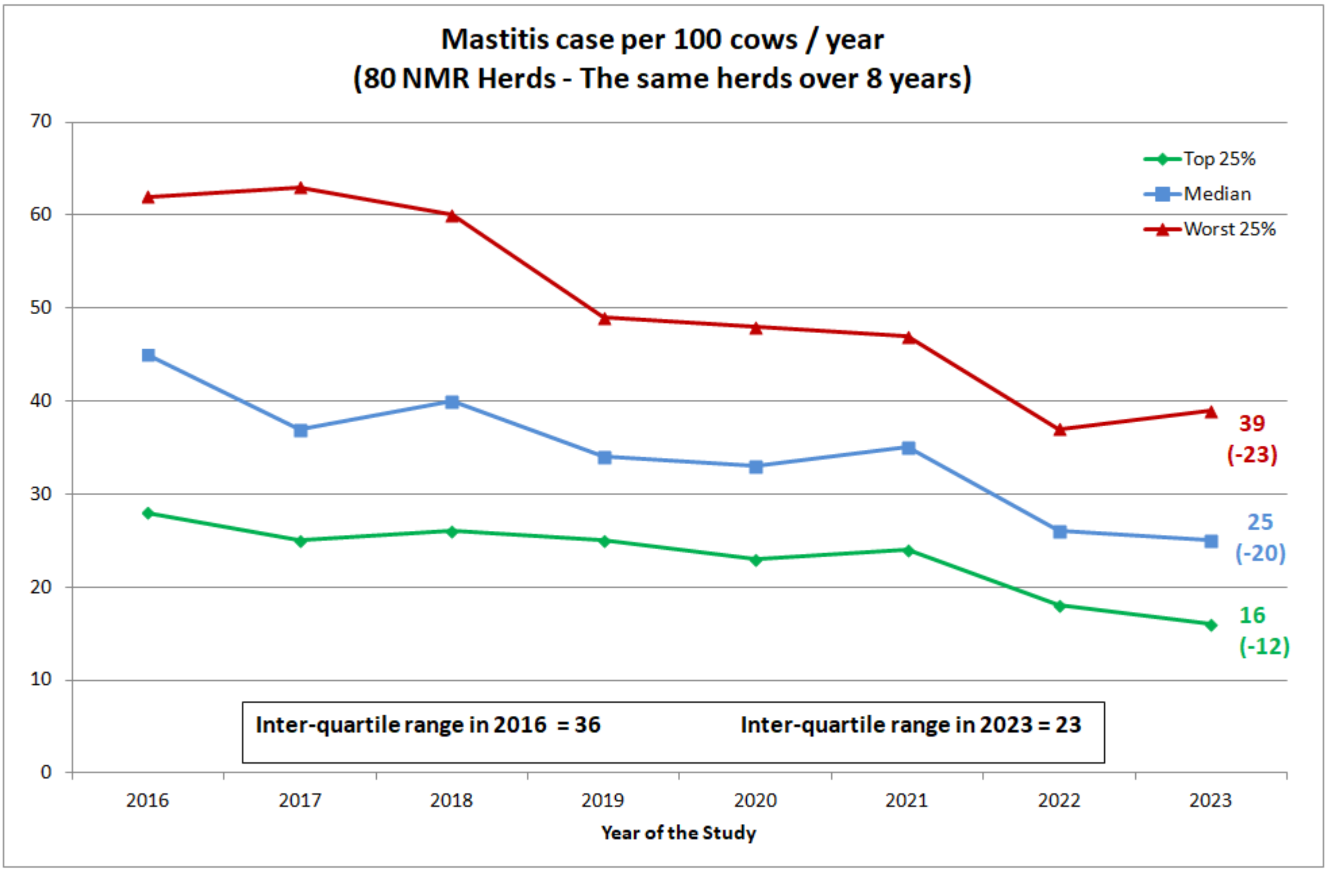


**Figure 22. Mastitis rate (cases / 100 cows in milk / year) – the same herds since 2016.**

# Section 4. The practical use of KPIs by farmers and their technical advisers

## 4.1 Interpreting the “KPIs at a glance” report in InterHerd+

The figures obtained from this study can be treated as “national standards” for UK 'black and white' dairy herds in 2023, with target values set at the level currently achieved or bettered on one in four of the 500 farms in the survey. A farmer can readily see where their herd would perform for each parameter relative to the 500 herds. This can be used to focus discussion on the causes and options/need for improvement.

The KPI report in the InterHerd+ program provides an overview of performance for an individual herd. Parameters are calculated in an identical way so are directly comparable to the KPI values in the study. Comparing the performance of the herd with the results of the study highlights areas of strength and weakness in that herd’s performance (Supplementary Figure 1).

The combination of parameters relating to production, fertility and health, emphasizes the dynamic nature of dairy production and the need for high standards across all areas of herd management. Many herds are excellent in one area of production, fertility or health but seldom in all. There is always room for improvement in efficiency.

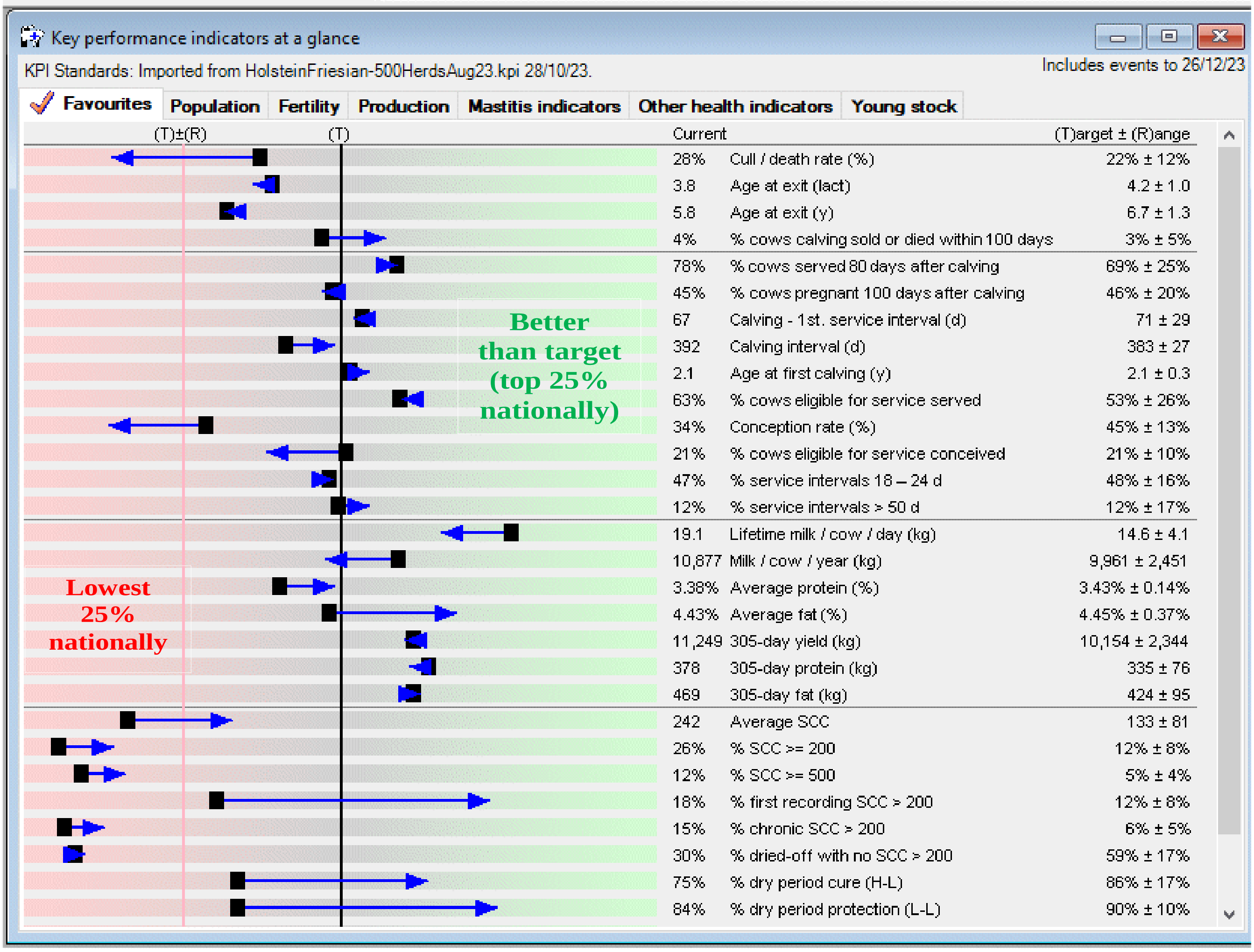


**Supplementary Figure 1. The “KPIs at a glance” report in InterHerd+.**

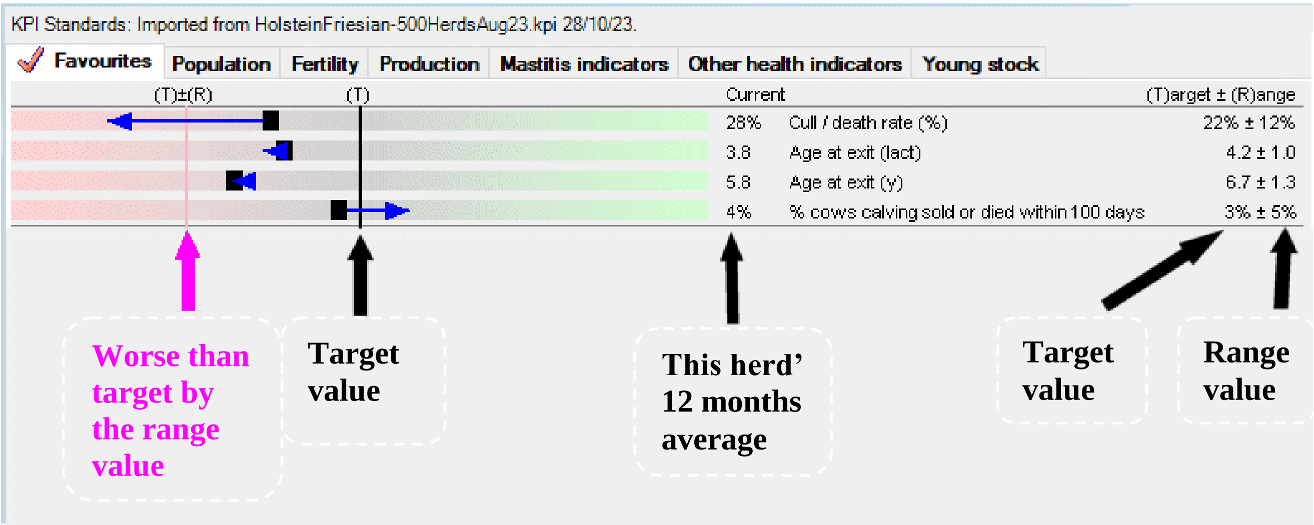


**Supplementary Figure 2. Interpreting the "KPIs at a glance" report in InterHerd+.**

The value displayed to the left of each parameter title represents the herd's performance over the last year. It is the rolling 12 month average for that parameter. In Supplementary Figure 2 the herd had a cull/death rate averaging 28% over the previous 12 months.

To the right of each listed parameter is a **target** value and a **range** (corresponding to the values given in Table KPIs A and Table KPIs B. In Supplementary Figure 2 above the TARGET value for cull/death rate is 22% with a range of ±12%.

These values are also displayed graphically to the left of the parameter titles. The **target** value is represented by the **vertical black** line. The area to the right hand side is shaded green to denote a performance level that is **better** than the target value.

Left of the target line is shaded **red** denoting performance that is **worse** than the target value. The **vertical red** line represents the level that is "**worse than the target by the range value**" (so the performance of the poorer performing 25% of herds). In Supplementary Figure 2 for culling rate, the red vertical line represents the target (22%) worse by the range (12%) so a culling rate of 34%.

The positions of the black square and blue arrow 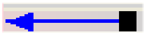 show how the current herd is performing for each parameter relative to the specified target and range values. The arrow indicates any direction of change.

- The **black square** is the **12 month rolling average** value for that parameter. So it is the longer-term performance based on the last 12 months of data (the value displayed to the left of the parameter title).
- The **blue** arrow head is the **3 months rolling average** value for that parameter. In other words it is the short-term performance based on the last 3 months only. The line and arrow show the difference and direction of change between the 3 and 12 month average values. Beware that while this may indicate a significant change in herd performance, the blue line may also be influenced by seasonal factors in that 3 month period.

## 4.2 Using the target and range values to highlight a herd's strengths and weaknesses

**Herd strengths:** This study identifies the level achieved by the best 25% of the herds for each parameter. That value is then set as the "TARGET" for comparison with other herds. In Supplementary Figure 3, any KPI with a black square to the **right (green side) of the vertical black target line** is "**the best 25%**" when compared to the 500 study herds. In Supplementary Figure 3 below, the herd displayed has 10 parameters that are "better than target" so this herd would currently be in the top 25% of herds nationally for those parameters. This includes 5 fertility parameters and 5 yield parameters.

**Herd weaknesses:** The **vertical red line** represents the performance achieved or bettered by 75% of the 500 herds (the target, worse by the range). Any parameter with a black square to the **left of the vertical red line** would be **"in the bottom 25%"** for that parameter when compared to the 500 study herds. There are 5 SCC parameters in Supplementary Figure 3.

**Average performance levels:** Parameters that fall between the vertical black and red lines are within the inter-quartile range (so the middle 50% of herds) when compared with the 500 study herds. Highlighting the strengths and weaknesses of a dairy herd.

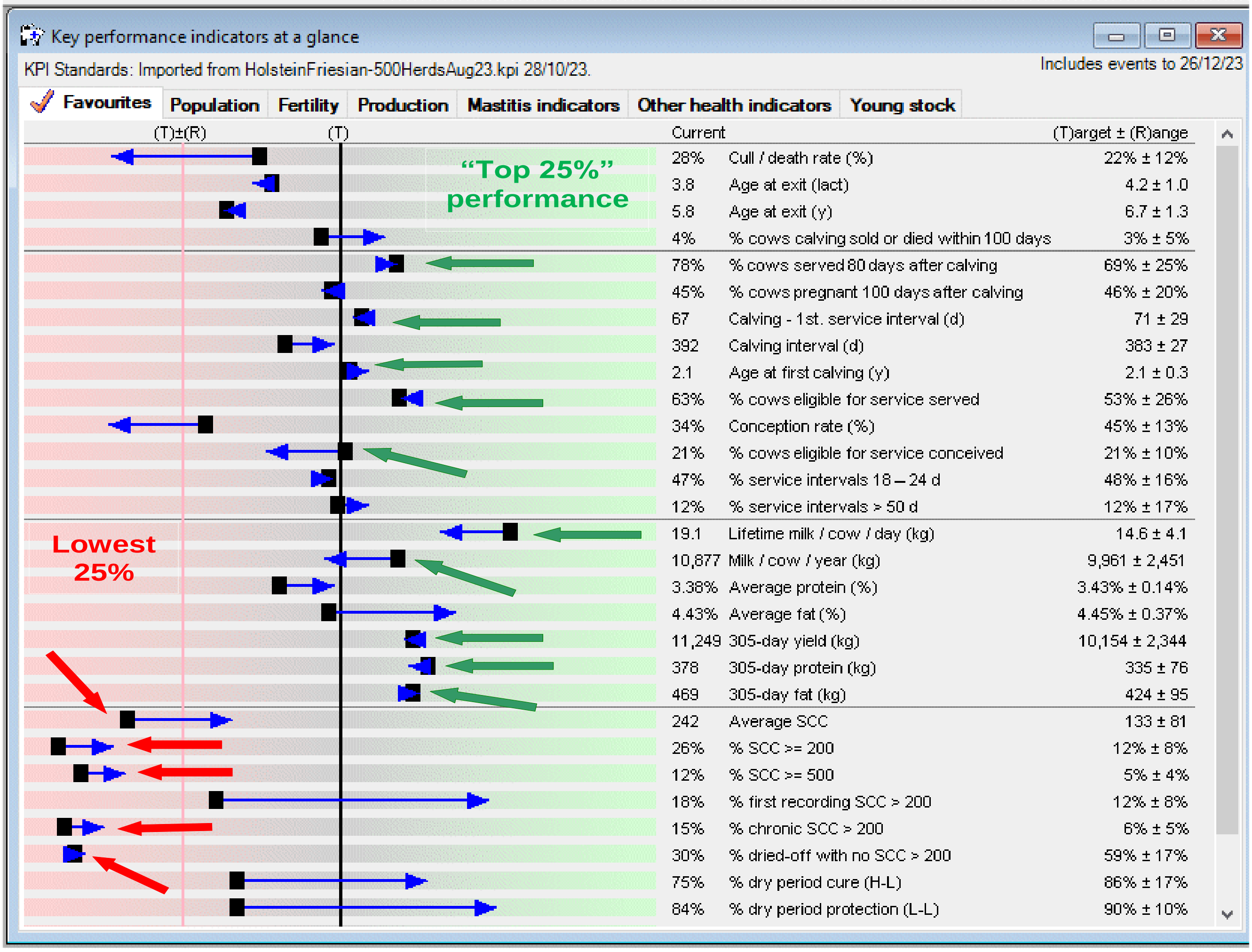


**Supplementary Figure 3. Highlighting the strengths and weaknesses of a dairy herd.**

Supplementary Figure 3 must be treated as a **DISCUSSION DOCUMENT**. The emphasis is on achieving an appropriate balance of performance in production, fertility and health. A parameter in the top 25% is not necessarily a good thing. The aim is to **stimulate informed discussion between farmers and their advisers** about **what** is happening **and WHY.**

## 4.3 Correlation between Herd SCC vs % chronic high SCC milk samples

The 500 herd studies also provide data to investigate correlations between different KPIs which can provide simple messages for farmers and advisers. A good example of this is the strong correlation between the Herd SCC and the percentage of milk samples deriving from chronic high SCC cows ($2^{nd}/3^{rd}/4^{th}$ etc consecutive high SCC milk sample). Supplementary Figure 4 shows the distribution of the 500 herds from the current study (year ending $31^{st}$ August 2023).

Each blue square represents one of the 500 study herds. The very strong correlation ($R^2$=0.745) is clearly evident giving a very clear message. If you have a high level of chronic cows in your herd you are also very likely to have a high herd SCC with all the costs and penalties associated with that.

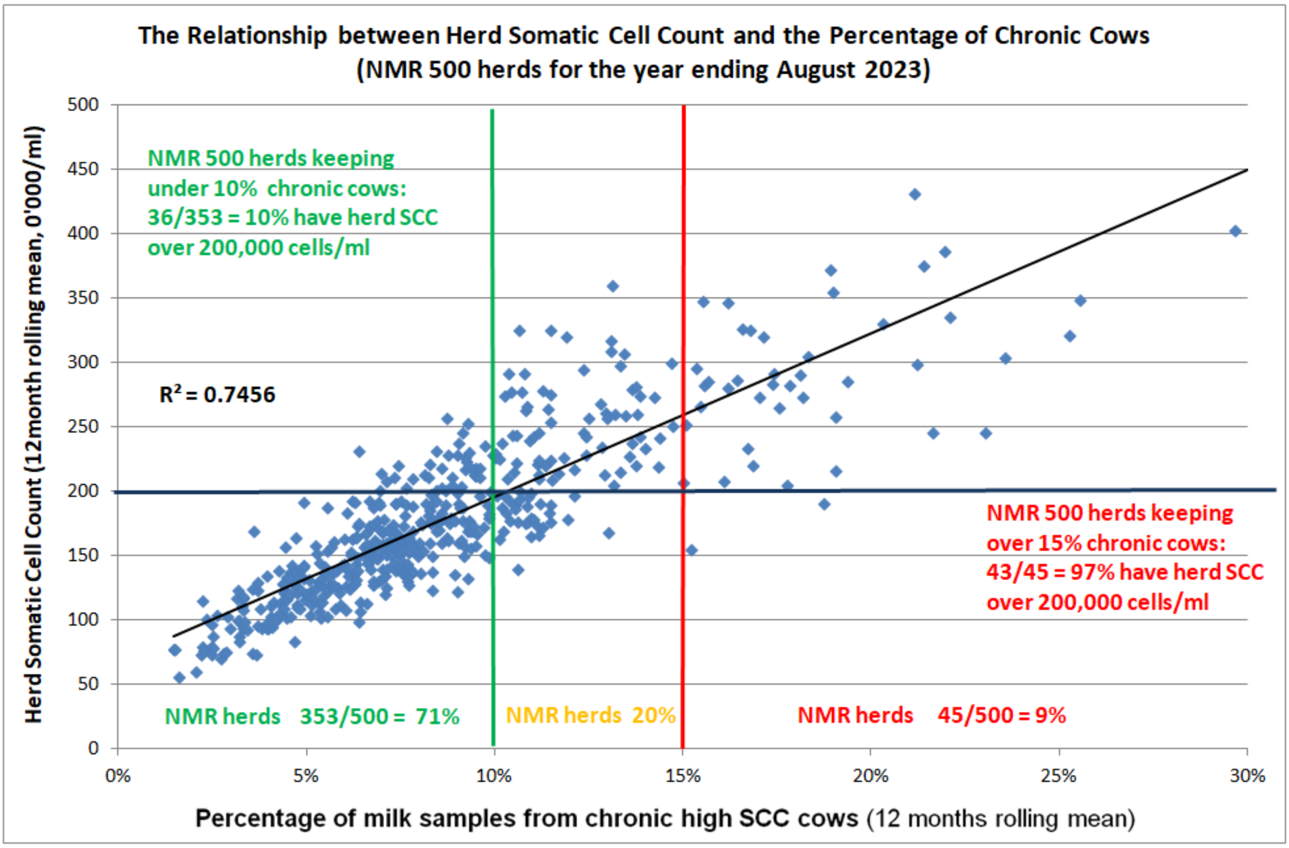


**Supplementary Figure 4. The relationship between herd average SCC and the % chronic high SCC cows.**

In addition to the correlation the graph shows that in herds where over 15% of the milk is from chronic cows the vast majority are penalised for having a high herd SCC. In 2023 there were 43 herds of this type and 97% of them had a high herd SCC (averaging greater than 200,000 cells/ml). In contrast, there were 353 herds with less than 10% chronic cows of which only 36 (10%) also had a high herd SCC.

This strong correlation has been evident in all the 14 annual KPI studies. Supplementary Table 1 shows the enormous gains that the UK dairy industry has made in tackling high herd SCCs. The 71% of the herds (353/500) in the most recent study that qualify as “Low levels of chronic cows” (less than 10% chronic cows), are a dramatic improvement on the 24% of herds in the original study in 2010. Over the same time interval the % of herds keeping high levels of chronic cows (≥15% of the herd) has dropped from 41% of herds in 2010 to 9% in 2023.

**Supplementary Table 1. Percentage of herds with low (<10%) and high (≥15%) levels of chronic high SCC samples, in the KPI studies from 2010 to 2023.**

| KPI study year | Low levels of chronic cows (<10% of milking cows) | High levels of chronic cows (≥15% of milking cows) |
|---|---|---|
| 2010 | 24% of herds | 41% of herds |
| 2011 | 32% of herds | 35% of herds |
| 2012 | 34% of herds | 30% of herds |
| 2013 | 36% of herds | 29% of herds |
| 2014 | 42% of herds | 24% of herds |
| 2015 | 44% of herds | 21% of herds |
| 2016 | 51% of herds | 18% of herds |
| 2017 | 51% of herds | 17% of herds |
| 2018 | 56% of herds | 12% of herds |
| 2019 | 62% of herds | 13% of herds |
| 2020 | 65% of herds | 10% of herds |
| 2021 | 67% of herds | 10% of herds |
| 2022 | 71% of herds | 8% of herds |
| 2023 | 71% of herds | 9% of herds |

In contrast to the strong correlation between herd SCC and the level of chronic cows, there is no clear correlation between the level of mastitis incidence and the herd SCC. Supplementary Figure 5 shows the poor correlation observed in 2023. Similar poor correlation was observed in previous studies.

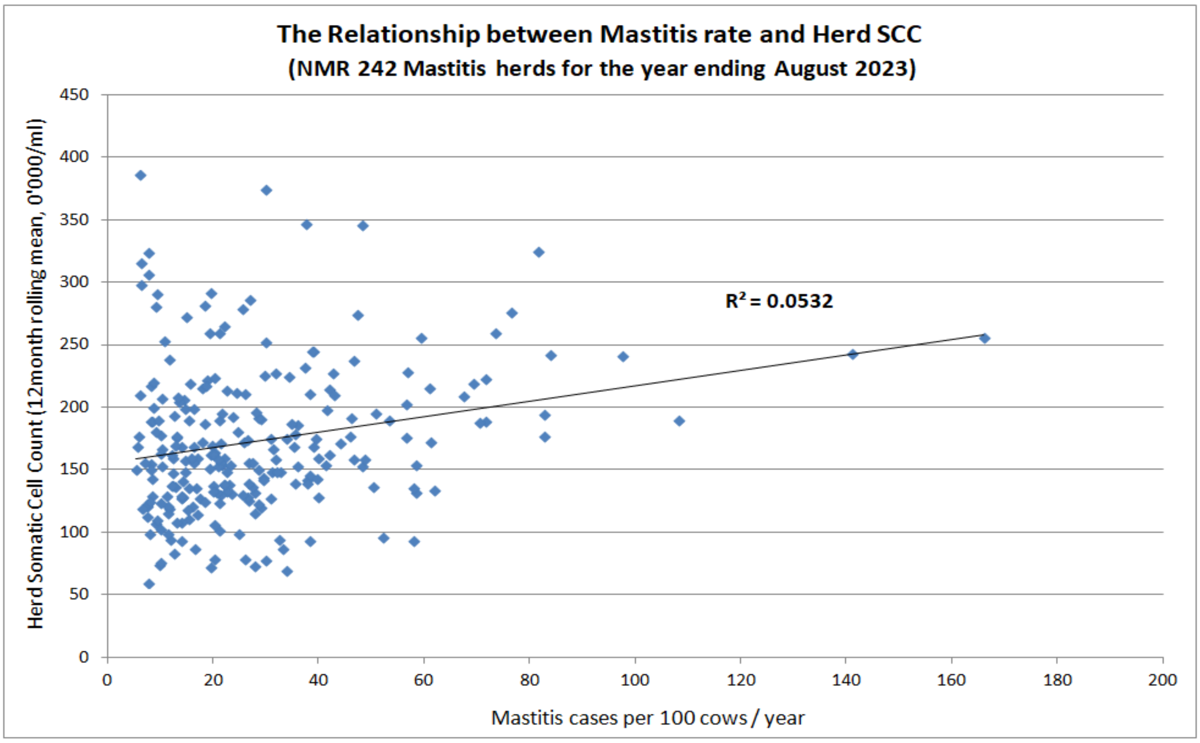


**Supplementary Figure 5. The relationship between herd average SCC and mastitis incidence.**

## Conclusion

Since 2010, key performance indicators relating to fertility, milk production and somatic cell count have improved. Age at first calving has decreased from 2.4 years to 2.2 years, % served by 80 DPP increased from 46% to 59%, conception rates have increased from 32% to 39% and calving interval has gradually decreased from 424 days to 394 days. Lifetime milk/cow/day increased by 25% from 10.5kg in 2010 to 12.7kg in 2023. Milk protein has increased from 3.27% to 3.36%. Milk fat has increased from 3.96% to 4.26%. Average herd SCC decreased by 20% from 210,000 cells/ml to 168,000 cells/ml. The proportion of cows with chronic (repeat) SCC >=200,000 cells/ml has decreased from 14% to 8%. However, the demographic of UK dairy herds have noticeably changed. The productive life of dairy cows has decreased from 1,511 days to 1,367 days. Age at exit has decreased from 6.6 to 6.0 years and cull rates have increased from 24% to 28%.Therefore, herd sizes have increased and older cattle are more readily replaced for replacement heifers, although the motivations behind reduced longevity remains unclear.

# Appendix 1. Changes in KPIs between 2010 and 2023

Since the initial study in 2010, wherever possible the same herds are kept in the sample used each year. If necessary, herds with poorly recorded fertility data and herds no longer recording were replaced with randomly selected new herds. Within the sample of the 2023 study, there remain 100 herds (20%) that have been included in all the 14 studies (2010 to 2023).

As a check to confirm that replacement of some herds and retention of others has not introduced any unexpected bias in the sample, Table Appendix 1 shows the changes observed in KPIs over 14 years (2010 vs. 2023) for the ‘changing sample’ of 500 each year alongside the changes seen in the 100 herds common to all 14 years. Table Appendix 1 shows similar trends in the ‘common 100’ herds to the trends in the ‘whole 500’ herd samples. Overall, these results do not suggest that significant bias has been introduced by the year-on-year partial replacement of herds in the sample.

**Table Appendix 1 A comparison of the 2010 and 2013 median values within the 500-herds and 100 common herds.**

| | 500 herds groups | | 100 common herds | |
|---|---|---|---|---|
| | Median value | | Median value | |
| **Parameter** ***Year of the study*** | **2010** | **2023** | **2010** | **2023** |
| A. Culling rate | 24% | 28% | 23% | 28% |
| B. Culling / death rate in first 100 days of lactation | 7% | 5% | 6% | 5% |
| C(a). Age at exit (years) | 6.6 | 6.0 | 6.7 | 5.9 |
| C(b). Age at exit (days) | 2,393 | 2,179 | 2,453 | 2,166 |
| D. Age at exit by Lactations | 3.9 | 3.6 | 4.0 | 3.6 |
| E. Percentage Served by day 80 | 46% | 59% | 46% | 59% |
| F. Percentage conceived 100 days after calving | 26% | 39% | 26% | 38% |
| G. Calving to 1st service interval (days) | 105 | 81 | 102 | 82 |
| H. Calving interval (days) | 424 | 394 | 422 | 394 |
| I(a). Age at 1st calving (years) | 2.4 | 2.2 | 2.5 | 2.2 |
| I(b). Age at 1st calving (days) | 893 | 804 | 912 | 795 |
| J. Conception rate | 32% | 39% | 32% | 38% |
| K. Percentage service intervals at 18-24 days | 30% | 41% | 32% | 41% |
| L. Percentage service intervals >50 days | 32% | 19% | 30% | 19% |
| M. Percentage eligible for service that served | 27% | 41% | 28% | 40% |
| N. Percentage eligible for service that conceived | 9% | 16% | 10% | 17% |
| O. Lifetime milk / cow / day (kg) | 10.5 | 12.7 | 10.6 | 12.5 |
| P. Milk / cow / year (kg) | 7,665 | 8,737 | 8,140 | 8,567 |
| Q. Average Protein% | 3.27% | 3.36% | 3.25% | 3.35% |
| R. Average Fat% | 3.96% | 4.26% | 3.95% | 4.28% |
| V. Average SCC ('000 cells/ml) | 210 | 168 | 194 | 170 |
| W. Percentage SCC >=200,000 cells/ml | 24% | 15% | 22% | 16% |
| X. Percentage SCC >500,000 cells/ml | 9% | 7% | 8% | 6% |
| Y. Percentage 1st recording SCC >=200,000 cells/ml | 20% | 15% | 18% | 14% |
| Z. Percentage chronic SCC >=200,000 cells/ml | 14% | 8% | 12% | 7% |
| ZA. Percentage Dry period cure (High:Low) | 74% | 77% | 76% | 78% |
| ZB. Percentage Dry period protection (Low:Low) | 84% | 86% | 85% | 86% |
| ZC. Percentage Low SCC at end of previous lactation | 60% | 79% | 66% | 78% |

# Appendix 2. KPI definitions

In the following definitions the average population of cows is calculated using animal days. Every day that animal is present in the population at risk during the period of study is a 365$^{th}$ of an animal year. The total animal days is divided by 365 to give animal years, which equates to the average population at risk.

**Table Appendix 2 KPI definitions.**

| Parameter | Description |
|---|---|
| A. Culling rate | The number of cows dying or culled during the 12 month period expressed as a percentage of the average cow population for the same 12 month period. |
| B. Percentage culled / died 100 days after calving | The percentage of heifers/cows calving during the 12 month period that exit within 100 days after calving. |
| C. Age at exit (years) | The average age (in days) of cows culled/died in the analysis period, divided by 365.24 |
| D. Age at exit by lactations | The average number of lactations completed by cows culled/died in the analysis period. |
| E. Percentage Served by day 80 | The percentage of cows reaching the 80$^{th}$ day after calving that have been served at least once. |
| F. Percentage conceived 100 days after calving | The percentage of cows reaching 100 days after calving that have conceived. |
| G. Calving to 1$^{st}$ service interval (days) | The average days between calving and 1$^{st}$ service for all cows served for the first time in a lactation during the analysis period. |
| H. Calving interval (days) | The interval between calvings, in days, for all re-calvings recorded in the analysis period. |
| I. Age at 1$^{st}$ calving (years) | The age at first calving for all cows calving for the first time during the analysis period. |
| J. Conception rate | The number of conceptions as a percentage of the total number of services (services to cows culled are included) during the analysis period. |
| K. Percentage service intervals at 18-24 days (Heat detection) | The percentage of all service intervals for cows returning to service during the analysis period that are between 18 and 24 days (equating to one oestrous cycle after the previous service). |
| L. Percentage service intervals >50 days | The percentage of all service intervals for cows returning to service during the analysis period that are over 50 days. |
| M. Percentage of cows eligible for service that were served (Submission rate) | The percentage of cows that are eligible for service (42 days+ after calving and not barren or already pregnant) during the analysis period that are served per 21 day (oestrous cycle) period. |
| N. Percentage of cows eligible for service that conceived (Pregnancy rate) | The percentage of cows that are eligible for service (42 days+ after calving and not barren or already pregnant) during the analysis period that conceive per 21 day (oestrous cycle) period. |
| O. Lifetime milk / cow/day (kg) | The average of total milk yield divided by age in days (from birth to culling) for cows leaving the herd during the analysis period. |
| P. Milk / cow / year (kg) | The total milk produced per cow place in the year. The total milk divided by the average population of cows (both in milk and dry). |

| Parameter | Description |
|---|---|
| Q. Average protein% | The weighted average protein% of all milk recorded during the analysis period. |
| R. Average fat% | The weighted average fat% of all milk recorded during the analysis period. |
| S. 305 day yield (kg) | The average 305 day production for all cows reaching 305 days after calving during the analysis period. |
| T. 305 day protein (kg) | The average 305 day production of milk protein for all cows reaching 305 days after calving during the analysis period. |
| U. 305 day fat (kg) | The average 305 day production of milk fat for all cows reaching 305 days after calving during the analysis period. |
| V. Average SCC ('000 cells/ml) | The weighted average somatic cell count of all milk recorded during the analysis period. |
| W. Percentage SCC ≥200,000 cells/ml | The percentage of all recorded milk samples during the analysis period that had an individual SCC reading of 200,000 cells/ml or higher. |
| X. Percentage SCC ≥500,000 cells/ml | The percentage of all recorded milk samples during the analysis period that had an individual SCC reading of 500,000 cells/ml or higher. |
| Y. Percentage 1st recording SCC ≥200,000 cells/ml | The percentage of all cows starting new lactations that had a high SCC (≥200,000 cells/ml) reading at the first milk recording in the lactation. |
| Z. Percentage chronic SCC ≥200,000 cells/ml | The percentage of all milk samples taken in the analysis period that originated from chronic SCC cows where the current and previous milk samples both had SCC levels of 200,000 cells/ml milk or greater. |
| ZA. Percentage Dry period cure (High:Low) | Of re-calving cows recorded starting a new lactation during the analysis period: the percentage of cows ending the previous lactation with a HIGH SCC (≥200,000 cells/ml) that started the new lactation with a LOW SCC (<200,000 cells/ml). |
| ZB. Percentage Dry period protection (Low:Low) | Of re-calving cows recorded starting a new lactation during the analysis period: the percentage of cows ending the previous lactation with a LOW SCC (<200,000 cells/ml) that also started the new lactation with a LOW SCC (<200,000 cells/ml). |
| ZC. Percentage Low at end of previous lactation (SCC<200,000 cells/ml) | Of re-calving cows recorded starting a new lactation during the analysis period: The percentage that had a LOW SCC (<200,000 cells/ml) at the last milk recording in the previous lactation. |
| ZD. Percentage New SCC ≥200,000 cells/ml | The percentage of all recorded milk samples that were of the "New" SCC Category, namely the first HIGH SCC (≥200,000) in a lactation following one or more low SCC samples. |
| ZE. Percentage Dried-off with no SCC ≥200,000 cells/ml | The percentage of cows completing a lactation without recording a high SCC (cows recording only LOW SCC samples (<200,000 cells/ml) in the previous lactation). |
| ZF. Threshold Index new high / new low | Of cows with consecutive milk records in the same lactation, the number of cows changing from Low SCC at the previous to High SCC at the next recording divided by the number of cows going from High SCC at the previous to Low SCC at the next recording. |

| Parameter | Description |
|---|---|
| ZG. Recovery percentage of new/first/repeat infections | Of HIGH SCC cows (≥200,000cells/ml) that at the previous recording were either low SCC or not yet in milk, the percentage that were LOW SCC (<200,000 cells/ml) at the following recording. |
| ZH. Recovery percentage of chronic infections | Of CHRONIC High SCC cows (High SCC cows that at the previous recording were also High SCC), the percentage of those milked that were LOW SCC (<200,000 cells/ml) at the following recording. |
| ZI. Percentage drying off with no mastitis cases | The percentage of cows completing a lactation without recording a mastitis case. |
| ZJ. Mastitis rate (cases/100 cows in milk per year) | The total cow cases of mastitis recorded divided by the average population of cows in milk, represented as a % (cases/100 cows in milk). |
| ZK. Index mastitis case by Day 30 | The percentage of cows calving during the12 month period that recorded a mastitis case by day 30 of the lactation. |
| ZL. Index mastitis rate after Day 30 | The incidence rate of ***index*** mastitis cases in cows that have passed 30 days since calving. |

# Appendix 3. Herd Companion high SCC categories

The web-based Herd Companion program (**www.nmr.co.uk/Herd-companion**) was introduced by NMR in 2003 primarily to support the use of milk recording data to control somatic cell counts (SCC) in dairy herds.

Herd Companion focuses more on the duration of a high SCC infection rather than the magnitude of an individual milk sample. Using a threshold of 200,000 cells/ml milk to indicate infection, the program aims to balance the ability of many cows to self-cure with the need to assist cows where infection is becoming established. While in the region of 50% of cows self-cure after an initial raised SCC this recovery rate falls to less than 20% once a cow has recorded a second high SCC. It is these persistent high SCC cows that require attention before they are damaged irretrievably by a sustained period of infection.

The development of Herd Companion led to the definition of four main categories of high cell count cow, as illustrated below. Each vertical bar represents the magnitude of the SCC at each milk recording in a lactation. Where the bar is black the SCC is below the threshold of 200,000 cells/ml milk. A red bar indicates a SCC level above the threshold.

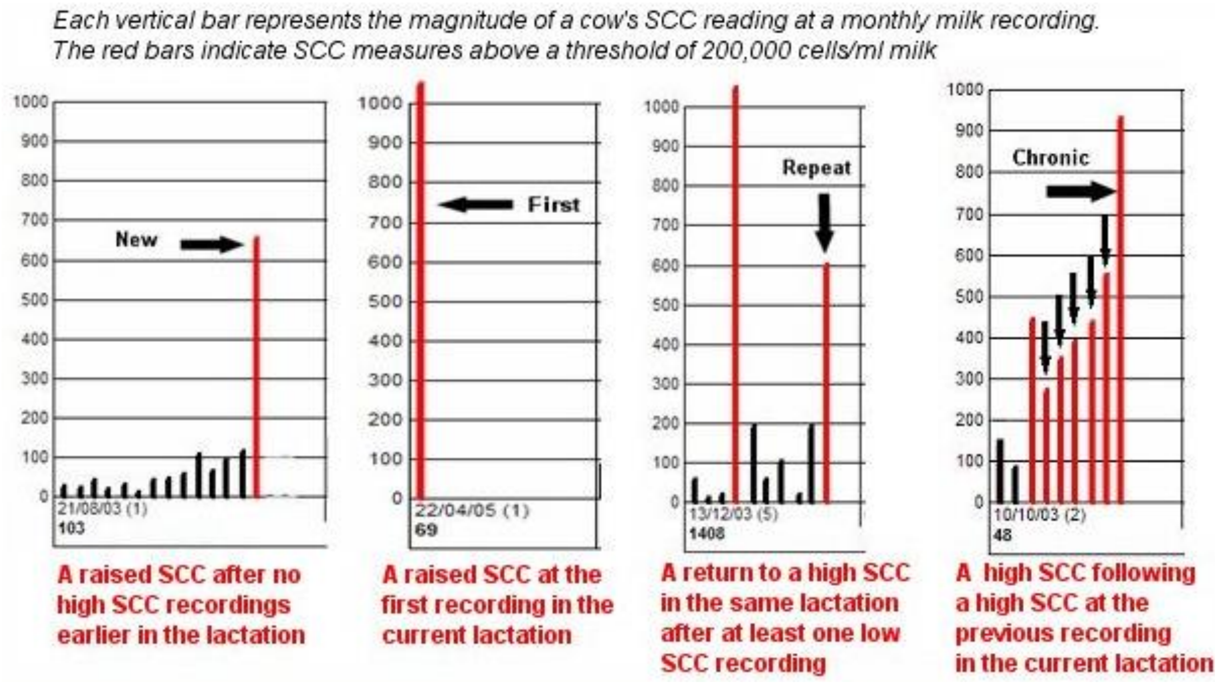


**NEW:** The "New" category describes cows recording their first high SCC in the lactation, having recorded one or more low SCCs at earlier recording(s). An infection acquired in the lactation.
**FIRST:** The "First" category describes cows that are HIGH SCC at their First milk recording in the current lactation. This is an infection that may be related to the dry period.
**REPEAT:** The "Repeat" category describes a possible re-infection (or failure to cure). A cow that had high SCC recording(s) earlier in the current lactation recorded a LOW SCC in the previous month(s) but has returned to a High SCC at the latest recording.
**Chronic:** The "Chronic" category describes a cow that is High SCC at the latest recording AND was also High SCC at the PREVIOUS recording(s). So she was high SCC last time and failed to recover. In the example above the cow has 7 consecutive high SCC recordings so has been defined as Chronic for the last 6 months of consecutive high SCC recordings.
