# Supplementary material for: Setting the boundaries–an approach to estimate the Loss Gap in dairy cattle
Source: PLoS One. 2024 Jun 27;19(6):e0306314. doi: 10.1371/journal.pone.0306314 (PMC11210862; doi:10.1371/journal.pone.0306314)
Supplement: Attachment — Submitted filename: Response to reviewers_27.05.2024.docx [file pone.0306314.s006.docx]

For ease of reading the comments/request made were left in **bold**.

**Reviewer 1**

**Could you please confirm that you have no identifying data in your supporting information file?**

Yes, the authors confirm that the data made available in the publication does not include any element that might compromise participant privacy.
